# Global RNA sequencing reveals that genotype-dependent allele-specific expression contributes to differential expression in rice F1 hybrids

**DOI:** 10.1186/1471-2229-13-221

**Published:** 2013-12-21

**Authors:** Gaoyuan Song, Zhibin Guo, Zhenwei Liu, Qin Cheng, Xuefeng Qu, Rong Chen, Daiming Jiang, Chuan Liu, Wei Wang, Yunfang Sun, Liping Zhang, Yingguo Zhu, Daichang Yang

**Affiliations:** 1 State Key Laboratory of Hybrid Rice and College of Life Sciences, Wuhan University, Luojia Hill, Wuhan, Hubei Province 430072, China

**Keywords:** Allele-specific expression, Complementary effects, Differentially expressed genes, Genotype-dependent monoallelic expression, Rice hybrids

## Abstract

**Background:**

Extensive studies on heterosis in plants using transcriptome analysis have identified differentially expressed genes (DEGs) in F_1_ hybrids. However, it is not clear why yield in heterozygotes is superior to that of the homozygous parents or how DEGs are produced. Global allele-specific expression analysis in hybrid rice has the potential to answer these questions.

**Results:**

We report a genome-wide allele-specific expression analysis using RNA-sequencing technology of 3,637–3,824 genes from three rice F_1_ hybrids. Of the expressed genes, 3.7% exhibited an unexpected type of monoallelic expression and 23.8% showed preferential allelic expression that was genotype-dependent in reciprocal crosses. Those genes exhibiting allele-specific expression comprised 42.4% of the genes differentially expressed between F_1_ hybrids and their parents. Allele-specific expression accounted for 79.8% of the genes displaying more than a 10-fold expression level difference between an F_1_ and its parents, and almost all (97.3%) of the genes expressed in F_1_, but non-expressed in one parent. Significant allelic complementary effects were detected in the F_1_ hybrids of rice.

**Conclusions:**

Analysis of the allelic expression profiles of genes at the critical stage for highest biomass production from the leaves of three different rice F_1_ hybrids identified genotype-dependent allele-specific expression genes. A cis-regulatory mechanism was identified that contributes to allele-specific expression, leading to differential gene expression and allelic complementary effects in F_1_ hybrids.

## Background

Heterosis, or hybrid vigor, refers to the superior biological functions of F_1_ hybrids compared with their parental homozygous or inbred lines. This phenomenon was first described by Charles Darwin and was later independently rediscovered by George H. Shull and Edward M. East in 1908 [1-3]. Although it is not well understood at the molecular level, heterosis has been exploited over the past half-century in plant and animal breeding. Two classic hypotheses, “dominance” and “overdominance”, have been proposed to explain hybrid vigor. The “dominance” hypothesis proposes that the detrimental allele from one parent is complemented by the superior allele from the other parent, and that the accumulated superior alleles in the F_1_ hybrids give rise to heterosis. By comparison, the “overdominance” hypothesis signifies that hybrid vigor results from the interaction between alleles brought together in the hybrid [4].

To attempt to discriminate between these hypotheses, extensive studies of gene effects and transcriptomics have been conducted [5-11]. Genetic analyses have revealed the genetic effects of additive, overdominance, dominance, and epistasis, and that interactions between different loci are associated with heterosis in different varieties [5-11]. Several studies analyzing transcription have indicated that DEGs commonly occur in inbred lines as well as between F_1_ hybrids and their parents. For example, 4–18% of maize genes are differentially expressed in different tissues of the maize inbred lines B73 and Mo17 according to microarray-based analyses [12]. In *Arabidopsis* seedlings, high-density single nucleotide polymorphism (SNP) analysis showed that 31% of all analyzed genes were differentially expressed between the parental inbred lines [13], while, in another study, 10.6% of genes were differentially expressed in different tissues of the hybrid rice LYP9 and its parents [14].

Recently, high-throughput RNA-sequencing technology revealed that 4-week-old shoots of the 93–11 and Nipponbare rice varieties had 24.0% DEGs, as did their reciprocal hybrids [15]. Moreover, a newly published global survey based on RNA sequencing technology found that approximately 70% of all expressed genes were differentially expressed between the two maize inbred parents B73 and Mo17, and that 42–55% were differentially expressed between the reciprocal F_1_ and its parents [16]. Although the molecular basis of heterosis has been attributed to the DEGs in the above hybrids, the underlying mechanism(s) causing differential expression remain unknown.

Several studies have shown that only one allele is expressed in heterozygotes [17-20], and monoallelic expression or an imbalance in heterozygote allelic expression has been extensively studied in humans and other mammals [21-23]. Transcription profile analyses have indicated that monoallelic expression could be caused by X-chromosome silencing, autosomal imprinting, or random events. Some studies with vegetative tissues from maize F_1_ hybrids identified several genes exhibiting allele-specific expression (ASE) [12,24,25], which differed markedly between the different F_1_ hybrids and was altered in response to environmental stress. This could contribute to heterosis.

The objectives of the present study were to explore global ASE in hybrid rice and to reveal the mechanism of differential expression in F_1_ hybrids using RNA sequencing. Three elite rice varieties were chosen that met the breeding objectives from different periods in China, Guangluai #4 (GL, 1970–1980s), Teqing (TQ, 1980–1990s), and 93–11 (1990s to present), plus their F_1_ hybrids, which we show have different levels of heterosis. Two F_1_ hybrids, GL × TQ and GL × 93-11, exhibited high heterosis, and the third, 93-11 × TQ, low heterosis. To obtain sufficient SNPs to distinguish the maternal and paternal alleles in F_1_ hybrids, the genomes of the three parents were re-sequenced. To identify more SNPs for further ASE analysis, nuclear RNA was extracted from leaves of the three F_1_ hybrids and their parents and subjected to Illumina RNA-Seq technology. We identify a global ASE profile that reveals a potential mechanism for an increased biomass-based, grain-yield heterosis.

## Results

### Global ASE analysis by RNA sequencing

To obtain sufficient SNPs for ASE analysis, we achieved a rice genome coverage of 17.7–27.7 fold to satisfy the minimum requirement of obtaining more than 90% SNPs [26]. A total of 76.2–119.0 million reads (100 bp per read) from three rice varieties serving as parents for the F_1_s were obtained using Illumina DNA-Seq technology (Additional file 1: Table S1), and 375,744–411,571 SNPs were detected between each parent with 98.0% accuracy (Additional file 2: Figure S1A, Additional file 3: Table S2). A total of 89.5–114.1 million reads (90 bp per read) were obtained from analogous tissue from the three F_1_ hybrids. Analysis disclosed that 29,064–29,928 genes at the secondary branch differentiation stage were expressed in the three F_1_ hybrids and their parents (Additional file 1: Table S1). The expression levels of 30 genes in the F_1_ hybrids and their parents were also determined, and quantitative real time polymerase chain reaction (qRT-PCR) and RNA-sequencing were shown to be highly correlated in terms of their analyses (expression level correlation coefficient *r* = 0.92-0.99 (Additional file 4: Table S3)). We found that 41,416–43,685 of the SNPs located in gene bodies had an average read coverage of 8.6–10.9 in the F_1_ hybrids. These SNPs were available for allelic expression analysis (Additional file 2: Figure S1B and Additional file 5: Table S4). Of these, 9,752–10,818 genes accounting for 33.5–36.1% of the total number of expressed genes containing SNPs could be used for distinguishing the alleles (Additional file 6: Figure S2). A total of 3,627–3,824 genes met the criterion of at least 10 SNP reads, so could be used for further ASE profile analyses.

**Figure 1:**
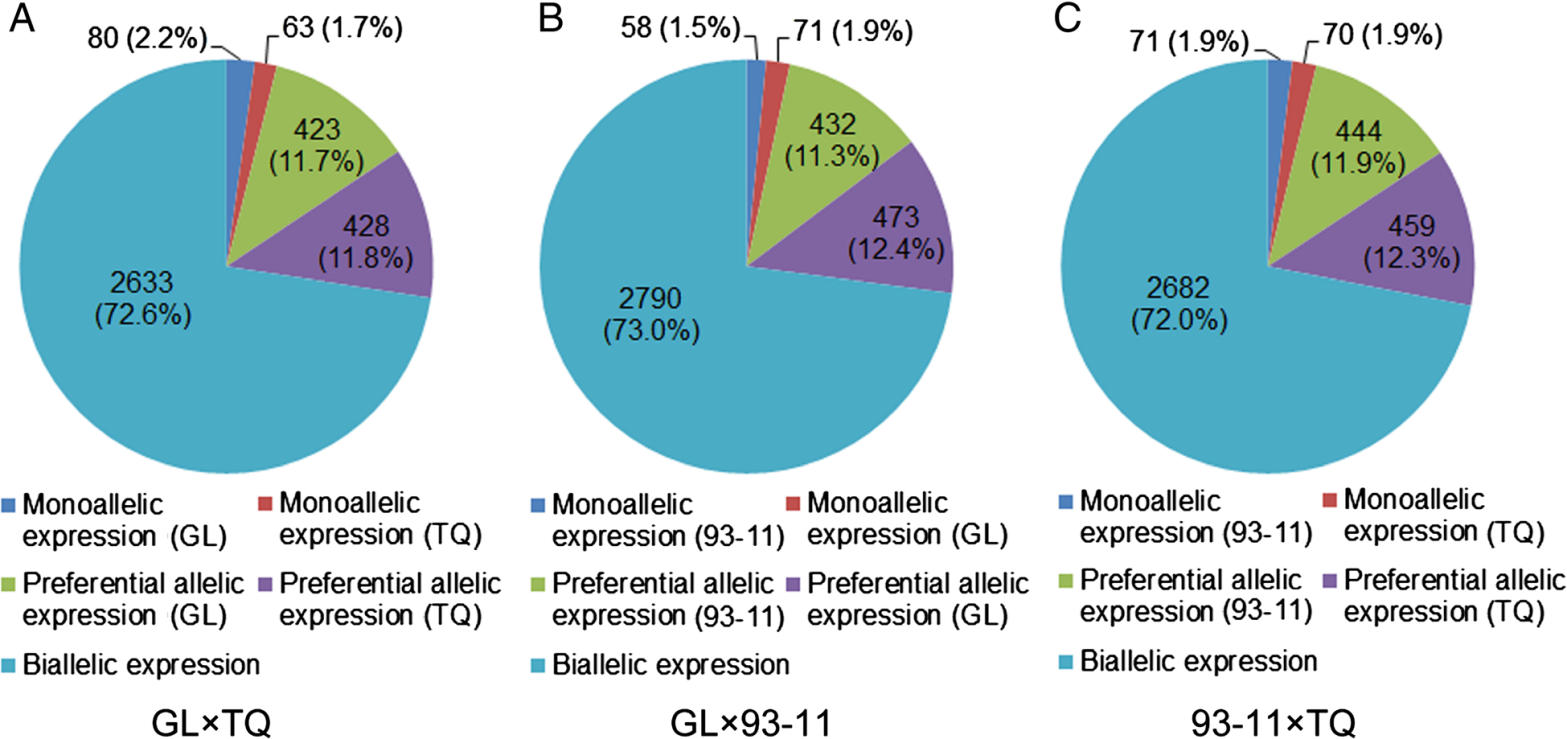
**Overall allele-specific expression (ASE) profiles in three F**_**1 **_**populations.** The three types of ASE genes and their proportions detected in GL × TQ **(A)**, GL × 93-11 **(B)**, and 93-11 × TQ **(C)**.

Through these ASE profile analyses, the F_1_ hybrids were classified into three categories: monoallelic expression, in which only one allele from either the maternal or paternal parent is expressed; preferential allelic expression, in which expression levels differ by more than two-fold between alleles; and biallelic expression, in which expression levels vary by less than two-fold between alleles (Additional file 7: Figure S3). We found that 3.4–3.9% of the genes were monoallelic expression, 23.5–24.2% were preferential allelic expression, and 72.0–73.0% were biallelic expression (Figure 1A-C). Further analysis of paternally and maternally derived read coverage in each heterozygote showed that the two parents contributed equally to the F_1_ hybrids. Our results are consistent with those from *Arabidopsis* embryos [27], and suggest that no obvious parent-of-origin effect occurred in the vegetative tissues of the rice hybrids (Figure 1A–C and Additional file 8: Figure S4).

### A cis-regulation mechanism and genotype-dependent monoallelic expression

**Figure 2:**
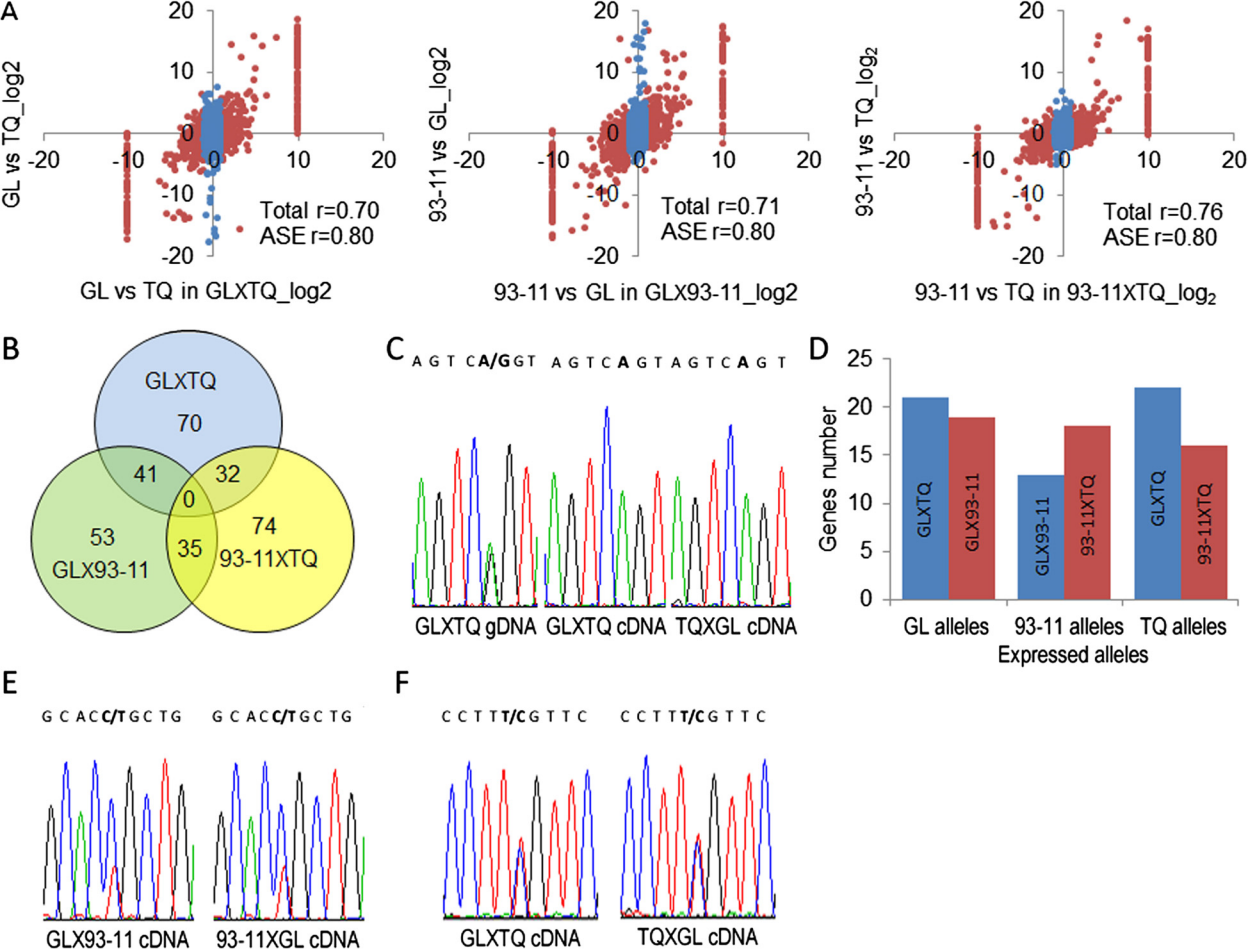
**The cis-regulatory mechanism and genotype-dependent monoallelic expression. (A)** Correlation analysis between genic expression in the parental generation and allelic expression in the F_1_ generation of the three hybrids. Red, allelic-specific expressed genes (ASE); blue, biallelic expressed genes. **(B)** The number of variety-specific and commonly expressed monoallelic expressed genes in the three F_1_ hybrids. **(C)** Example of a monoallelic expression gene confirmed by RT-PCR sequencing of reciprocal F_1_ crosses. **(D)** Confirmation of genotype-dependent monoallelic expression patterns in the three F_1_ hybrids showing the origin of the alleles. **(E)** Example of a preferential allelic expression gene confirmed by RT-PCR sequencing of reciprocal F_1_ crosses. **(F)** Example of a biallelic expression gene confirmed by RT-PCR sequencing of reciprocal F_1_ crosses.

To understand the relationship between gene expression in the parents and ASE in the F_1_ hybrids, the correlation between these parameters was determined for 3,627–3,824 genes, yielding correlation coefficients in the range of 0.70–0.76 (Figure 2A, *P* < 2.2E-16). A higher correlation coefficient was obtained from the same analysis with 2,026 ASE genes in the F_1_ hybrids (Figure 2A, Pearson’s *r* > 0.80, *P* < 2.2E-16). These results suggest that a cis-regulatory mechanism is occurring, that is, if the allele is transcribed in the parent it is also transcribed in its F_1_ hybrids, and if not in the parent then not in its F_1_ hybrids. Our data also indicate that only 5.4–19.3% of gene expression in F_1_ hybrids is non-additive. Trans-acting regulation may thus also contribute to the regulation of gene expression in rice hybrids, but would not have a major effect.

We identified 413 monoallelic expression genes in the three F_1_ hybrids (143 from GL × TQ, 129 from GL × 93-11, and 141 from 93-11 × TQ) (Additional file 9: Table S5). Of these, 108 were common to two F_1_ hybrids, but none were common to all three (Figure 2B). We randomly chose 134 monoallelic expression genes, accounting for 32.4% of the total, from the three hybrids and used reverse-transcription (RT)-PCR sequencing to verify a reliability of 91.8% (123/134) (Figure 2C, Additional file 10: Table S6). Through further investigation of allelic expression patterns in three reciprocal crosses, GL × 93-11 and 93-11 × GL, GL × TQ and TQ × GL, and 9311 × TQ and TQ × 93-11, we detected 109 monoallelic expression genes and 14 preferential allelic expression genes and found that all monoallelic expression and preferential allelic expression genes tested exhibited a genotype-dependent expression pattern, while 17 biallelic expression genes showed no difference between reciprocal crosses (Figure 2C–F, Additional file 11: Table S7). This shows that, regardless of the paternal or maternal origin in the reciprocal crossings, monoallelic expression and preferential allelic expression genes always express the allele from a given parent (Figure 2C and E). Combining our results with previous observations from maize [24], we suggest that a hitherto overlooked type of monoallelic expression occurs in eukaryotic organisms.

### ASE results in transcriptome divergence in the F_1_ hybrids

**Figure 3:**
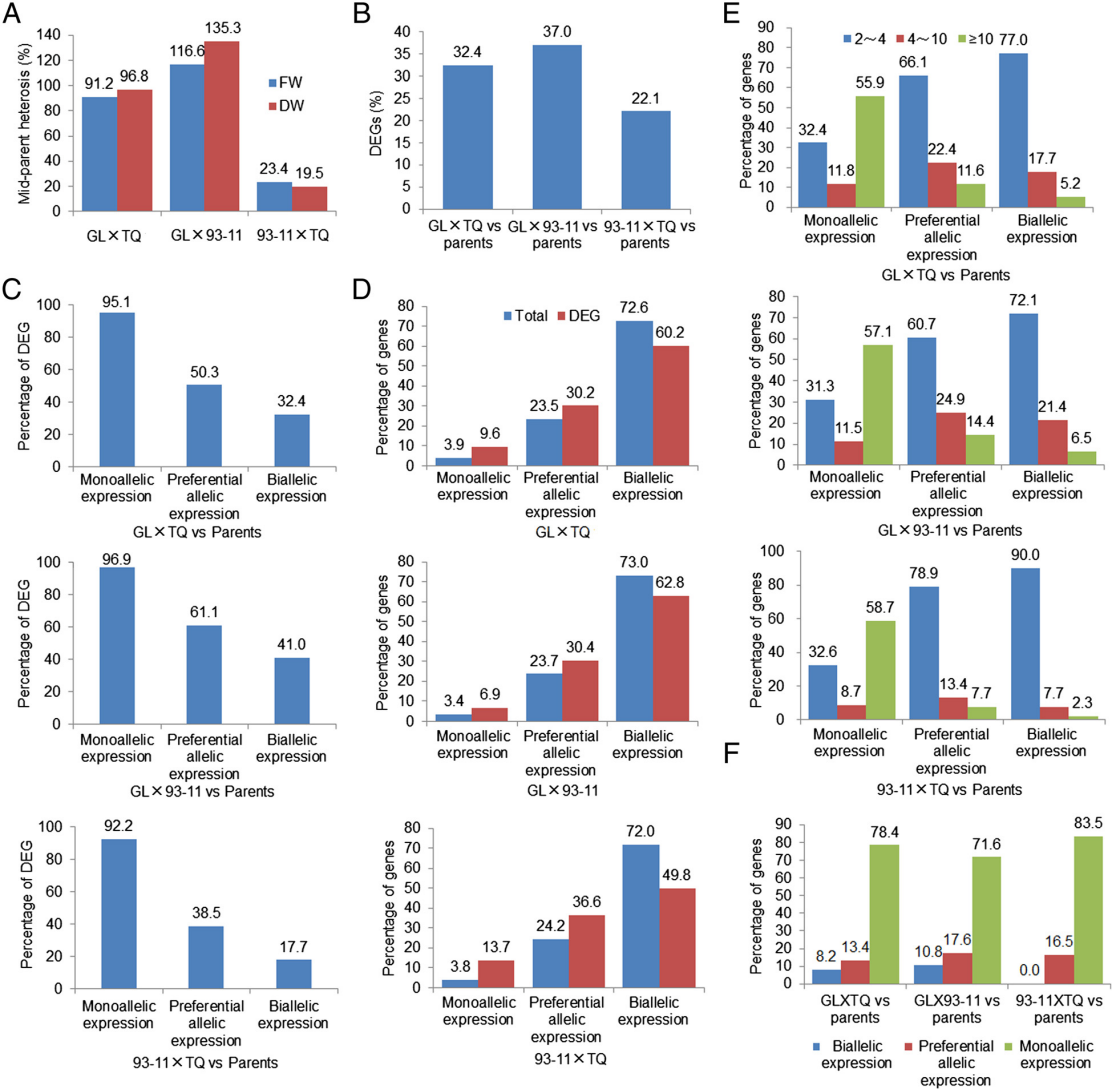
**Contributions of the three allelic expression types of genes to the differentially expressed genes (DEGs). (A)** The mid-parent heterosis level of total biomass at the secondary branch differentiation stage exhibited by the three F_1_ hybrid populations. Heterosis was evaluated using the fresh weight and dry weight of single plants from each F_1_ hybrid generation and their parents. **(B)** The preferential allelic expression genes compared between each F_1_ hybrid and its parents. **(C)** The percentages of DEGs represented by the genes of each allelic expression type. **(D)** The contributions of monoallelic expression, preferential allelic expression, and biallelic expression genes to the total genes and DEGs in the three F_1_s. **(E)** The proportions of genes with monoallelic expression, preferential allelic expression, and biallelic expression profiles in the F_1_ generation compared with their parents, sub-grouped according to fold differences in expression level. **(F)** The proportions of monoallelic expression, preferential allelic expression, and biallelic expression genes expressed in the F_1_ generation and one of the two parents but not expressed in the other parent.

Previous studies have demonstrated that higher levels of heterosis are associated with greater differences between the agronomic and/or metabolic traits of parents [28-30], and that DEGs (fold change >2.0, false discovery rate (FDR) <0.05) between F_1_ hybrids and their parents are among the major factors leading to heterosis [14,31,32]. To ascertain the extent to which monoallelic expression and preferential allelic expression genes contribute to heterosis, we dissected the global DEGs between F_1_ hybrids and their parents. The total number of DEGs in the three hybrids was correlated with heterosis level of both fresh and dry mass (*r* > 0.99, *P* < 0.03; Figure 3A–B).

To explore which mechanism(s) create DEGs in heterozygotes, we found that 95.1% of monoallelic expression genes, 50.3% of preferential allelic expression genes, and 30.4% of biallelic expression genes are DEGs (Figure 3C). Surprisingly, we discovered that the monoallelic expression and preferential allelic expression genes, comprising 27.5% of the total analyzed genes, amounted to 42.7% of the DEGs (Figure 3D). More specifically, the monoallelic expression genes, accounting for 3.7% of the total analyzed genes, gave rise to 10.0% of the DEGs, the preferential allelic expression genes (23.8%) amounted to 32.4%, and the biallelic expression genes (72.3%) were composed of only 57.6% of the DEGs (Figure 3D). By determining contributions to DEGs between F_1_ hybrids and their parents, we found that 57.2% of DEGs from monoallelic expression, 11.4% from preferential allelic expression, and 3.9% from biallelic expression exhibited a considerably greater than 10-fold difference. By contrast, 79.7% of DEGs from biallelic expression, 68.6% from preferential allelic expression, and 32.1% from monoallelic expression genes displayed a less than four-fold difference between the F_1_ and their parents (Figure 3E). The 71.6–83.5% of the genes that were expressed in the F_1_s but silenced in one of the two parents showed monoallelic expression patterns (Figure 3F). These results demonstrate that the majority of DEGs (>10 fold difference) are attributable to ASE, and that monoallelic expression genes in particular play an important role in gene expression divergence between F_1_ hybrids and their parents.

### Complementary effects are mainly contributed by ASE genes

**Figure 4:**
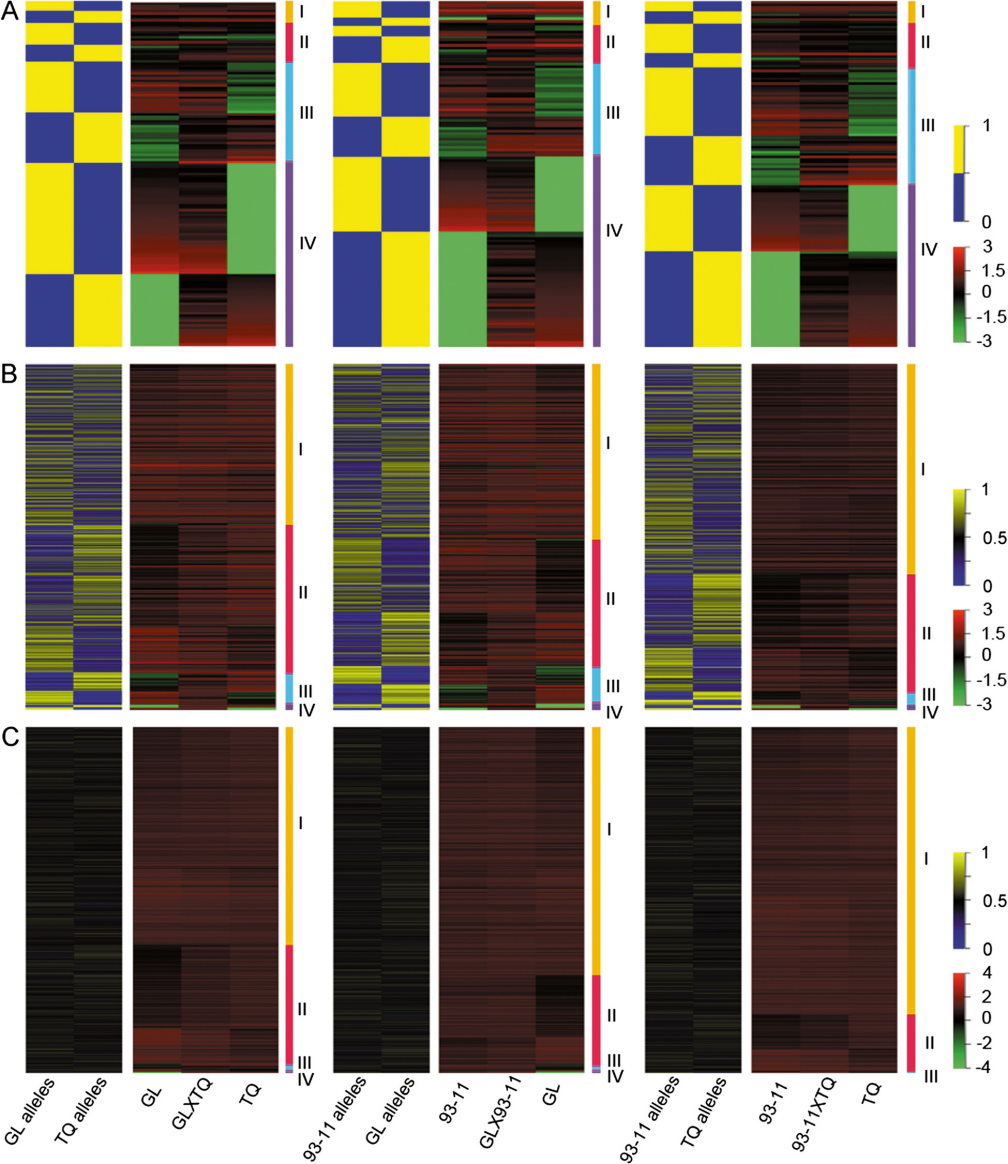
**Complementary effects of allele-specific expression genes contributing to transcriptome optimization of F**_**1 **_**hybrids.** The left two lanes of each panel show the allelic expression patterns observed in the F_1_s, whereas the right three lanes compare the expression levels of these genes in the hybrids (lane 4) to those in the parents (lanes 3 and 5). The genes in groups I, II, III, and IV are those with no difference between the parents, a 2- to 10-fold difference between the parents, a greater than 10-fold difference between the parents, and expression in only one parent, respectively. **(A)** The expression levels of monoallelic expression genes in the F_1_ hybrids and their parents. **(B)** The expression levels of preferential allelic expression genes in the F_1_ hybrids and their parents. **(C)** The expression levels of biallelic expression genes in the F_1_ hybrids and their parents. The short bands on the same horizontal line indicate the same gene. The expression level of each gene was normalized by log_10_. The vertical bars on the right correlate color in the panels with relative levels of transcription.

Because the presence of a cis-regulatory mechanism of allelic expression would be in accordance with the “dominance” hypothesis, we analyzed the transcriptomic profiles of all ASE genes in the three F_1_ hybrids and their parents. A large number of genotype-dependent monoallelic expression genes in F_1_ hybrids would also be in accordance with the “dominance” hypothesis. The results showed that an average of 51.6% (53.1% in GL × TQ, 55.0% in GL × 93-11, and 46.8% in 93-11 × TQ) of genes expressed in one parent and non-expressed in the other were categorized as monoallelic expression genes in F_1_ hybrids (Figure 4A class IV and Additional file 12: Table S8), whereas 30.2% (29.4, 27.1, and 34.0% in GL × TQ, GL × 93-11, and 93-11 × TQ, respectively) of genes expressed at low levels in either parent, but enhanced in F_1_ hybrids, were categorized as monoallelic expression genes (Figure 4A class III and Additional file 12: Table S8). Most of both types of monoallelic expression genes exhibited a mid-parent expression level (Additional file 12: Table S8). Therefore, the alleles only expressed in the F_1_ were those expressed in the inbred parent, while the alleles silenced in the inbred parent were also silenced in F_1_. This means that the total expression level of the gene in the F_1_ is one half that in the parent, which is equivalent to a mid-parent expression level (Figure 4A class IV and Additional file 12: Table S8). This dosage effect implies that a cis-regulatory mechanism is acting.

Moreover, we found that more monoallelic expression genes were downregulated than upregulated in F_1_ (Additional file 13: Table S9), and that 9.7% of preferential allelic expression genes exhibited the same patterns as monoallelic expression genes in the F_1_ hybrids (Figure 4B classes III and IV). The analysis of genes within the categories of activated and enhanced expression in F_1_ hybrids found complementary effects of superior alleles from both parents with average values of 73.8% and 93.6% for class III and class IV genes, respectively. By contrast, a biallelic expression pattern was exhibited by only 26.2% and 6.4% in class III and class IV genes, respectively (Figure 4C and Additional file 14: Table S10). The proportion of genes with complementary effects in GL × TQ and GL × 93-11 was higher than that in 93-11 × TQ (7.8% and 7.5% versus 4.7%, respectively), which was consistent with the heterosis level of these F_1_ hybrids (Additional file 14: Table S10). Our data imply that ASE genes, most notably monoallelic expression genes, are the main contributors to allelic complementary effects in hybrid rice.

### ASE genes have diverse biological functions in F_1_ hybrids

To ascertain the molecular and biological functions of monoallelic expression and preferential allelic expression genes, we performed gene ontology (GO) analysis. Four GO terms for molecular functions, nucleotide binding, receptor activity, protein binding, and kinase activity, were commonly found among both monoallelic expression and preferential allelic expression genes (Table 1 and Additional file 15: Tables S11 and Additional file 16: Table S12). These functions indicate that monoallelic expression genes are mainly involved in protein modification, signal transduction, and response to endogenous stimulus pathways. A wider diversity of functions, including biosynthesis, morphogenesis, and carbohydrate metabolism, was found for preferential allelic expression genes (Table 1 and Additional file 17: Tables S13 and Additional file18: Table S14), suggesting that ASE genes have important roles in biosynthesis, development, and their regulation. To distinguish between the enrichment of monoallelic expression and preferential allelic expression gene functions and that of all genes for ASE analysis, we performed GO analysis of 3,627–3,824 genes in three F_1_ hybrids. Limited GO terms were common to monoallelic expression and preferential allelic expression genes in different F_1_ hybrids (Additional files 19: Figures S5 and Additional file 20: Figure S6), which might be a consequence of specific monoallelic expression and preferential allelic expression genes occurring in different F_1_ hybrids, suggesting that allele-specific expression genes have different roles in different genomic backgrounds.

**Table 1:** The common functions of monoallelic expression and preferential allelic expression genes in the three rice hybrids

	**GO Term**	**GL × TQ**		**GL × 93-11**		**93-11 × TQ**	
		**Genes**	**P value**	**Genes**	**P value**	**Genes**	**P value**
	**Molecular function**						
Monoallelic expression genes	Nucleotide binding	26	1.87E-08	27	2.20E-10	37	1.52E-14
	Receptor activity	8	3.77E-05	12	1.23E-09	7	5.85E-04
	Protein binding	25	5.51E-10	41	1.70E-26	46	1.98E-26
	Kinase activity	22	5.65E-09	22	4.85E-10	28	3.30E-12
Preferential allelic expression genes	Nucleotide binding	252	4.38E-48	300	1.27E-66	220	3.26E-30
	Receptor activity	59	3.55E-18	77	2.05E-28	25	0.02
	Protein binding	231	1.09E-55	276	4.19E-76	185	3.07E-29
	Kinase activity	180	7.10E-37	235	1.27E-62	142	8.13E-18
	Transcription factor activity	163	1.20E-27	127	3.67E-11	158	2.33E-23
	Structural molecule activity	63	4.19E-11	89	2.71E-22	59	1.02E-08
	Transporter activity	112	2.22E-14	105	5.37E-10	119	1.25E-15
	Carbohydrate binding	7	0.03	9	0	8	0.01
	**Biological function**						
Monoallelic expression genes	Protein modification	27	3.10E-11	27	1.35E-12	36	9.22E-17
	Signal transduction	31	3.16E-13	27	1.48E-11	43	2.53E-21
	Response to endogenous stimulus	28	8.58E-10	24	2.49E-08	40	6.32E-17
Preferential allelic expression genes	Protein modification	221	2.89E-48	235	4.77E-50	175	8.30E-24
	Signal transduction	238	6.76E-50	266	1.24E-58	182	1.07E-21
	Biosynthesis	125	3.00E-10	147	1.38E-14	90	0.023505
	Morphogenesis	32	2.70E-10	39	7.50E-14	23	4.21E-05
	Response to endogenous stimulus	264	1.63E-54	265	1.75E-48	207	3.00E-25
	DNA metabolism	49	4.49E-05	42	0.010399	46	7.96E-04
	Protein biosynthesis	40	2.48E-06	51	5.48E-10	45	8.44E-08
	Amino acid and derivative metabolism	86	6.11E-15	79	2.05E-10	61	3.23E-05
	Lipid metabolism	67	7.99E-11	63	5.17E-08	45	0.003092
	Response to stress	172	2.38E-24	165	2.76E-18	204	5.86E-36
	Catabolism	76	1.96E-19	43	3.72E-04	58	1.06E-09
	Response to external stimulus	58	5.92E-11	56	6.79E-09	62	5.63E-12
	Response to biotic stimulus	107	1.36E-13	143	4.41E-26	149	2.22E-30
	Response to abiotic stimulus	141	9.70E-23	140	2.49E-19	146	4.98E-23
	Pollination	10	0.001166	20	2.35E-10	11	4.48E-04
	Flower development	30	3.36E-06	44	1.54E-12	42	8.04E-12
	Cell organization and biogenesis	39	1.81E-04	33	0.019878	31	0.033566
	Cell differentiation	44	2.43E-11	51	4.52E-14	36	6.69E-07
	Secretory pathway	81	0.003356	93	2.74E-04	90	3.59E-04

## Discussion

Previous studies of transcriptome analyses related to heterosis have mainly been conducted using microarray analyses, and RT-PCR and RNA-sequencing technology [15,31,33]. Here, we combined RNA-sequencing with DNA re-sequencing technology to establish ASE assays and achieved a 20-fold coverage of rice genome re-sequencing data to identify SNPs. A strict statistical cut-off for SNP calling enabled fully quantitative analyses of both overall and allele-specific gene expression profiles to be obtained for rice leaves at the stage of secondary branch differentiation. These data were verified by PCR-sequencing and RT-PCR sequencing using analogous materials planted in the following year. This developing stage is important as grain yield is directly correlated with the biomass established early in vegetative growth [34]. SNP accuracies of 98.0% and 91.8% were confirmed, indicating the reliability of both genome and transcriptome data, respectively.

Monoallelic expression genes have been studied for nearly a half century in humans and other mammals [17-20,35,36]. Most cause X-chromosome silencing, autosomal imprinting, or random events [17-20,35,36], and contribute to dose-dependent gene expression, immune responses, and disease biogenesis, including several types of cancers [17-20,35-37]. ASE and regulation mechanisms have also been studied in humans and other animals [23,38,39]; however, by contrast, monoallelic expression is poorly understood in higher plants. Different studies have reported a high proportion of ASE genes in maize hybrids (50% and 60%) with cis-regulatory effects underlying the ASE [24,40], compared with a more limited number of monoallelic expression genes [25]. Other studies using F_1_ hybrids of *japonica*-*indica*, maize, and *Arabidopsis* focused on endosperm-localized genes and identified more than 100 imprinted genes [41-44]. However, no large-scale ASE analysis has previously been carried out in rice hybrids.

We identified 413 monoallelic expression and 2,659 preferential allelic expression genes in the three F_1_ hybrids via a global transcriptome analysis. Of the total genes analyzed, 3.4–3.9% exhibited monoallelic expression and 23.5–24.2% exhibited preferential allelic expression patterns. The proportion of ASE genes, moreover, did not differ significantly in the three F_1_ hybrids, which is similar to a previous report in maize hybrids [24]. Importantly, our results indicated that all monoallelic expression and preferential allelic expression genes tested exhibited genotype-dependent expression patterns in reciprocal crosses, which contrasts with the findings of monoallelic expression genes in humans and other mammals [21,45]. The observed genotype-dependent ASE in the vegetative tissue of hybrid rice could represent a common mechanism of allelic complementary effects in hybrids, and show the importance of parental genotype in both crossbreeding and hybrid breeding. Given the number of genes with genotype-dependent monoallelic expression involved in a wide range of GO categories that play important biological functions, many potential opportunities exist for them to contribute to DEGs and to produce the diverse phenotypes of the F_1_ hybrids.

In the present study, the genotype-dependent ASE genes might confer a fitness advantage to the heterozygous state relative to either of their homozygous parents. As most monoallelic expression genes were silenced or suppressed in one of the parents, we speculate that genotype-dependent monoallelic expression might be the consequence of artificial parent selection by breeders. To meet the breeding objectives, “superior alleles” are accumulated in the elite parent for long-term selection (Figure 4A). Such alleles could maintain allelic diversity in different varieties and enlarge the allele difference in the rice germplasm. Therefore, our findings of allelic complementary effects in F_1_ hybrid rice could guide the selection of elite parents through complementing a variety of the superior alleles.

Previous studies have indicated that differential gene expression is common between F_1_ and parents, and that it is a major contributor to heterosis at the transcription level [14,31]. For example, hundreds of differentially expressed genes were detected at different developmental stages between the elite hybrid rice LYP9 and its parents [14], and analogous results were obtained from studies with *Arabidopsis* and maize [28,32,46,47]. Our study also found that the percentage of DEGs between F_1_ hybrids and parents exactly correlated with the heterosis level of each F_1_ hybrid (Figure 3A and B).

Such results do not divulge, nonetheless, how the alleles from inbred lines become the DEGs in heterozygotes, although the mechanism behind this is revealed to some extent by genome-wide, ASE analysis using high-throughput RNA sequencing. Moreover, in the present study, the most significant DEGs between the F_1_ and parent were expressed in the F_1_ but silenced in one of the parents, accounting for 93.7% of the DEGs associated with ASE. Of these, 15.8% showed preferential allelic expression and 77.8% were monoallelic expression. Altogether, 27.5% of the ASE genes contributed to 42.4% of the total number of DEGs. Although the proportion of ASE genes was similar in the three F_1_ hybrids, the total numbers of DEGs differed, with fewer detected in 93-11 × TQ (Figure 3B) with its lower heterosis level (Figure 3A) and more found in GL × TQ and GL × 93-11 with their higher heterosis levels. The same results were also found between their parents, suggesting that transcriptome divergence in F_1_ hybrids is attributed to transcriptome divergence between parents. Crucially, increased parent allelic variation could be an important strategy for maintaining higher heterosis levels in hybrid rice breeding programs through enlarging the transcriptome diversity between parents, which have accumulated different sets of superior alleles.

A recent study has provided molecular evidence for a single gene model to support the “overdominance” hypothesis of heterosis in tomato hybrids [48]. Because of technological limitations, however, the maternal and paternal alleles in the F_1_ hybrids could not be distinguished effectively. The recent development of high-throughput sequencing technology provides the opportunity to study ASE in heterozygotes. Many genes have exhibited additive expression patterns in F_1_ hybrids in previous studies, and complementary effects at the gene expression level have been reported in hybrid maize [16]. Our data derived from global allelic expression profiles extend this result to hybrid rice, and also reveal the mechanism of DEG formation in heterozygotes. Our observed high correlation (>0.7) between allelic expression in F_1_ hybrids and gene expression in parental lines indicates that a cis-regulated mechanism plays an essential role in allelic expression. Allelic complementation effects, moreover, can be the outcome of a cis-regulatory mechanism mainly contributed to by ASE genes.

The findings of the present study support the “dominance” hypothesis for *indica* hybrid rice varieties and reveal that the consequences of complementation, primarily by genotype-dependent monoallelic expression and preferential allelic expression alleles, are an accumulation of “superior” alleles that confer monoallelic expression and preferential allelic expression genes in F_1_ hybrids. The expression of these “superior” alleles offers an opportunity to optimize the transcriptomes that give rise to heterosis in F_1_ hybrids. Although our results revealed that allelic complementary effects played a major contribution to gene expression in hybrid rice and support the “dominance” hypothesis, they do not exclude the contribution of different mechanisms to heterosis in hybrid rice and other crops.

## Conclusions

Allelic expression profiles in hybrid rice determined by RNA-sequencing technology demonstrated a type of genotype-dependent monoallelic expression genes in plants. DEGs between parents and F_1_ hybrids were mainly attributable to ASE genes, which gave rise to the observed allelic complementary effects in F_1_ hybrids.

## Methods

### Plant material and phenotypic analysis

Reciprocal crosses were made between the three *indica* varieties, Guangluai #4 (GL), 93–11, and Teqing (TQ), at Hainan Island, China in the spring of 2009. The six reciprocal F_1_ hybrids were planted together with the three parents in Wuhan, China in the summer of 2009. Triplicate plots, each containing 30 individuals, were planted for all nine genotypes. Heterosis levels were evaluated by measuring the fresh and dry biomass of the above-ground parts of the plants at the secondary branch differentiation stage, which is critical for determining the highest biomass and maximum grain yield. The mid-parent heterosis level was calculated as described by Ma et al. [49]. Analogous leaves from the F_1_ hybrids and parents at the same development stages in 2010 were used to verify gene and allelic expression and genotype-dependent monoallelic expression.

### Nuclear RNA extraction

The second fully expanded leaves were harvested at the secondary branch differentiation stage, immediately frozen in liquid nitrogen and stored at -80°C. Material from the triplicate plots was pooled at harvest. Nuclei were isolated from ~10 g of frozen leaves using the Plant Nuclei Isolation/Extraction Kit (Sigma, St. Louis, MO, USA). Total hnRNA was extracted from nuclei using Trizol (Invitrogen, Carlsbad, CA, USA) according to the manufacturer’s instructions, and treated with RNase-free DNase I (New England Biolabs, Ipswich, MA, USA) to remove any contaminating genomic DNA.

### Library construction

The Illumina mRNA-Seq Sample Prep Kit (Illumina, San Diego, CA USA) was used to prepare the sequencing library with 3 μg of nuclear RNA. Fragmentation buffer in the kit was added directly to hnRNA to produce short fragments of 200–700 bp, which served as the templates for first-strand cDNA synthesis using random hexamers. Second-strand cDNA was synthesized followed the protocol described in the kit and was purified using a QIAquick PCR Extraction Kit (Qiagen, Valencia,CA USA) and eluted in elution buffer (EB). The short fragments were then ligated to sequencing adapters. Suitable fragments of approximately 200 bp were selected as templates for amplification in a MyCycler PCR instrument (Bio-Rad, Hercules, CA USA) with the following program: denaturation at 98°C for 30 s followed by 15 cycles of 98°C for 10 s, 65°C for 30 s, and 72°C for 30 s plus a terminal hold at 72°C for 5 min. The samples were then purified using the QIAquick PCR Purification Kit according to the manufacturer’s protocol and eluted in 30 μL of EB. A 1-μL aliquot of the construct was loaded onto an Agilent Technologies 2100 Bioanalyzer using the Agilent DNA 1000 Chip Kit (Agilent, Santa Clara, CA USA). After verifying the size and purity of the DNA fragments, the library was sequenced using an Illumina GA II x platform by BGI (Shenzhen, China).

The DNA Illumina TruSeq DNA sample preparation kit was used to prepare a genomic DNA library according to the manufacturer’s protocol. Contaminating RNA was removed by treating with RNase A. The DNA samples were sent to the Beijing Novo Gene Company (Beijing, China) for sequencing using an Illumina Hiseq 2000 platform.

### Read alignment

The raw reads were filtered before data analysis by removing reads consisting of adaptors only, those with greater than 10% unknown bases, and those in which more than half of the bases gave a quality score of less than 5.0. The remaining (clean) reads were mapped to the reference genome of *Japonica* variety Nipponbare (http://www.gramene.org/) using SOAP2 software [50]. Mismatches of no more than two bases were allowed in the alignment. We used the reads per kb per million reads (RPKM) method to calculate unique gene expression levels [51].

### SNP identification

SOAP2 was used to align each read to the Nipponbare reference genome (http://www.gramene.org/), with no more than three mismatches to the candidate SNPs permitted for each read [50]. This method of SNP calling has been previously described [46]. A statistical model based on Bayesian theory and the Illumina quality system was used to calculate the probability of each possible genotype at every position from the alignment of reads to the reference genome. Six criteria were set to filter out unreliable SNPs: 1) the read quality value must be no lower than 20, 2) the SNPs must be at least 5 bp from each other, 3) the SNP must be represented by at least four reads, 4) the sequencing depth must be less than 10,000, 5) the SNP must be more than 5 bp distant from an intron-exon junction and 6) the approximate copy number of the flanking sequences must be less than four. SNP sets from each biological replicate of GL versus TQ, GL versus 93–11, and 93–11 versus TQ were obtained and used for further allele-specific analysis.

### ASE analysis

The paternal and maternal alleles expressed in the F_1_ hybrid transcriptomes were distinguished by their SNP nucleotype. The expression level was calculated based on at least 10 reads of single genes. The expression level from a paternal or maternal allele was calculated based on the number of reads for the given allele divided by the total number of reads for the SNP. When only one allele was expressed, the gene was categorized as showing monoallelic expression. When the allele expression level was biased toward one parent by more than two-fold, the gene was categorized as showing preferential allelic expression. When the two alleles were expressed equally (less than two-fold difference), the gene was categorized as showing biallelic expression. In our computer analysis, the relative allele expression value of “0”, which occurred when an allele was not expressed, could not be computed. Therefore, to enable calculations, the “0” was replaced by 0.001.

### SNP confirmation and validation of allelic expression

For SNP validation, primers flanking the SNP sites were designed to amplify 300–800 bp fragments. PCR amplification was performed using reaction mixtures containing 10 ng of cDNA template and 5 μM primer. PCR was performed using the MyCycler PCR system with the following parameters: denaturation at 95°C for 5 min followed by 30 cycles of 95°C for 30 s, 50–55°C for 30 s, and 72°C for 30 s plus a terminal hold at 72°C for 5 min. PCR products were purified using the Axy PCR Cleanup Kit (AxyGEN Bioscience, Union city, CA USA) and sequenced using an ABI 3730xI machine. The resulting sequences were compared with reference SNPs detected by genome sequencing.

For monoallelic expression and preferential allelic expression validation, cDNA and gDNA from each F_1_ sample were used as PCR templates, and the sequences derived from the genome and transcriptome were compared. For preferential allelic expression and biallelic expression validation, cDNA from reciprocal F_1_ hybrids was used as PCR templates. The sequencing results for cDNA from reciprocal F_1_ hybrids were compared to detect parent-of-origin effects.

### GO and statistical analyses

GO analysis was performed using the open-source MAS3 database (http://bioinfo.capitalbio.com/mas3/). A threshold of a two-fold change in gene expression levels and a FDR of <0.05 were used to identify differentially expressed genes. The *P*-values and the FDRs of differentially expressed genes were calculated as described [52].

### Gene expression level validation

To validate the gene expression level, 30 randomly selected genes with different expression levels were verified by quantitative RT-PCR as described by Wang et al. [53].

## Abbreviations

ASE: Allele-specific expression; DEG: Differentially expressed gene; FDR: False discovery rate; GO: Gene ontology; RPKM: Reads per kb per million reads; RT-PCR: Reverse transcription polymerase chain reaction; SNP: Single nucleotide polymorphism.

## Competing interests

The authors declare that they have no competing interests.

## Authors’ contributions

GYS performed the most of the experiments; ZBG performed part of data analysis; GYS, ZWL, RC and CL did the monoallelic confirmation; DMJ performed all field experiments; ZWL, XFQ, WW, LPZ and QC helped GYS performed crossing and analysis of phenotypes; DCY and YGZ supervised this study; DCY and GYS designed the experiments and wrote the manuscript. All the authors discussed the results and contributed to the manuscript. All authors read and approved the final manuscript.

## Supplementary Material

Additional file 1: Table S1 Sequencing depth. Click here for file

Additional file 2: Figure S1 SNP locations (A) The location of SNPs detected between parents. (B) The location of SNPs used to evaluate allele-specific expression in each F_1_ hybrid. Click here for file

Additional file 3: Table S2 Confirmed SNPs. Click here for file

Additional file 4: Table S3 List of genes used for expression level confirmation. Click here for file

Additional file 5: Table S4 Read coverage in the ASE analysis. Click here for file

Additional file 6: Figure S2 Number of expressed genes containing SNPs in different F_1_ hybrids. Click here for file

Additional file 7: Figure S3 A model of the differential allelic expression pattern found in hybrid rice: biallelic expression, preferential allelic expression, and monoallelic expression. Click here for file

Additional file 8: Figure S4 The total read coverage of each allele from the two parents in the three F_1_ populations. Click here for file

Additional file 9: Table S5 List of all monoallelically expressed genes. Click here for file

Additional file 10: Table S6 List of confirmed monoallelically expressed genes. Click here for file

Additional file 11: Table S7 List of confirmed preferential allelic expression and biallelic expression genes. Click here for file

Additional file 12: Table S8 The expression levels of genes with monoallelic expression in the F_1_ and parental populations. Click here for file

Additional file 13: Table S9 The expression changes of monoallelic expression genes between F_1_ and parents. Click here for file

Additional file 14: Table S10 Allelic expression genes classified by the relative expression level of parents. Click here for file

Additional file 15: Table S11 Molecular function of monoallelic expression genes. Click here for file

Additional file 16: Table S12 Molecular function of preferential allelic expression genes. Click here for file

Additional file 17: Table S13 Biological function of monoallelic expression genes. Click here for file

Additional file 18: Table S14 Biological function of preferential allelic expression genes. Click here for file

Additional file 19: Figure S5 Enriched biological functions in different groups of genes in GL × TQ (A), GL × 93-11 (B), and 93-11 × TQ (C). Click here for file

Additional file 20: Figure S6 Enriched molecular functions in different groups of genes in GL × TQ (A), GL × 93-11 (B), and 93-11 × TQ (C). Click here for file
