# Supplementary material for: Global RNA sequencing reveals that genotype-dependent allele-specific expression contributes to differential expression in rice F1 hybrids
Source: BMC Plant Biol. 2013 Dec 21;13:221. doi: 10.1186/1471-2229-13-221 (PMC3878109; doi:10.1186/1471-2229-13-221)
Supplement: Additional file 1: Table S1 — Sequencing depth. [file 1471-2229-13-221-S1.docx]

Table S1. Sequencing depth

| Materials | | GL | 93-11 | TQ | GL×TQ | GL×93-11 | 93-11×TQ |
| --- | --- | --- | --- | --- | --- | --- | --- |
| Reads number | RNA | 113,770,636 | 106,192,798 | 114,053,786 | 89,532,864 | 105,211,962 | 110,543,858 |
|  | DNA | 76,237,504 | 119,006,988 | 113,493,466 | _ | _ | _ |
| Expressed gene | | 29,910 | 29,064 | 29,682 | 29,101 | 29,928 | 29,112 |
