# Supplementary figures and images for: Global RNA sequencing reveals that genotype-dependent allele-specific expression contributes to differential expression in rice F1 hybrids
Source: BMC Plant Biol. 2013 Dec 21;13:221. doi: 10.1186/1471-2229-13-221 (PMC3878109; doi:10.1186/1471-2229-13-221)

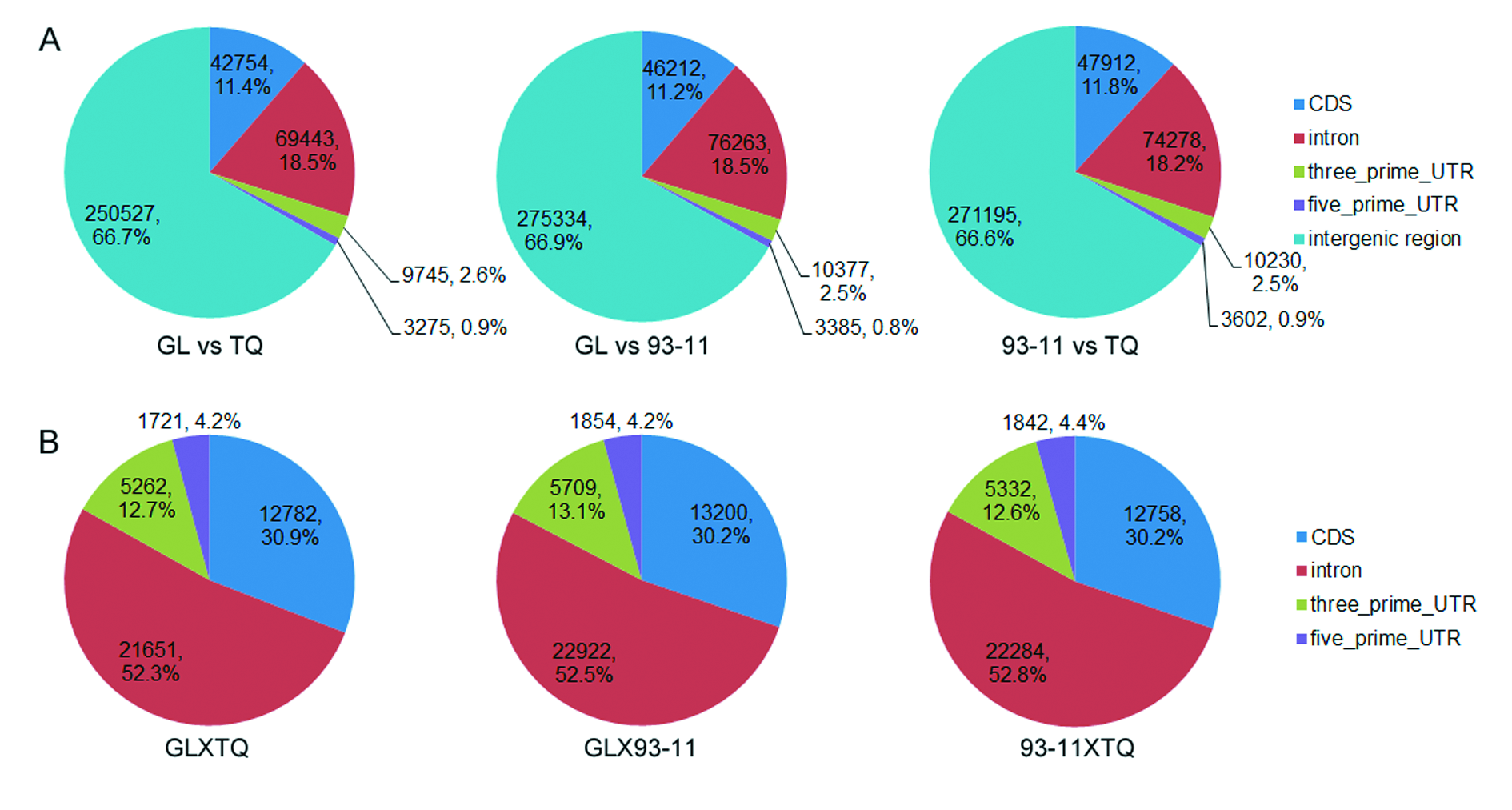

Supplement: Additional file 2: Figure S1 — SNP locations (A) The location of SNPs detected between parents. (B) The location of SNPs used to evaluate allele-specific expression in each F1 hybrid. [file 1471-2229-13-221-S2.tiff]

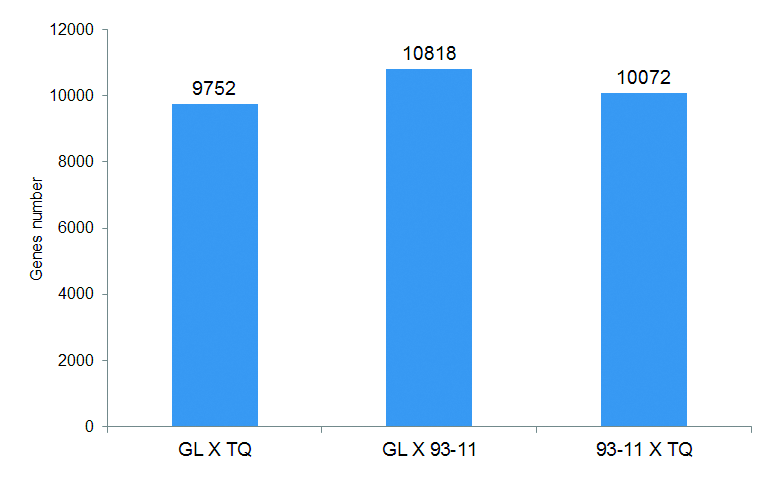

Supplement: Additional file 6: Figure S2 — Number of expressed genes containing SNPs in different F1 hybrids. [file 1471-2229-13-221-S6.tiff]

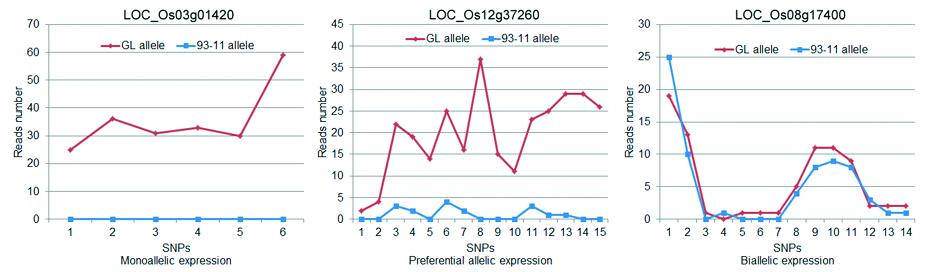

Supplement: Additional file 7: Figure S3 — A model of the differential allelic expression pattern found in hybrid rice: biallelic expression, preferential allelic expression, and monoallelic expression. [file 1471-2229-13-221-S7.tiff]

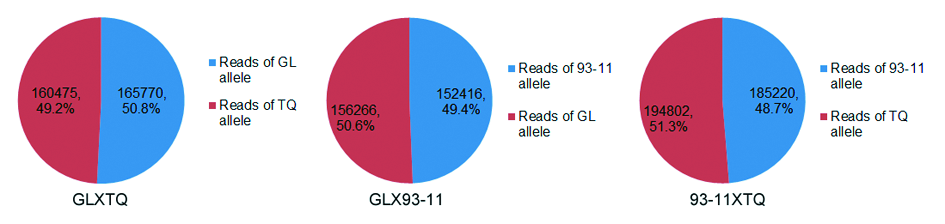

Supplement: Additional file 8: Figure S4 — The total read coverage of each allele from the two parents in the three F1 populations. [file 1471-2229-13-221-S8.tiff]

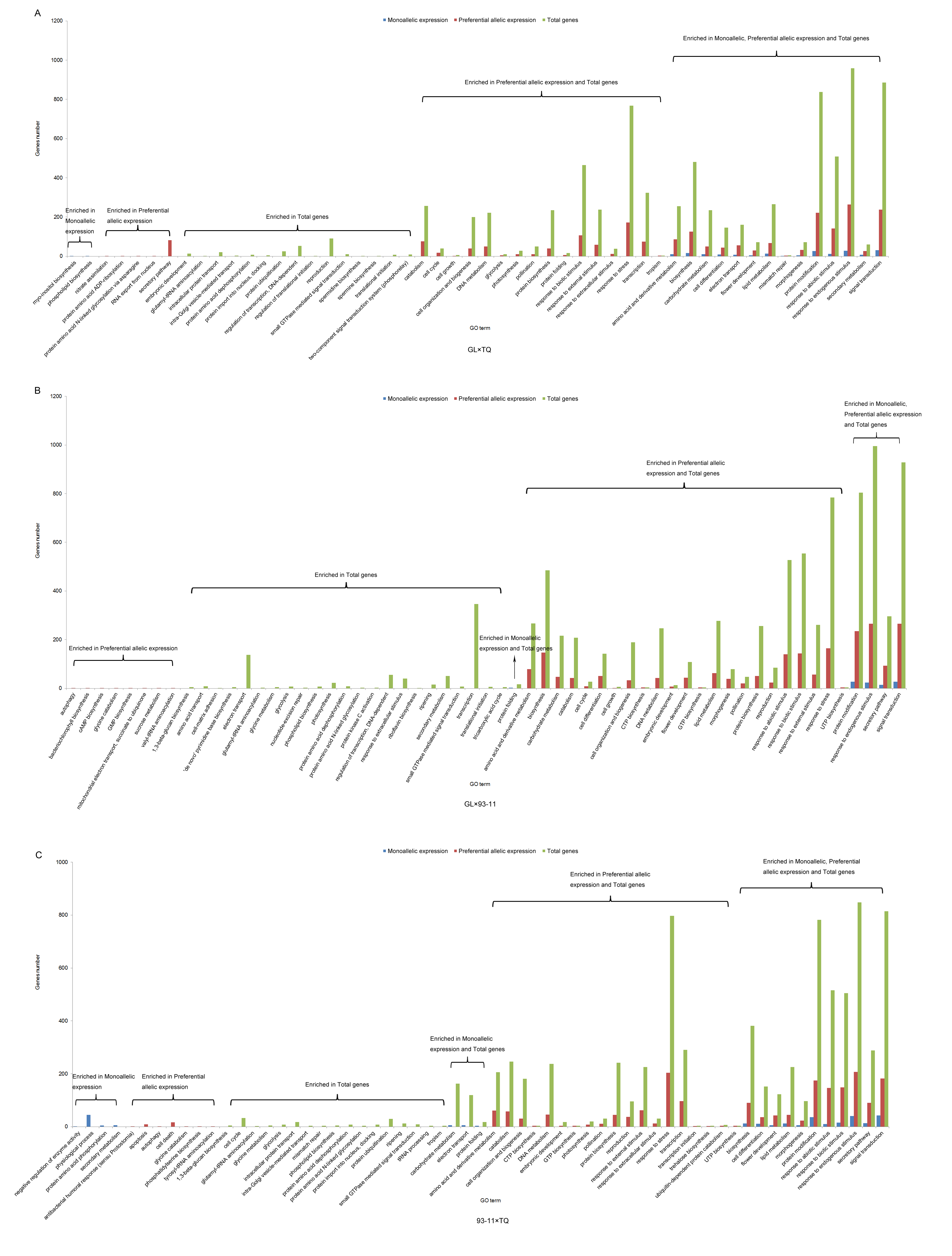

Supplement: Additional file 19: Figure S5 — Enriched biological functions in different groups of genes in GL × TQ (A), GL × 93-11 (B), and 93-11 × TQ (C). [file 1471-2229-13-221-S19.tiff]

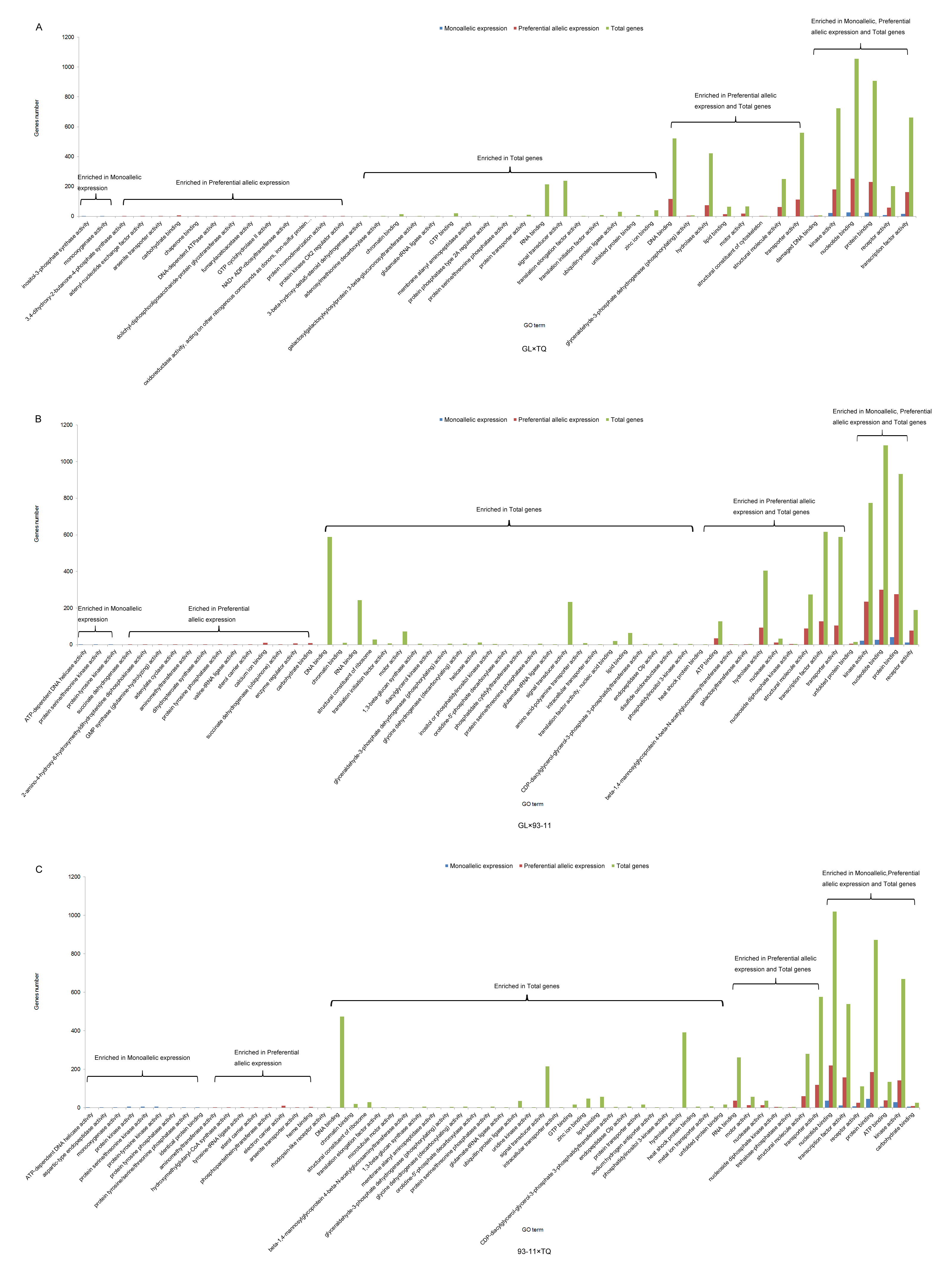

Supplement: Additional file 20: Figure S6 — Enriched molecular functions in different groups of genes in GL × TQ (A), GL × 93-11 (B), and 93-11 × TQ (C). [file 1471-2229-13-221-S20.tiff]
