# Supplementary material for: Global RNA sequencing reveals that genotype-dependent allele-specific expression contributes to differential expression in rice F1 hybrids
Source: BMC Plant Biol. 2013 Dec 21;13:221. doi: 10.1186/1471-2229-13-221 (PMC3878109; doi:10.1186/1471-2229-13-221)
Supplement: Additional file 3: Table S2 — Confirmed SNPs. [file 1471-2229-13-221-S3.docx]

Table S2. Confirmed SNPs

| SNP site | Chr | Nuclear type | Confirmed (Yes or No) | Material | Primer (5’ to 3’ F/R) |
| --- | --- | --- | --- | --- | --- |
| 8398273 | 1 | C | Yes | GL | CAACGGTATGTTTGTGCG  /CTGTTCGTCGTCGTCATC |
| 8677622 | 1 | T | Yes | GL | CACCGCTATTTTACTCCAAC -3'  / CCACATACCGCTCTAAGTTAC -3' |
| 12065051 | 1 | A | Yes | GL | CGTCCAAACTATCTCCCTAC -3'  / GAGTCCCAATCAAAATCGG -3' |
| 17836686 | 1 | C | Yes | GL | CGAATCTCAGTGCGATAATG -3'  / AAGCAGTCGGTCGGAGTC -3' |
| 18616857 | 1 | C | Yes | GL | GGTTGGTGGTGTTTGAATC -3'  / AGTGTGGTGTGGTAGGAGTAG -3' |
| 19323298 | 1 | T | Yes | GL | CCATTCTCCATCCATCCC -3'  / CAACTTCTTCCTTTCCTCTCTG -3' |
| 22758328 | 1 | C | Yes | GL | GGCAAAATAGGGAAACAGAG -3'  / GATGGACAGTGGCTGAGG -3' |
| 25272952 | 1 | G | Yes | GL | CTATGAAAGAACTTGGGCG -3'  / GATTTGCGTGTGAGTGGG -3' |
| 32216054 | 1 | A | Yes | GL | AACCCTTATTTCCGTGCC -3'  / TGCTCTGTCTGCCTAAACAC -3' |
| 33049018 | 1 | G | Yes | GL | AGGAAGAAACCGATGTAGG -3'  /GTTCAATAGATTCGTCTCGC -3' |
| 38365941 | 1 | T | Yes | GL | CAATCAGAAACAGTGGTTGG -3'  / CCTTACTAATCCCTCGGTTC -3' |
| 1140905 | 1 | C | Yes | GL | CAGATGAGTGAGCCTGCC  /CCTTGGATTATGCCTCCTG |
| 1874087 | 1 | C | Yes | GL | ATTCCGTGCCGCTTTATC  /CATTTTGTGCCACTTCGTTC |
| 2762405 | 1 | G | Yes | GL | GGGTTCATCAAGTGGTATCAG  /CAAAAGAAAGGCAGAGCG |
| 5268869 | 1 | G | Yes | GL | CGAGCAACATAAATGGAGC  /CGTGGGGAAAAAAGAAGTC |
| 5953925 | 1 | A | Yes | GL | CTAAACACAGCCGAACTTG  /GATGGGAGGAGAGAGAATG |
| 8167970 | 1 | G | Yes | GL | TCCATCTCTCACCTTCCAG  /GGGGTTTGGTATGATTTCTC |
| 10560193 | 1 | C | Yes | GL | AGCATCTGGTCACACAAGTC  /GGTCTATTTTGGAGGGAGTAG |
| 11997455 | 1 | T | Yes | GL | GCCGTAAAGCAAGAAACG  /TGGTAGCAAGCAGGGAAG |
| 13656198 | 1 | T | Yes | GL | CTTGTCTTTTTTCCTTCCCC  /TTCAAATGGTAGGCGTGC |
| 15863065 | 1 | G | Yes | GL | CCACCTCTCCCTCTCTTACC  /CAAAGTTCCGCATACATTCG |
| 17978264 | 1 | C | Yes | GL | AGAAGAAGGGCGATGTATG  /AGGCTCCAATCCAAAGTC |
| 18340456 | 1 | C | Yes | GL | GCAAAAGGAGGCTACATCTG  /GGCACTGTCGTGTGAACG |
| 3014302 | 2 | G | Yes | GL | ACCAACCACCACACCATC  /TCCACACAGCCTAACTCG |
| 10071639 | 2 | C | Yes | GL | GATTGGAACGATGAGCCC  /CGAGGAGGTTGTGGAACTC |
| 16983137 | 2 | T | Yes | GL | TTAGAGGAGTCGGAGCCC  /CATCTCGTCGCCTTCTTC |
| 17250491 | 2 | C | Yes | GL | ATGCGGAGCAGTTTAGGC  GGTTCGTGGCTTCTGGAG |
| 18712681 | 2 | A | Yes | GL | CCTCATCTACATTTTCGTCCAC  / GTCGGGTCCAACAGCAAG |
| 23692786 | 2 | C | Yes | GL | GAAAAATGCCCTCCAACAG  / GTCAGGTGCCAAGTTCCG |
| 24204313 | 2 | C | Yes | GL | TAGGGGTAATCTCGCTGG  /CCAAGTGTCTGAGTCGTGTG |
| 24771713 | 2 | G | Yes | GL | GCTATCCATCTCTATCTCGC  / AAATCCTGTCCGTTGGTG |
| 25635977 | 2 | G | Yes | GL | AGCCAAAAGCCTCAAGCG  / GTGCCGTTTCCATTGTCG |
| 25735624 | 2 | G | Yes | GL | GTTTTTCAGGTGCTGGGC  / GCTCTACTGGTGACGCTCC |
| 26555960 | 2 | G | Yes | GL | CAAAGAGATTGCCGCTTG  / GCTCAGAACCTGCTCATCC |
| 4525916 | 3 | G | Yes | GL | GGTTGGCTCTGTCGTGTAG  / CCTCCAGTGAACTCATTCC |
| 8331724 | 3 | C | Yes | GL | CCTGTTTCCTGGTGTGAAC  / CGAAGTATGACGATGGTGC |
| 8623614 | 3 | C | No | GL | TGCCGAGTTCCTTACGAC  / GCTCCCCTTCTCACTTCAC |
| 9767912 | 3 | G | Yes | GL | GCACCGCTGTTGTAGATG  / CTCATTGTCGTGGTCGTAG |
| 17836686 | 1 | T | Yes | 93-11 | CGAATCTCAGTGCGATAATG  / AAGCAGTCGGTCGGAGTC |
| 38365941 | 1 | C | Yes | 93-11 | CAATCAGAAACAGTGGTTGG  /CCTTACTAATCCCTCGGTTC |
| 15863065 | 1 | A | Yes | 93-11 | CCACCTCTCCCTCTCTTACC  /CAAAGTTCCGCATACATTCG |
| 8677622 | 1 | C | Yes | 93-11 | CACCGCTATTTTACTCCAAC  / CCACATACCGCTCTAAGTTAC |
| 18340456 | 1 | T | Yes | 93-11 | GCAAAAGGAGGCTACATCTG  /GGCACTGTCGTGTGAACG |
| 10071639 | 1 | T | Yes | 93-11 | GATTGGAACGATGAGCCC  /CGAGGAGGTTGTGGAACTC |
| 17250491 | 2 | T | Yes | 93-11 | ATGCGGAGCAGTTTAGGC  /GGTTCGTGGCTTCTGGAG |
| 18712681 | 2 | C | Yes | 93-11 | CCTCATCTACATTTTCGTCCAC  / GTCGGGTCCAACAGCAAG |
| 23692786  24204313 | 2  2 | T  T | Yes  Yes | 93-11  93-11 | GAAAAATGCCCTCCAACAG  / GTCAGGTGCCAAGTTCCG  TAGGGGTAATCTCGCTGG  /CCAAGTGTCTGAGTCGTGTG |
| 24771713 | 2 | A | Yes | 93-11 | GCTATCCATCTCTATCTCGC  /AAATCCTGTCCGTTGGTG |
| 25635977 | 2 | A | Yes | 93-11 | AGCCAAAAGCCTCAAGCG  / GTGCCGTTTCCATTGTCG |
| 8967027 | 3 | A | Yes | 93-11 | GGATGAACTCCCCTCTCC  / AGCAAGCCAGGAACACAG |
