# Supplementary material for: Global RNA sequencing reveals that genotype-dependent allele-specific expression contributes to differential expression in rice F1 hybrids
Source: BMC Plant Biol. 2013 Dec 21;13:221. doi: 10.1186/1471-2229-13-221 (PMC3878109; doi:10.1186/1471-2229-13-221)
Supplement: Additional file 5: Table S4 — Read coverage in the ASE analysis. [file 1471-2229-13-221-S5.docx]

Table S4. Read coverage in the ASE analysis

| Materials | GL×TQ | GL×93-11 | 93-11×TQ |
| --- | --- | --- | --- |
| SNPs for ASE analysis | 41,416 | 43,685 | 42,216 |
| Reads covered SNPs | 389,101 | 373,773 | 461,809 |
| Reads/SNP | 9.4 | 8.6 | 10.9 |
