# Supplementary material for: Global RNA sequencing reveals that genotype-dependent allele-specific expression contributes to differential expression in rice F1 hybrids
Source: BMC Plant Biol. 2013 Dec 21;13:221. doi: 10.1186/1471-2229-13-221 (PMC3878109; doi:10.1186/1471-2229-13-221)
Supplement: Additional file 9: Table S5 — List of all monoallelically expressed genes. [file 1471-2229-13-221-S9.docx]

Table S5. List of all monoallelically expressed genes

| Gene_id | Reads of P1 | P1 | Reads of P2 | | P2 | Annotation |
| --- | --- | --- | --- | --- | --- | --- |
| GL×TQ | | | | | | |
| LOC_Os01g09370 | 0 | GL | | 11 | TQ | ankyrin repeat domain-containing protein 28, putative, expressed |
| LOC_Os01g12100 | 0 | GL | | 11 | TQ | expressed protein |
| LOC_Os01g12304 | 0 | GL | | 12 | TQ | esterase, putative, expressed |
| LOC_Os01g49529 | 0 | GL | | 23 | TQ | OsWAK10d - OsWAK receptor-like cytoplasmic kinase OsWAK-RLCK, expressed |
| LOC_Os01g59300 | 0 | GL | | 52 | TQ | hypothetical protein |
| LOC_Os02g14520 | 0 | GL | | 34 | TQ | transposon protein, putative, CACTA, En/Spm sub-class, expressed |
| LOC_Os02g29210 | 0 | GL | | 157 | TQ | ankyrin, putative, expressed |
| LOC_Os02g38700 | 0 | GL | | 13 | TQ | transposon protein, putative, Ac/Ds sub-class |
| LOC_Os02g40130 | 0 | GL | | 241 | TQ | leucine-rich, putative, expressed |
| LOC_Os03g26350 | 0 | GL | | 25 | TQ | transposon protein, putative, CACTA, En/Spm sub-class |
| LOC_Os03g28270 | 0 | GL | | 11 | TQ | Leucine Rich Repeat family protein, expressed |
| LOC_Os04g12580 | 0 | GL | | 21 | TQ | receptor-like protein kinase, putative |
| LOC_Os04g19970 | 0 | GL | | 17 | TQ | retrotransposon, putative, centromere-specific |
| LOC_Os04g19980 | 0 | GL | | 41 | TQ | retrotransposon protein, putative, unclassified |
| LOC_Os04g23360 | 0 | GL | | 16 | TQ | retrotransposon protein, putative, unclassified, expressed |
| LOC_Os04g30200 | 0 | GL | | 13 | TQ | OsFBL14 - F-box domain and LRR containing protein, expressed |
| LOC_Os04g51009 | 0 | GL | | 17 | TQ | OsWAK52 - OsWAK short gene, expressed |
| LOC_Os04g52590 | 0 | GL | | 13 | TQ | protein kinase domain containing protein, expressed |
| LOC_Os05g13420 | 0 | GL | | 24 | TQ | large tegument protein, putative, expressed |
| LOC_Os05g23960 | 0 | GL | | 13 | TQ | pentatricopeptide, putative |
| LOC_Os05g41880 | 0 | GL | | 10 | TQ | mutS domain V family protein, expressed |
| LOC_Os05g43910 | 0 | GL | | 14 | TQ | cytochrome P450, putative, expressed |
| LOC_Os05g48790 | 0 | GL | | 126 | TQ | expressed protein |
| LOC_Os06g07810 | 0 | GL | | 11 | TQ | transposon protein, putative, unclassified |
| LOC_Os06g12470 | 0 | GL | | 99 | TQ | retrotransposon protein, putative, Ty3-gypsy subclass, expressed |
| LOC_Os06g13520 | 0 | GL | | 19 | TQ | SAM dependent carboxyl methyltransferase domain containing protein |
| LOC_Os06g38680 | 0 | GL | | 10 | TQ | expressed protein |
| LOC_Os06g39090 | 0 | GL | | 28 | TQ | transposon protein, putative, unclassified |
| LOC_Os06g46170 | 0 | GL | | 12 | TQ | retrotransposon protein, putative, unclassified |
| LOC_Os07g04420 | 0 | GL | | 10 | TQ | hypothetical protein |
| LOC_Os07g04430 | 0 | GL | | 19 | TQ | expressed protein |
| LOC_Os07g04480 | 0 | GL | | 13 | TQ | transposon protein, putative, unclassified, expressed |
| LOC_Os07g09460 | 0 | GL | | 186 | TQ | expressed protein |
| LOC_Os07g10940 | 0 | GL | | 13 | TQ | exo70 exocyst complex subunit family protein |
| LOC_Os07g19150 | 0 | GL | | 31 | TQ | cytochrome P450 71E1, putative |
| LOC_Os09g19380 | 0 | GL | | 13 | TQ | receptor-like protein kinase precursor, putative, expressed |
| LOC_Os09g19390 | 0 | GL | | 45 | TQ | senescence-induced receptor-like serine/threonine-protein kinase precursor, putative, expressed |
| LOC_Os09g20040 | 0 | GL | | 15 | TQ | resistance-like protein I2GA-SH194-2, putative, expressed |
| LOC_Os10g04750 | 0 | GL | | 12 | TQ | OsFBX362 - F-box domain containing protein |
| LOC_Os10g19120 | 0 | GL | | 10 | TQ | retrotransposon protein, putative, Ty3-gypsy subclass |
| LOC_Os10g20060 | 0 | GL | | 46 | TQ | hypothetical protein |
| LOC_Os10g22362 | 0 | GL | | 13 | TQ | hypothetical protein |
| LOC_Os10g24980 | 0 | GL | | 45 | TQ | retrotransposon protein, putative, unclassified |
| LOC_Os10g25180 | 0 | GL | | 13 | TQ | phosphoinositide phosphatase family protein, putative, expressed |
| LOC_Os10g29170 | 0 | GL | | 14 | TQ | transposon protein, putative, Ac/Ds sub-class |
| LOC_Os11g01990 | 0 | GL | | 11 | TQ | expressed protein |
| LOC_Os11g07140 | 0 | GL | | 38 | TQ | receptor kinase, putative, expressed |
| LOC_Os11g07980 | 0 | GL | | 95 | TQ | ion channel nompc, putative, expressed |
| LOC_Os11g08940 | 0 | GL | | 16 | TQ | RNA polymerases N 8 kDa subunit, putative |
| LOC_Os11g40009 | 0 | GL | | 18 | TQ | phospholipase, patatin family, putative, expressed |
| LOC_Os11g44960 | 0 | GL | | 10 | TQ | NBS-LRR disease resistance protein, putative, expressed |
| LOC_Os11g44990 | 0 | GL | | 195 | TQ | NB-ARC domain containing protein, expressed |
| LOC_Os11g47140 | 0 | GL | | 604 | TQ | OsWAK123 - OsWAK receptor-like protein kinase, expressed |
| LOC_Os12g02060 | 0 | GL | | 10 | TQ | peroxidase precursor, putative |
| LOC_Os12g02070 | 0 | GL | | 11 | TQ | expressed protein |
| LOC_Os12g20410 | 0 | GL | | 15 | TQ | matrix attachment region binding protein, putative, expressed |
| LOC_Os12g21570 | 0 | GL | | 32 | TQ | retrotransposon protein, putative, Ty3-gypsy subclass, expressed |
| LOC_Os12g21580 | 0 | GL | | 14 | TQ | retrotransposon protein, putative, Ty3-gypsy subclass |
| LOC_Os12g25830 | 0 | GL | | 11 | TQ | retrotransposon protein, putative, unclassified |
| LOC_Os12g28100 | 0 | GL | | 19 | TQ | NBS-LRR disease resistance protein, putative, expressed |
| LOC_Os12g32310 | 0 | GL | | 14 | TQ | hypothetical protein |
| LOC_Os12g36030 | 0 | GL | | 10 | TQ | expressed protein |
| LOC_Os12g39970 | 0 | GL | | 12 | TQ | expressed protein |
| LOC_Os01g10700 | 10 | GL | | 0 | TQ | D-mannose binding lectin family protein |
| LOC_Os01g35860 | 65 | GL | | 0 | TQ | transposon protein, putative, Mutator sub-class |
| LOC_Os01g35870 | 14 | GL | | 0 | TQ | hypothetical protein |
| LOC_Os02g05530 | 49 | GL | | 0 | TQ | hypothetical protein |
| LOC_Os02g18612 | 22 | GL | | 0 | TQ | expressed protein |
| LOC_Os02g31230 | 10 | GL | | 0 | TQ | expressed protein |
| LOC_Os02g38386 | 21 | GL | | 0 | TQ | NBS-LRR disease resistance protein, putative, expressed |
| LOC_Os02g38392 | 127 | GL | | 0 | TQ | NBS-LRR disease resistance protein, putative, expressed |
| LOC_Os02g57140 | 17 | GL | | 0 | TQ | expressed protein |
| LOC_Os02g57930 | 226 | GL | | 0 | TQ | retrotransposon protein, putative, Ty3-gypsy subclass |
| LOC_Os03g01420 | 111 | GL | | 0 | TQ | expressed protein |
| LOC_Os03g01480 | 15 | GL | | 0 | TQ | hypothetical protein |
| LOC_Os03g01520 | 10 | GL | | 0 | TQ | hypothetical protein |
| LOC_Os03g13690 | 10 | GL | | 0 | TQ | hypothetical protein |
| LOC_Os03g14920 | 10 | GL | | 0 | TQ | hypothetical protein |
| LOC_Os03g57560 | 18 | GL | | 0 | TQ | piwi domain containing protein, expressed |
| LOC_Os03g63450 | 24 | GL | | 0 | TQ | snRK1-interacting protein 1, putative, expressed |
| LOC_Os04g12990 | 14 | GL | | 0 | TQ | OsFBX122 - F-box domain containing protein |
| LOC_Os04g22960 | 38 | GL | | 0 | TQ | retrotransposon protein, putative, unclassified |
| LOC_Os04g22970 | 22 | GL | | 0 | TQ | retrotransposon protein, putative, unclassified |
| LOC_Os04g23040 | 87 | GL | | 0 | TQ | expressed protein |
| LOC_Os04g30030 | 111 | GL | | 0 | TQ | cysteine-rich receptor-like protein kinase 12 precursor, putative |
| LOC_Os04g30180 | 15 | GL | | 0 | TQ | F-box/LRR-repeat protein 14, putative, expressed |
| LOC_Os04g31260 | 11 | GL | | 0 | TQ | hypothetical protein |
| LOC_Os04g51250 | 13 | GL | | 0 | TQ | expressed protein |
| LOC_Os04g54110 | 12 | GL | | 0 | TQ | ARK3, putative |
| LOC_Os05g03320 | 49 | GL | | 0 | TQ | expressed protein |
| LOC_Os05g15940 | 11 | GL | | 0 | TQ | retrotransposon protein, putative, unclassified |
| LOC_Os05g46640 | 127 | GL | | 0 | TQ | hypothetical protein |
| LOC_Os05g46660 | 82 | GL | | 0 | TQ | transposon protein, putative, Mutator sub-class |
| LOC_Os05g46680 | 35 | GL | | 0 | TQ | retrotransposon protein, putative, unclassified |
| LOC_Os06g12140 | 14 | GL | | 0 | TQ | conserved hypothetical protein |
| LOC_Os06g15730 | 13 | GL | | 0 | TQ | expressed protein |
| LOC_Os06g40780 | 53 | GL | | 0 | TQ | MONOCULM 1, putative, expressed |
| LOC_Os07g01890 | 11 | GL | | 0 | TQ | expressed protein |
| LOC_Os07g01900 | 11 | GL | | 0 | TQ | expressed protein |
| LOC_Os07g07030 | 15 | GL | | 0 | TQ | expressed protein |
| LOC_Os07g16970 | 11 | GL | | 0 | TQ | rab GDP dissociation inhibitor alpha, putative, expressed |
| LOC_Os07g17689 | 35 | GL | | 0 | TQ | expressed protein |
| LOC_Os07g19210 | 10 | GL | | 0 | TQ | cytochrome P450, putative, expressed |
| LOC_Os07g47110 | 13 | GL | | 0 | TQ | OsFBT11 - F-box and tubby domain containing protein, expressed |
| LOC_Os08g01520 | 17 | GL | | 0 | TQ | cytochrome P450, putative |
| LOC_Os09g13510 | 14 | GL | | 0 | TQ | retrotransposon protein, putative, unclassified |
| LOC_Os09g15639 | 22 | GL | | 0 | TQ | expressed protein |
| LOC_Os09g15650 | 12 | GL | | 0 | TQ | hypothetical protein |
| LOC_Os09g18410 | 11 | GL | | 0 | TQ | retrotransposon protein, putative, Ty3-gypsy subclass |
| LOC_Os09g19229 | 106 | GL | | 0 | TQ | protein kinase domain containing protein, expressed |
| LOC_Os09g19350 | 62 | GL | | 0 | TQ | expressed protein |
| LOC_Os09g24170 | 340 | GL | | 0 | TQ | hypothetical protein |
| LOC_Os09g24180 | 103 | GL | | 0 | TQ | retrotransposon protein, putative, unclassified |
| LOC_Os09g24190 | 14 | GL | | 0 | TQ | hypothetical protein |
| LOC_Os10g03000 | 14 | GL | | 0 | TQ | retrotransposon protein, putative, unclassified |
| LOC_Os10g10300 | 16 | GL | | 0 | TQ | retrotransposon protein, putative, unclassified, expressed |
| LOC_Os10g15240 | 26 | GL | | 0 | TQ | retrotransposon protein, putative, Ty3-gypsy subclass |
| LOC_Os10g22450 | 10 | GL | | 0 | TQ | inositol-3-phosphate synthase, putative, expressed |
| LOC_Os10g24000 | 50 | GL | | 0 | TQ | hypothetical protein |
| LOC_Os10g24050 | 22 | GL | | 0 | TQ | ribosome inactivating protein, putative, expressed |
| LOC_Os10g24200 | 21 | GL | | 0 | TQ | retrotransposon protein, putative, Ty3-gypsy subclass |
| LOC_Os11g10850 | 11 | GL | | 0 | TQ | dirigent, putative |
| LOC_Os11g12350 | 23 | GL | | 0 | TQ | disease resistance protein, putative, expressed |
| LOC_Os11g13680 | 63 | GL | | 0 | TQ | expressed protein |
| LOC_Os11g18790 | 18 | GL | | 0 | TQ | retrotransposon protein, putative, unclassified |
| LOC_Os11g18800 | 12 | GL | | 0 | TQ | retrotransposon protein, putative, unclassified |
| LOC_Os11g18810 | 56 | GL | | 0 | TQ | retrotransposon protein, putative, Ty3-gypsy subclass |
| LOC_Os11g18820 | 31 | GL | | 0 | TQ | retrotransposon protein, putative, Ty3-gypsy subclass |
| LOC_Os11g25830 | 10 | GL | | 0 | TQ | retrotransposon protein, putative, unclassified |
| LOC_Os11g27799 | 13 | GL | | 0 | TQ | expressed protein |
| LOC_Os11g29970 | 171 | GL | | 0 | TQ | NB-ARC domain containing protein, expressed |
| LOC_Os11g35274 | 29 | GL | | 0 | TQ | protein kinase domain containing protein, expressed |
| LOC_Os11g39190 | 17 | GL | | 0 | TQ | NB-ARC domain containing protein, putative, expressed |
| LOC_Os11g41210 | 28 | GL | | 0 | TQ | disease resistance protein RPM1, putative, expressed |
| LOC_Os11g45190 | 12 | GL | | 0 | TQ | NB-ARC domain containing protein, expressed |
| LOC_Os11g45220 | 22 | GL | | 0 | TQ | IWS1 homolog A, putative, expressed |
| LOC_Os11g45990 | 15 | GL | | 0 | TQ | von Willebrand factor type A domain containing protein, putative, expressed |
| LOC_Os12g07370 | 44 | GL | | 0 | TQ | csAtPR5, putative, expressed |
| LOC_Os12g07380 | 10 | GL | | 0 | TQ | expressed protein |
| LOC_Os12g24800 | 677 | GL | | 0 | TQ | 9-cis-epoxycarotenoid dioxygenase 1, chloroplast precursor, putative, expressed |
| LOC_Os12g28110 | 10 | GL | | 0 | TQ | conserved hypothetical protein |
| LOC_Os12g29150 | 33 | GL | | 0 | TQ | retrotransposon protein, putative, Ty3-gypsy subclass |
| LOC_Os12g30760 | 25 | GL | | 0 | TQ | disease resistance protein, putative |
| GL×93-11 | | | | | | |
| LOC_Os01g14790 | 0 | 93-11 | | 12 | GL | expressed protein |
| LOC_Os01g15270 | 0 | 93-11 | | 14 | GL | expressed protein |
| LOC_Os01g15510 | 0 | 93-11 | | 11 | GL | hypothetical protein |
| LOC_Os01g19140 | 0 | 93-11 | | 15 | GL | O-sialoglycoprotein endopeptidase, putative, expressed |
| LOC_Os01g25450 | 0 | 93-11 | | 11 | GL | AIG1 family protein, expressed |
| LOC_Os01g26280 | 0 | 93-11 | | 26 | GL | OsWAK8 - OsWAK receptor-like protein kinase, expressed |
| LOC_Os01g32439 | 0 | 93-11 | | 28 | GL | expressed protein |
| LOC_Os01g55090 | 0 | 93-11 | | 19 | GL | transposon protein, putative, Mutator sub-class, expressed |
| LOC_Os01g56850 | 0 | 93-11 | | 12 | GL | expressed protein |
| LOC_Os02g05530 | 0 | 93-11 | | 43 | GL | hypothetical protein |
| LOC_Os02g31230 | 0 | 93-11 | | 22 | GL | expressed protein |
| LOC_Os03g01420 | 0 | 93-11 | | 155 | GL | expressed protein |
| LOC_Os03g01480 | 0 | 93-11 | | 35 | GL | hypothetical protein |
| LOC_Os03g04720 | 0 | 93-11 | | 10 | GL | retrotransposon protein, putative, unclassified |
| LOC_Os03g50290 | 0 | 93-11 | | 15 | GL | 14-3-3 protein, putative, expressed |
| LOC_Os04g22950 | 0 | 93-11 | | 50 | GL | hypothetical protein |
| LOC_Os04g22960 | 0 | 93-11 | | 25 | GL | retrotransposon protein, putative, unclassified |
| LOC_Os04g22970 | 0 | 93-11 | | 44 | GL | retrotransposon protein, putative, unclassified |
| LOC_Os04g23040 | 0 | 93-11 | | 76 | GL | expressed protein |
| LOC_Os04g23140 | 0 | 93-11 | | 18 | GL | expressed protein |
| LOC_Os04g23799 | 0 | 93-11 | | 37 | GL | retrotransposon protein, putative, Ty3-gypsy subclass |
| LOC_Os04g24510 | 0 | 93-11 | | 10 | GL | OsWAK36 - OsWAK receptor-like protein kinase, expressed |
| LOC_Os04g24620 | 0 | 93-11 | | 10 | GL | retrotransposon protein, putative, Ty3-gypsy subclass |
| LOC_Os04g30180 | 0 | 93-11 | | 27 | GL | F-box/LRR-repeat protein 14, putative, expressed |
| LOC_Os04g30240 | 0 | 93-11 | | 21 | GL | OsWAK60 - OsWAK receptor-like protein kinase, expressed |
| LOC_Os04g36580 | 0 | 93-11 | | 80 | GL | retrotransposon protein, putative, unclassified |
| LOC_Os04g38060 | 0 | 93-11 | | 24 | GL | retrotransposon protein, putative, unclassified |
| LOC_Os05g08900 | 0 | 93-11 | | 19 | GL | expressed protein |
| LOC_Os06g14350 | 0 | 93-11 | | 24 | GL | caleosin related protein, putative, expressed |
| LOC_Os06g15730 | 0 | 93-11 | | 31 | GL | expressed protein |
| LOC_Os06g28300 | 0 | 93-11 | | 29 | GL | zinc knuckle family protein, expressed |
| LOC_Os06g42650 | 0 | 93-11 | | 10 | GL | hypothetical protein |
| LOC_Os07g01900 | 0 | 93-11 | | 45 | GL | expressed protein |
| LOC_Os07g45560 | 0 | 93-11 | | 59 | GL | conserved hypothetical protein |
| LOC_Os08g14880 | 0 | 93-11 | | 195 | GL | transposon protein, putative, unclassified, expressed |
| LOC_Os08g23020 | 0 | 93-11 | | 34 | GL | retrotransposon protein, putative, unclassified |
| LOC_Os08g23200 | 0 | 93-11 | | 17 | GL | transposon protein, putative, Ac/Ds sub-class |
| LOC_Os08g41630 | 0 | 93-11 | | 10 | GL | ubiquitin carboxyl-terminal hydrolase family protein, expressed |
| LOC_Os09g01980 | 0 | 93-11 | | 11 | GL | retrotransposon protein, putative, unclassified |
| LOC_Os10g03000 | 0 | 93-11 | | 18 | GL | retrotransposon protein, putative, unclassified |
| LOC_Os10g03669 | 0 | 93-11 | | 11 | GL | expressed protein |
| LOC_Os10g04342 | 0 | 93-11 | | 39 | GL | stripe rust resistance protein Yr10, putative, expressed |
| LOC_Os10g04720 | 0 | 93-11 | | 63 | GL | TKL_IRAK_DUF26-la.5 - DUF26 kinases have homology to DUF26 containing loci, expressed |
| LOC_Os10g04730 | 0 | 93-11 | | 508 | GL | TKL_IRAK_DUF26-la.6 - DUF26 kinases have homology to DUF26 containing loci, expressed |
| LOC_Os10g10300 | 0 | 93-11 | | 34 | GL | retrotransposon protein, putative, unclassified, expressed |
| LOC_Os10g10750 | 0 | 93-11 | | 24 | GL | expressed protein |
| LOC_Os10g15240 | 0 | 93-11 | | 23 | GL | retrotransposon protein, putative, Ty3-gypsy subclass |
| LOC_Os10g24000 | 0 | 93-11 | | 37 | GL | hypothetical protein |
| LOC_Os10g24050 | 0 | 93-11 | | 23 | GL | ribosome inactivating protein, putative, expressed |
| LOC_Os10g24200 | 0 | 93-11 | | 12 | GL | retrotransposon protein, putative, Ty3-gypsy subclass |
| LOC_Os11g18780 | 0 | 93-11 | | 11 | GL | retrotransposon protein, putative, unclassified |
| LOC_Os11g18800 | 0 | 93-11 | | 14 | GL | retrotransposon protein, putative, unclassified |
| LOC_Os11g18810 | 0 | 93-11 | | 79 | GL | retrotransposon protein, putative, Ty3-gypsy subclass |
| LOC_Os11g18820 | 0 | 93-11 | | 33 | GL | retrotransposon protein, putative, Ty3-gypsy subclass |
| LOC_Os11g27799 | 0 | 93-11 | | 12 | GL | expressed protein |
| LOC_Os11g36560 | 0 | 93-11 | | 15 | GL | zinc finger C3HC4 type family protein, putative |
| LOC_Os11g39310 | 0 | 93-11 | | 66 | GL | NB-ARC domain containing protein, expressed |
| LOC_Os11g40160 | 0 | 93-11 | | 101 | GL | expressed protein |
| LOC_Os11g41210 | 0 | 93-11 | | 25 | GL | disease resistance protein RPM1, putative, expressed |
| LOC_Os11g41540 | 0 | 93-11 | | 94 | GL | disease resistance RPP8-like protein 3, putative, expressed |
| LOC_Os11g45190 | 0 | 93-11 | | 23 | GL | NB-ARC domain containing protein, expressed |
| LOC_Os12g10770 | 0 | 93-11 | | 17 | GL | retrotransposon protein, putative, unclassified |
| LOC_Os12g13270 | 0 | 93-11 | | 163 | GL | expressed protein |
| LOC_Os12g13295 | 0 | 93-11 | | 24 | GL | expressed protein |
| LOC_Os12g22010 | 0 | 93-11 | | 10 | GL | hypothetical protein |
| LOC_Os12g22060 | 0 | 93-11 | | 13 | GL | expressed protein |
| LOC_Os12g22284 | 0 | 93-11 | | 71 | GL | white-brown complex homolog protein 11, putative, expressed |
| LOC_Os12g24800 | 0 | 93-11 | | 169 | GL | 9-cis-epoxycarotenoid dioxygenase 1, chloroplast precursor, putative, expressed |
| LOC_Os12g29150 | 0 | 93-11 | | 11 | GL | retrotransposon protein, putative, Ty3-gypsy subclass |
| LOC_Os12g29160 | 0 | 93-11 | | 14 | GL | LTPL105 - Protease inhibitor/seed storage/LTP family protein precursor, putative |
| LOC_Os12g29690 | 0 | 93-11 | | 12 | GL | NBS-LRR disease resistance protein, putative |
| LOC_Os01g02440 | 11 | 93-11 | | 0 | GL | Ser/Thr receptor-like kinase, putative, expressed |
| LOC_Os01g09320 | 10 | 93-11 | | 0 | GL | NADP-dependent malic enzyme, chloroplast precursor, putative, expressed |
| LOC_Os01g20880 | 27 | 93-11 | | 0 | GL | OsWAK3 - OsWAK receptor-like cytoplasmic kinase OsWAK-RLCK |
| LOC_Os01g26210 | 52 | 93-11 | | 0 | GL | OsWAK6 - OsWAK receptor-like protein kinase, expressed |
| LOC_Os01g31830 | 13 | 93-11 | | 0 | GL | hypothetical protein |
| LOC_Os01g33960 | 15 | 93-11 | | 0 | GL | transposon protein, putative, CACTA, En/Spm sub-class |
| LOC_Os01g42330 | 26 | 93-11 | | 0 | GL | retrotransposon protein, putative, unclassified |
| LOC_Os03g32330 | 20 | 93-11 | | 0 | GL | expressed protein |
| LOC_Os03g35970 | 301 | 93-11 | | 0 | GL | retrotransposon protein, putative, unclassified |
| LOC_Os04g19970 | 16 | 93-11 | | 0 | GL | retrotransposon, putative, centromere-specific |
| LOC_Os04g19980 | 51 | 93-11 | | 0 | GL | retrotransposon protein, putative, unclassified |
| LOC_Os04g24274 | 20 | 93-11 | | 0 | GL | hypothetical protein |
| LOC_Os04g29680 | 16 | 93-11 | | 0 | GL | OsWAK38 - OsWAK receptor-like protein kinase, expressed |
| LOC_Os04g38490 | 13 | 93-11 | | 0 | GL | hypothetical protein |
| LOC_Os04g41000 | 22 | 93-11 | | 0 | GL | expressed protein |
| LOC_Os04g52590 | 13 | 93-11 | | 0 | GL | protein kinase domain containing protein, expressed |
| LOC_Os04g53496 | 19 | 93-11 | | 0 | GL | NBS-LRR disease resistance protein, putative |
| LOC_Os06g38680 | 23 | 93-11 | | 0 | GL | expressed protein |
| LOC_Os06g39090 | 37 | 93-11 | | 0 | GL | transposon protein, putative, unclassified |
| LOC_Os07g04390 | 49 | 93-11 | | 0 | GL | hypothetical protein |
| LOC_Os07g04430 | 48 | 93-11 | | 0 | GL | expressed protein |
| LOC_Os07g04480 | 20 | 93-11 | | 0 | GL | transposon protein, putative, unclassified, expressed |
| LOC_Os07g04490 | 51 | 93-11 | | 0 | GL | hypothetical protein |
| LOC_Os07g09460 | 149 | 93-11 | | 0 | GL | expressed protein |
| LOC_Os07g10940 | 67 | 93-11 | | 0 | GL | exo70 exocyst complex subunit family protein |
| LOC_Os07g30980 | 14 | 93-11 | | 0 | GL | uvrD/REP helicase family protein, putative, expressed |
| LOC_Os08g07080 | 43 | 93-11 | | 0 | GL | terpene synthase, putative, expressed |
| LOC_Os08g10250 | 51 | 93-11 | | 0 | GL | SHR5-receptor-like kinase, putative, expressed |
| LOC_Os08g14860 | 257 | 93-11 | | 0 | GL | cytochrome b-c1 complex subunit 7, putative, expressed |
| LOC_Os08g18079 | 14 | 93-11 | | 0 | GL | expressed protein |
| LOC_Os08g21530 | 30 | 93-11 | | 0 | GL | hypothetical protein |
| LOC_Os08g21879 | 32 | 93-11 | | 0 | GL | expressed protein |
| LOC_Os08g23090 | 12 | 93-11 | | 0 | GL | retrotransposon protein, putative, unclassified |
| LOC_Os08g24850 | 44 | 93-11 | | 0 | GL | retrotransposon, putative, centromere-specific |
| LOC_Os08g27580 | 12 | 93-11 | | 0 | GL | expressed protein |
| LOC_Os08g30110 | 15 | 93-11 | | 0 | GL | transposon protein, putative, CACTA, En/Spm sub-class |
| LOC_Os10g04750 | 10 | 93-11 | | 0 | GL | OsFBX362 - F-box domain containing protein |
| LOC_Os10g08830 | 27 | 93-11 | | 0 | GL | conserved hypothetical protein |
| LOC_Os10g19990 | 69 | 93-11 | | 0 | GL | hypothetical protein |
| LOC_Os10g20060 | 112 | 93-11 | | 0 | GL | hypothetical protein |
| LOC_Os10g20080 | 39 | 93-11 | | 0 | GL | hypothetical protein |
| LOC_Os10g24980 | 59 | 93-11 | | 0 | GL | retrotransposon protein, putative, unclassified |
| LOC_Os10g29170 | 19 | 93-11 | | 0 | GL | transposon protein, putative, Ac/Ds sub-class |
| LOC_Os11g07140 | 210 | 93-11 | | 0 | GL | receptor kinase, putative, expressed |
| LOC_Os11g07150 | 14 | 93-11 | | 0 | GL | retrotransposon protein, putative, unclassified |
| LOC_Os11g15450 | 10 | 93-11 | | 0 | GL | retrotransposon protein, putative, unclassified |
| LOC_Os11g15670 | 34 | 93-11 | | 0 | GL | NBS-LRR disease resistance protein, putative, expressed |
| LOC_Os11g29090 | 13 | 93-11 | | 0 | GL | NB-ARC/LRR disease resistance protein, putative |
| LOC_Os11g29110 | 24 | 93-11 | | 0 | GL | Leucine Rich Repeat family protein, expressed |
| LOC_Os11g40009 | 11 | 93-11 | | 0 | GL | phospholipase, patatin family, putative, expressed |
| LOC_Os11g40249 | 73 | 93-11 | | 0 | GL | expressed protein |
| LOC_Os11g40400 | 15 | 93-11 | | 0 | GL | protein kinase, putative, expressed |
| LOC_Os11g44990 | 133 | 93-11 | | 0 | GL | NB-ARC domain containing protein, expressed |
| LOC_Os11g47452 | 14 | 93-11 | | 0 | GL | retrotransposon protein, putative, unclassified |
| LOC_Os12g18300 | 43 | 93-11 | | 0 | GL | retrotransposon protein, putative, unclassified |
| LOC_Os12g18320 | 10 | 93-11 | | 0 | GL | retrotransposon protein, putative, Ty3-gypsy subclass |
| LOC_Os12g20410 | 13 | 93-11 | | 0 | GL | matrix attachment region binding protein, putative, expressed |
| LOC_Os12g24100 | 10 | 93-11 | | 0 | GL | retrotransposon protein, putative, unclassified |
| 93-11×TQ | | | | | | |
| LOC_Os01g15270 | 0 | 93-11 | | 18 | TQ | expressed protein |
| LOC_Os01g15910 | 0 | 93-11 | | 12 | TQ | UTP--glucose-1-phosphate uridylyltransferase, putative |
| LOC_Os01g19140 | 0 | 93-11 | | 29 | TQ | O-sialoglycoprotein endopeptidase, putative, expressed |
| LOC_Os01g25450 | 0 | 93-11 | | 27 | TQ | AIG1 family protein, expressed |
| LOC_Os01g29469 | 0 | 93-11 | | 10 | TQ | dual specificity protein phosphatase, putative, expressed |
| LOC_Os01g32439 | 0 | 93-11 | | 27 | TQ | expressed protein |
| LOC_Os01g55090 | 0 | 93-11 | | 39 | TQ | transposon protein, putative, Mutator sub-class, expressed |
| LOC_Os01g59300 | 0 | 93-11 | | 11 | TQ | hypothetical protein |
| LOC_Os01g59570 | 0 | 93-11 | | 11 | TQ | senescence-induced receptor-like serine/threonine-protein kinase precursor, putative, expressed |
| LOC_Os02g14520 | 0 | 93-11 | | 39 | TQ | transposon protein, putative, CACTA, En/Spm sub-class, expressed |
| LOC_Os03g26080 | 0 | 93-11 | | 22 | TQ | nucleoside-triphosphatase, putative, expressed |
| LOC_Os03g26350 | 0 | 93-11 | | 35 | TQ | transposon protein, putative, CACTA, En/Spm sub-class |
| LOC_Os04g29424 | 0 | 93-11 | | 18 | TQ | retrotransposon protein, putative, LINE subclass |
| LOC_Os04g30240 | 0 | 93-11 | | 24 | TQ | OsWAK60 - OsWAK receptor-like protein kinase, expressed |
| LOC_Os04g30250 | 0 | 93-11 | | 10 | TQ | wall-associated receptor kinase-like 5 precursor, putative, expressed |
| LOC_Os04g37990 | 0 | 93-11 | | 11 | TQ | transporter family protein, putative, expressed |
| LOC_Os04g38060 | 0 | 93-11 | | 26 | TQ | retrotransposon protein, putative, unclassified |
| LOC_Os05g11290 | 0 | 93-11 | | 10 | TQ | retrotransposon protein, putative, Ty1-copia subclass |
| LOC_Os05g13410 | 0 | 93-11 | | 45 | TQ | retrotransposon protein, putative, unclassified |
| LOC_Os05g13420 | 0 | 93-11 | | 56 | TQ | large tegument protein, putative, expressed |
| LOC_Os05g15220 | 0 | 93-11 | | 102 | TQ | retrotransposon protein, putative, unclassified, expressed |
| LOC_Os05g15330 | 0 | 93-11 | | 22 | TQ | hypothetical protein |
| LOC_Os05g15340 | 0 | 93-11 | | 12 | TQ | retrotransposon protein, putative, unclassified |
| LOC_Os05g43910 | 0 | 93-11 | | 23 | TQ | cytochrome P450, putative, expressed |
| LOC_Os05g46110 | 0 | 93-11 | | 18 | TQ | retrotransposon protein, putative, unclassified |
| LOC_Os06g06960 | 0 | 93-11 | | 22 | TQ | S-locus-like receptor protein kinase, putative, expressed |
| LOC_Os06g07810 | 0 | 93-11 | | 11 | TQ | transposon protein, putative, unclassified |
| LOC_Os06g12470 | 0 | 93-11 | | 103 | TQ | retrotransposon protein, putative, Ty3-gypsy subclass, expressed |
| LOC_Os06g13520 | 0 | 93-11 | | 36 | TQ | SAM dependent carboxyl methyltransferase domain containing protein |
| LOC_Os06g34460 | 0 | 93-11 | | 16 | TQ | hypothetical protein |
| LOC_Os06g35600 | 0 | 93-11 | | 12 | TQ | retrotransposon, putative, centromere-specific |
| LOC_Os07g17230 | 0 | 93-11 | | 16 | TQ | disease resistance protein, putative, expressed |
| LOC_Os07g19150 | 0 | 93-11 | | 32 | TQ | cytochrome P450 71E1, putative |
| LOC_Os07g31250 | 0 | 93-11 | | 13 | TQ | OsWAK69 - OsWAK receptor-like cytoplasmic kinase OsWAK-RLCK, expressed |
| LOC_Os07g33690 | 0 | 93-11 | | 43 | TQ | NBS-LRR type disease resistance protein Hom-F, putative, expressed |
| LOC_Os07g45560 | 0 | 93-11 | | 57 | TQ | conserved hypothetical protein |
| LOC_Os08g05440 | 0 | 93-11 | | 14 | TQ | NB-ARC domain containing protein |
| LOC_Os08g10760 | 0 | 93-11 | | 10 | TQ | hypothetical protein |
| LOC_Os08g14880 | 0 | 93-11 | | 165 | TQ | transposon protein, putative, unclassified, expressed |
| LOC_Os08g14990 | 0 | 93-11 | | 15 | TQ | receptor-like protein kinase 2 precursor, putative, expressed |
| LOC_Os08g29400 | 0 | 93-11 | | 16 | TQ | OsFBX290 - F-box domain containing protein, expressed |
| LOC_Os10g01380 | 0 | 93-11 | | 31 | TQ | CW7, putative, expressed |
| LOC_Os10g03900 | 0 | 93-11 | | 28 | TQ | expressed protein |
| LOC_Os10g04060 | 0 | 93-11 | | 13 | TQ | powdery mildew resistance protein PM3b, putative |
| LOC_Os10g04342 | 0 | 93-11 | | 50 | TQ | stripe rust resistance protein Yr10, putative, expressed |
| LOC_Os10g04730 | 0 | 93-11 | | 36 | TQ | TKL_IRAK_DUF26-la.6 - DUF26 kinases have homology to DUF26 containing loci, expressed |
| LOC_Os10g25180 | 0 | 93-11 | | 10 | TQ | phosphoinositide phosphatase family protein, putative, expressed |
| LOC_Os11g07250 | 0 | 93-11 | | 10 | TQ | receptor kinase, putative, expressed |
| LOC_Os11g07720 | 0 | 93-11 | | 15 | TQ | retrotransposon protein, putative, unclassified |
| LOC_Os11g07980 | 0 | 93-11 | | 113 | TQ | ion channel nompc, putative, expressed |
| LOC_Os11g08830 | 0 | 93-11 | | 81 | TQ | kelch domain containing protein, putative |
| LOC_Os11g08940 | 0 | 93-11 | | 25 | TQ | RNA polymerases N 8 kDa subunit, putative |
| LOC_Os11g29490 | 0 | 93-11 | | 16 | TQ | plasma membrane ATPase, putative, expressed |
| LOC_Os11g36970 | 0 | 93-11 | | 13 | TQ | zinc finger C3HC4 type family protein, putative |
| LOC_Os12g10770 | 0 | 93-11 | | 45 | TQ | retrotransposon protein, putative, unclassified |
| LOC_Os12g12514 | 0 | 93-11 | | 45 | TQ | NADP-dependent oxidoreductase, putative |
| LOC_Os12g16520 | 0 | 93-11 | | 19 | TQ | wall-associated receptor kinase 3 precursor, putative, expressed |
| LOC_Os12g17470 | 0 | 93-11 | | 15 | TQ | retrotransposon protein, putative, unclassified |
| LOC_Os12g21570 | 0 | 93-11 | | 12 | TQ | retrotransposon protein, putative, Ty3-gypsy subclass, expressed |
| LOC_Os12g22010 | 0 | 93-11 | | 12 | TQ | hypothetical protein |
| LOC_Os12g22080 | 0 | 93-11 | | 10 | TQ | retrotransposon protein, putative, Ty3-gypsy subclass |
| LOC_Os12g25830 | 0 | 93-11 | | 14 | TQ | retrotransposon protein, putative, unclassified |
| LOC_Os12g28100 | 0 | 93-11 | | 14 | TQ | NBS-LRR disease resistance protein, putative, expressed |
| LOC_Os12g29160 | 0 | 93-11 | | 28 | TQ | LTPL105 - Protease inhibitor/seed storage/LTP family protein precursor, putative |
| LOC_Os12g29690 | 0 | 93-11 | | 93 | TQ | NBS-LRR disease resistance protein, putative |
| LOC_Os12g32710 | 0 | 93-11 | | 10 | TQ | NB-ARC domain containing protein, expressed |
| LOC_Os12g35465 | 0 | 93-11 | | 369 | TQ | expressed protein |
| LOC_Os12g36030 | 0 | 93-11 | | 42 | TQ | expressed protein |
| LOC_Os12g36400 | 0 | 93-11 | | 167 | TQ | transposon protein, putative, Pong sub-class |
| LOC_Os12g37260 | 0 | 93-11 | | 66 | TQ | lipoxygenase 2.1, chloroplast precursor, putative, expressed |
| LOC_Os01g20880 | 44 | 93-11 | | 0 | TQ | OsWAK3 - OsWAK receptor-like cytoplasmic kinase OsWAK-RLCK |
| LOC_Os01g21130 | 36 | 93-11 | | 0 | TQ | expressed protein |
| LOC_Os01g24200 | 14 | 93-11 | | 0 | TQ | hypothetical protein |
| LOC_Os01g26210 | 92 | 93-11 | | 0 | TQ | OsWAK6 - OsWAK receptor-like protein kinase, expressed |
| LOC_Os01g31830 | 15 | 93-11 | | 0 | TQ | hypothetical protein |
| LOC_Os01g33684 | 46 | 93-11 | | 0 | TQ | disease resistance RPP13-like protein 1, putative, expressed |
| LOC_Os01g40980 | 11 | 93-11 | | 0 | TQ | helicase, putative, expressed |
| LOC_Os01g42330 | 27 | 93-11 | | 0 | TQ | retrotransposon protein, putative, unclassified |
| LOC_Os02g10340 | 11 | 93-11 | | 0 | TQ | retrotransposon protein, putative, unclassified |
| LOC_Os02g12450 | 21 | 93-11 | | 0 | TQ | receptor-like protein kinase 2 precursor, putative |
| LOC_Os02g16995 | 12 | 93-11 | | 0 | TQ | expressed protein |
| LOC_Os02g18780 | 15 | 93-11 | | 0 | TQ | hypothetical protein |
| LOC_Os03g32330 | 29 | 93-11 | | 0 | TQ | expressed protein |
| LOC_Os03g35970 | 526 | 93-11 | | 0 | TQ | retrotransposon protein, putative, unclassified |
| LOC_Os03g37240 | 18 | 93-11 | | 0 | TQ | retrotransposon protein, putative, unclassified |
| LOC_Os04g29680 | 22 | 93-11 | | 0 | TQ | OsWAK38 - OsWAK receptor-like protein kinase, expressed |
| LOC_Os05g03320 | 113 | 93-11 | | 0 | TQ | expressed protein |
| LOC_Os05g03390 | 29 | 93-11 | | 0 | TQ | expressed protein |
| LOC_Os05g20460 | 17 | 93-11 | | 0 | TQ | retrotransposon protein, putative, unclassified |
| LOC_Os05g45580 | 15 | 93-11 | | 0 | TQ | expressed protein |
| LOC_Os05g45590 | 12 | 93-11 | | 0 | TQ | hexokinase, putative, expressed |
| LOC_Os06g12140 | 14 | 93-11 | | 0 | TQ | conserved hypothetical protein |
| LOC_Os06g14040 | 14 | 93-11 | | 0 | TQ | ThiF family domain containing protein, putative, expressed |
| LOC_Os06g40770 | 19 | 93-11 | | 0 | TQ | expressed protein |
| LOC_Os06g40780 | 39 | 93-11 | | 0 | TQ | MONOCULM 1, putative, expressed |
| LOC_Os06g48650 | 16 | 93-11 | | 0 | TQ | OsSub52 - Putative Subtilisin homologue, expressed |
| LOC_Os07g07030 | 29 | 93-11 | | 0 | TQ | expressed protein |
| LOC_Os07g15930 | 13 | 93-11 | | 0 | TQ | legume lectins beta domain containing protein, expressed |
| LOC_Os07g17689 | 44 | 93-11 | | 0 | TQ | expressed protein |
| LOC_Os07g23270 | 18 | 93-11 | | 0 | TQ | transposon protein, putative, CACTA, En/Spm sub-class |
| LOC_Os07g23900 | 20 | 93-11 | | 0 | TQ | OsFBX234 - F-box domain containing protein, expressed |
| LOC_Os07g28644 | 10 | 93-11 | | 0 | TQ | expressed protein |
| LOC_Os07g30980 | 51 | 93-11 | | 0 | TQ | uvrD/REP helicase family protein, putative, expressed |
| LOC_Os07g45570 | 33 | 93-11 | | 0 | TQ | expressed protein |
| LOC_Os08g01520 | 12 | 93-11 | | 0 | TQ | cytochrome P450, putative |
| LOC_Os08g07080 | 19 | 93-11 | | 0 | TQ | terpene synthase, putative, expressed |
| LOC_Os08g10250 | 85 | 93-11 | | 0 | TQ | SHR5-receptor-like kinase, putative, expressed |
| LOC_Os08g15830 | 11 | 93-11 | | 0 | TQ | retrotransposon protein, putative, unclassified |
| LOC_Os08g17140 | 12 | 93-11 | | 0 | TQ | retrotransposon protein, putative, unclassified, expressed |
| LOC_Os08g18079 | 36 | 93-11 | | 0 | TQ | expressed protein |
| LOC_Os08g21530 | 42 | 93-11 | | 0 | TQ | hypothetical protein |
| LOC_Os08g21879 | 48 | 93-11 | | 0 | TQ | expressed protein |
| LOC_Os08g24360 | 10 | 93-11 | | 0 | TQ | hypothetical protein |
| LOC_Os08g27580 | 18 | 93-11 | | 0 | TQ | expressed protein |
| LOC_Os08g27790 | 71 | 93-11 | | 0 | TQ | retrotransposon protein, putative, unclassified |
| LOC_Os09g15639 | 20 | 93-11 | | 0 | TQ | expressed protein |
| LOC_Os09g15650 | 30 | 93-11 | | 0 | TQ | hypothetical protein |
| LOC_Os09g19229 | 90 | 93-11 | | 0 | TQ | protein kinase domain containing protein, expressed |
| LOC_Os09g24170 | 441 | 93-11 | | 0 | TQ | hypothetical protein |
| LOC_Os09g24190 | 30 | 93-11 | | 0 | TQ | hypothetical protein |
| LOC_Os10g08830 | 14 | 93-11 | | 0 | TQ | conserved hypothetical protein |
| LOC_Os10g10980 | 10 | 93-11 | | 0 | TQ | OsFBX379 - F-box domain containing protein, expressed |
| LOC_Os10g13840 | 11 | 93-11 | | 0 | TQ | conserved hypothetical protein |
| LOC_Os10g17510 | 43 | 93-11 | | 0 | TQ | transposon protein, putative, Ac/Ds sub-class |
| LOC_Os10g20630 | 15 | 93-11 | | 0 | TQ | vacuolar-sorting receptor precursor, putative, expressed |
| LOC_Os10g28510 | 12 | 93-11 | | 0 | TQ | expressed protein |
| LOC_Os11g12350 | 37 | 93-11 | | 0 | TQ | disease resistance protein, putative, expressed |
| LOC_Os11g29090 | 13 | 93-11 | | 0 | TQ | NB-ARC/LRR disease resistance protein, putative |
| LOC_Os11g29110 | 19 | 93-11 | | 0 | TQ | Leucine Rich Repeat family protein, expressed |
| LOC_Os11g29970 | 199 | 93-11 | | 0 | TQ | NB-ARC domain containing protein, expressed |
| LOC_Os11g40249 | 78 | 93-11 | | 0 | TQ | expressed protein |
| LOC_Os11g47452 | 14 | 93-11 | | 0 | TQ | retrotransposon protein, putative, unclassified |
| LOC_Os12g07370 | 109 | 93-11 | | 0 | TQ | csAtPR5, putative, expressed |
| LOC_Os12g07380 | 12 | 93-11 | | 0 | TQ | expressed protein |
| LOC_Os12g07440 | 13 | 93-11 | | 0 | TQ | hypothetical protein |
| LOC_Os12g18260 | 27 | 93-11 | | 0 | TQ | zinc knuckle domain containing protein |
| LOC_Os12g18300 | 30 | 93-11 | | 0 | TQ | retrotransposon protein, putative, unclassified |
| LOC_Os12g24090 | 24 | 93-11 | | 0 | TQ | hypothetical protein |
| LOC_Os12g30760 | 13 | 93-11 | | 0 | TQ | disease resistance protein, putative |
| LOC_Os12g33194 | 17 | 93-11 | | 0 | TQ | expressed protein |
| LOC_Os12g42830 | 23 | 93-11 | | 0 | TQ | conserved hypothetical protein |
