# Supplementary material for: Global RNA sequencing reveals that genotype-dependent allele-specific expression contributes to differential expression in rice F1 hybrids
Source: BMC Plant Biol. 2013 Dec 21;13:221. doi: 10.1186/1471-2229-13-221 (PMC3878109; doi:10.1186/1471-2229-13-221)
Supplement: Additional file 10: Table S6 — List of confirmed monoallelically expressed genes. [file 1471-2229-13-221-S10.docx]

Table S6. List of confirmed monoallelically expressed genes

| **Gene_id** | **Confirmed (Yes or No)** | **Materials** | **Primers (5'to 3' F/R)** |
| --- | --- | --- | --- |
| LOC_Os01g10700 | Yes | GL×TQ & TQ×GL | AATCTGGAACACTTGTCCC |
|  |  |  | GACCTTCAGGAATGCTAAAG |
| LOC_Os01g12304 | Yes | GL×TQ & TQ×GL | CGAAGAGTTTGGTCGTTCC |
|  |  |  | GCCCTGATTGCCACAGAG |
| LOC_Os01g35860 | Yes | GL×TQ & TQ×GL | CCTCCTACTAATCCTCAGAACC |
|  |  |  | TGGTGCTCCTTCAACCTC |
| LOC_Os02g14520 | Yes | GL×TQ & TQ×GL | CCCCTGATTGATGAGTTGTTAG |
|  |  |  | GCATTGACCTTCTTCTTGTCTC |
| LOC_Os02g29210 | Yes | GL×TQ & TQ×GL | cDNA: TGGTGCTGTTGTCAGTGG |
|  |  |  | CTGGGCAAAATGTGAGAAC |
|  |  |  | gDNA: TTCAACAGTCACCACCCC |
|  |  |  | AAAAGTTCTGCCTGCTGC |
| LOC_Os02g38392 | Yes | GL×TQ & TQ×GL | CGGAGAAGTGGATGTTGTAAAC |
|  |  |  | GACGATGACGAGCCTGTG |
| LOC_Os02g40130 | Yes | GL×TQ & TQ×GL | TCTCTCGGAAACCTGACC |
|  |  |  | GGGCATCACCAATGTTAG |
| LOC_Os03g26350 | Yes | GL×TQ & TQ×GL | GTGGAAGGAACATAAAGGG |
|  |  |  | GCCTAATGCTGGAAGGTC |
| LOC_Os03g57560 | Yes | GL×TQ & TQ×GL | GTTCACCTCTTTGGACATTC |
|  |  |  | TCATTGCTCTTCTCCTTAGC |
| LOC_Os04g19970 | Yes | GL×TQ & TQ×GL | AGCAGTGTAGCAGGTGGC |
|  |  |  | GGAAACTTGGTATTTGGTCG |
| LOC_Os04g22960 | No | GL×TQ & TQ×GL | cDNA: TGGTATGGGGTCTCTCTTG |
|  |  |  | GTTCCAGCCAGGGTAATC |
|  |  |  | gDNA: GGTCTCTCTTGTGGACGAC |
|  |  |  | ACTGGCTGATGTGGTAGAAC |
| LOC_Os04g30030 | Yes | GL×TQ & TQ×GL | TGAGAAGGACAGGGATGTATC |
|  |  |  | AGGAGGTAGAGGCAGTGC |
| LOC_Os04g30200 | Yes | GL×TQ & TQ×GL | CCCACAACTGTCCATTACTTC |
|  |  |  | CAGCCTTCAACAACCAGAG |
| LOC_Os04g51250 | Yes | GL×TQ & TQ×GL | GGGAGGTGCTGAAGAAGAG |
|  |  |  | CTCAAGAACTCGTAAAGTGGAG |
| LOC_Os04g52590 | Yes | GL×TQ & TQ×GL | cDNA: ATCACAACAACACGCTCTG |
|  |  |  | TCAGGTATTTGCCCAGTG |
|  |  |  | gDNA: CAGAACAGCAAGATTGAAGC |
|  |  |  | CGTCAGTTTATGATAGGTCGTC |
| LOC_Os05g03320 | Yes | GL×TQ & TQ×GL | cDNA: ATCAGGAGGTGCTTGAGG |
|  |  |  | TCCCAACAAACTAACGCC |
|  |  |  | gDNA: CCAATAATGAACACCGCAG |
|  |  |  | GGAGACCTGGAATGGTTTG |
| LOC_Os05g41880 | No | GL×TQ & TQ×GL | cDNA: GGGGTAAAGAAAAAGGCTAC |
|  |  |  | CCTCCAAGCAGACCAATAC |
|  |  |  | gDNA: CTTGATTGGTATGCGTGC |
|  |  |  | CTTTTCCCTGAGTAGTTAGGTC |
| LOC_Os05g46640 | Yes | GL×TQ & TQ×GL | GTTTTCGTTGAACCAAGTGG |
|  |  |  | TCGGGAATCACAGAAGGC |
| LOC_Os05g46660 | Yes | GL×TQ & TQ×GL | CCTCAACTGGAAAGAATGG |
|  |  |  | TGTCTCTGACTCAATCACTCC |
| LOC_Os05g46680 | Yes | GL×TQ & TQ×GL | TGGTCTCATCAGGGCAAG |
|  |  |  | GCGGTTTCGTAGGAATCG |
| LOC_Os05g48790 | Yes | GL×TQ & TQ×GL | GACTATGGAAGGGAACAGC |
|  |  |  | GCTCTGATGCCTGCTTAC |
| LOC_Os06g13520 | Yes | GL×TQ & TQ×GL | GTGCTAACTTTTCTTGGACG |
|  |  |  | GTGACATTGCTTGCGAAC |
| LOC_Os07g01900 | Yes | GL×TQ & TQ×GL | cDNA: TCGGACCTTCTCCACCAC |
|  |  |  | CTCCTCCATCTAACATTACAGC |
|  |  |  | gDNA: GCTGGTGACTTACCGTCC |
|  |  |  | CCTCCTCCATCTAACATTACAG |
| LOC_Os07g04480 | Yes | GL×TQ & TQ×GL | AACACCCATCCACAGCAC |
|  |  |  | GGCAGACATCCCTTCTACTG |
| LOC_Os07g17689 | Yes | GL×TQ & TQ×GL | GCTACCTCCTCCGCTTAG |
|  |  |  | ACTTCTCTTCCACCGACAG |
| LOC_Os07g19210 | Yes | GL×TQ & TQ×GL | ACAGCGACTACTGGCGTG |
|  |  |  | GACGACGGGAAGAAATCC |
| LOC_Os07g47110 | No | GL×TQ & TQ×GL | TTGAACAAGGAGGAACAGC |
|  |  |  | TGCCGAGATAGGATAGCG |
| LOC_Os08g01520 | Yes | GL×TQ & TQ×GL | CGCAAACCACACATTCTAAC |
|  |  |  | AGGGCACATTCTTCGTCC |
| LOC_Os09g19350 | Yes | GL×TQ & TQ×GL | TCAGCGTGTGAAACAGAAG |
|  |  |  | CAGTTGTGGTGCGATACC |
| LOC_Os09g20040 | Yes | GL×TQ & TQ×GL | CTGCCATTGTTGGAATCTC |
|  |  |  | AGCCTGACCACTACGAAAC |
| LOC_Os09g24180 | Yes | GL×TQ & TQ×GL | GCTGGCAAGGAAATCTTC |
|  |  |  | TTGTCTGATACATCTGCTGC |
| LOC_Os10g04750 | Yes | GL×TQ & TQ×GL | cDNA: CTGGACAACGACGACCTC |
|  |  |  | AAGGCACCAATACGAGTG |
|  |  |  | gDNA: CCAGGAAGGCACCAATAC |
|  |  |  | GTCATCTCAACTGCGTTTTC |
| LOC_Os10g19120 | Yes | GL×TQ & TQ×GL | GCAACTGAAACTACAACGACC |
|  |  |  | TATGACGATGCCAGCAGC |
| LOC_Os10g24050 | Yes | GL×TQ & TQ×GL | GACGACGATGTGGAGTATG |
|  |  |  | AGGGGAGTCTGGTGTGAG |
| LOC_Os10g24980 | Yes | GL×TQ & TQ×GL | GTGCCCGATGATTCTTATTC |
|  |  |  | CATTTTTCCTCTCTTCCTCC |
| LOC_Os10g25180 | Yes | GL×TQ & TQ×GL | GTTATTGAGAAGAAGCCTCG |
|  |  |  | CTCCAAACCCTTAGTCTGC |
| LOC_Os11g07140 | Yes | GL×TQ & TQ×GL | GAACCACATTTCATTGGCTC |
|  |  |  | TCTTGGCAAAGGTCCAGC |
| LOC_Os11g12350 | Yes | GL×TQ & TQ×GL | GAGAAACATTCAGAGTTCCG |
|  |  |  | CCTTAGCACATTGCCCTC |
| LOC_Os11g13680 | Yes | GL×TQ & TQ×GL | TCCTTGTTCTCCACCAGG |
|  |  |  | GGGGAAAACTTACTGCCAC |
| LOC_Os11g35274 | Yes | GL×TQ & TQ×GL | CGTCTTCTGATGCTTTGGG |
|  |  |  | TGACCATTGCCACTCTGC |
| LOC_Os11g44990 | Yes | GL×TQ & TQ×GL | GATGTGGGTTGCTGAAGG |
|  |  |  | CCAATGGACTCTGGGAGAC |
| LOC_Os11g47140 | Yes | GL×TQ & TQ×GL | CGACAACCTTATGCCTAAAG |
|  |  |  | TAAAACGACACTTCCCCG |
| LOC_Os12g02060 | Yes | GL×TQ & TQ×GL | cDNA: CCCTAAACCCTTTGCTTG |
|  |  |  | TTGCTGTTGTTGAGTCGC |
|  |  |  | gDNA: CCCTAAACCCTTTGCTTG |
|  |  |  | TTCTCTCCAGTGAATGTTGG |
| LOC_Os12g02070 | Yes | GL×TQ & TQ×GL | cDNA: ACGGATTGAAGAGGAGATG |
|  |  |  | GTCAGGAGTGGTGAATGTTAC |
|  |  |  | gDNA: CAGAGTTGATTGCGTTGG |
|  |  |  | ACGGATTGAAGAGGAGATG |
| LOC_Os12g20410 | Yes | GL×TQ & TQ×GL | GAATCGCTGAAGCATACC |
|  |  |  | CCTCACCATCCAGTGTCC |
| LOC_Os12g24800 | Yes | GL×TQ & TQ×GL | TTTAGGATGACAGCACACC |
|  |  |  | TCTTCCCAACCATTCGTC |
| LOC_Os05g23960 | Yes | GL×TQ | GGACTCAAATGATGCCCTC |
|  |  |  | GCAACCCAAACTACTGCG |
| LOC_Os07g09460 | Yes | GL×TQ | cDNA: ATGGAGGAAGTCACTGAGG |
|  |  |  | GAGGAAGTAGAACCCGATG |
|  |  |  | gDNA: GCTGTAAAGGACACATTCCC |
|  |  |  | TCGTTGACACTCCCTCTG |
| LOC_Os11g45190 | Yes | GL×TQ | cDNA: TAATCTCCAACCTTCCCC |
|  |  |  | CACACCTAATAAAACACGGC |
|  |  |  | gDNA: GCCCAATGTTTCAGATTCC |
|  |  |  | GTTCCCCTCCTTTTTCCG |
| LOC_Os11g45220 | Yes | GL×TQ | GAACCCAGTGGTGAAGGC |
|  |  |  | ATTATGGCGGAGGCAATG |
| LOC_Os12g30760 | Yes | GL×TQ | ACTGGATGGGACTGGGTC |
|  |  |  | GGGAGATAAAAGAACACAAGCC |
| LOC_Os03g28270 | Yes | TQ×GL | CTTGCTGGGAATCAACTTTC |
|  |  |  | AATGGAGAAGATGCTGGAAG |
| LOC_Os09g18410 | Yes | TQ×GL | GCTTTCCTTATGATGTGGC |
|  |  |  | GGTGGTGCTTGTTTCTCC |
| LOC_Os01g14790 | No | GL×93-11 & 93-11×GL | TCAGAGGGGGAGGTAATG |
|  |  |  | AGCCAGTGAAGTGGATACTC |
| LOC_Os01g20880 | Yes | GL×93-11 & 93-11×GL | TGTAGCCCCTCCCCTTAC |
|  |  |  | CAATCTTCACTCGCTGCTC |
| LOC_Os01g26210 | Yes | GL×93-11 & 93-11×GL | TGCCTATCTCCATTGTGC |
|  |  |  | TCTCTGTTCTCCTCTAAGCC |
| LOC_Os01g26280 | Yes | GL×93-11 & 93-11×GL | ACTGCTAAGTGGGCTTGC |
|  |  |  | AGCGAACTGGACAGAATAAG |
| LOC_Os01g55090 | Yes | GL×93-11 & 93-11×GL | CAATCAGAGAGAAGACAGCAG |
|  |  |  | CAACTAACAGAGAAAACGGG |
| LOC_Os04g19970 | Yes | GL×93-11 & 93-11×GL | AGCAGTGTAGCAGGTGGC |
|  |  |  | GGAAACTTGGTATTTGGTCG |
| LOC_Os04g19980 | Yes | GL×93-11 & 93-11×GL | GCCTAACAAGAGAAATCCC |
|  |  |  | TGAAGTCCAGTGAGTCGG |
| LOC_Os04g23140 | Yes | GL×93-11 & 93-11×GL | CATTCAAGTGCTCCATCG |
|  |  |  | CCCCATTACATAACCAAGG |
| LOC_Os04g24620 | No | GL×93-11 & 93-11×GL | GCATCCAAGGCTCTCAAC |
|  |  |  | CAACCACCGCTACCATTAG |
| LOC_Os04g29680 | Yes | GL×93-11 & 93-11×GL | CCAACTACACCGCTAAGG |
|  |  |  | CTGGAGAAACTGTGCCTG |
| LOC_Os04g30180 | Yes | GL×93-11 & 93-11×GL | CCTTGAGGCTCTAACCAAC |
|  |  |  | CAAACCAGGACAATGCTG |
| LOC_Os04g30240 | Yes | GL×93-11 & 93-11×GL | TGGTGATGTAAAGCCAGC |
|  |  |  | GCAAAGTTGAGACCCTCG |
| LOC_Os04g36580 | Yes | GL×93-11 & 93-11×GL | GGGGAAAACCAAGTTCTG |
|  |  |  | CCAGGAAATGGCAGTATTAC |
| LOC_Os04g52590 | Yes | GL×93-11 & 93-11×GL | cDNA: ATCACAACAACACGCTCTG |
|  |  |  | TCAGGTATTTGCCCAGTG |
|  |  |  | gDNA: CAGAACAGCAAGATTGAAGC |
|  |  |  | CGTCAGTTTATGATAGGTCGTC |
| LOC_Os06g28300 | Yes | GL×93-11 & 93-11×GL | AACCTGTGGGGAAGTTTC |
|  |  |  | AAGGGTGACGGTAAGCAG |
| LOC_Os07g01900 | Yes | GL×93-11 & 93-11×GL | cDNA: TCGGACCTTCTCCACCAC |
|  |  |  | CTCCTCCATCTAACATTACAGC |
|  |  |  | gDNA: GCTGGTGACTTACCGTCC |
|  |  |  | CCTCCTCCATCTAACATTACAG |
| LOC_Os07g45560 | Yes | GL×93-11 & 93-11×GL | CGGCAACCCTATTGTGTC |
|  |  |  | CCTGTAGTTCTCAACCCCTG |
| LOC_Os08g07080 | Yes | GL×93-11 & 93-11×GL | cDNA: TTAGGAAGGTAGTAAAGGGC |
|  |  |  | TTCGTTTCGTGTAGTAGCAC |
|  |  |  | gDNA: AGAGGGGAGTTTTGTTGC |
|  |  |  | GAAAGAGAATAATGGGGCTC |
| LOC_Os08g23020 | Yes | GL×93-11 & 93-11×GL | cDNA: CTACAAGTGTCGTTTTCGC |
|  |  |  | GGGTTGATGTCCAAATCG |
|  |  |  | gDNA: GTTTTGGCATCGTAGACTTG |
|  |  |  | CACCGTTTTGTTGAGGAAC |
| LOC_Os08g23200 | Yes | GL×93-11 & 93-11×GL | GCCTATCTACTCTATGATTCGTGC |
|  |  |  | GCCAACTTTTTATGCTCAGG |
| LOC_Os08g30110 | Yes | GL×93-11 & 93-11×GL | CAGATGACCCAAGAAACG |
|  |  |  | CCACACAAATAACACAACCC |
| LOC_Os10g03000 | Yes | GL×93-11 & 93-11×GL | GTTGGCTTTTGCTGCTTC |
|  |  |  | TTCCCTACGCATAGTCTCAG |
| LOC_Os10g04342 | Yes | GL×93-11 & 93-11×GL | GACCAGTTATCAAGGAAGAGTG |
|  |  |  | CAAAACCAATGTCCACAGG |
| LOC_Os10g24050 | Yes | GL×93-11 & 93-11×GL | GACGACGATGTGGAGTATG |
|  |  |  | AGGGGAGTCTGGTGTGAG |
| LOC_Os10g24980 | Yes | GL×93-11 & 93-11×GL | GTGCCCGATGATTCTTATTC |
|  |  |  | CATTTTTCCTCTCTTCCTCC |
| LOC_Os11g29090 | Yes | GL×93-11 & 93-11×GL | CTGTCAGCATTTGGAGTTG |
|  |  |  | CCACAAGAGAGAGATGAGTTAG |
| LOC_Os11g29110 | Yes | GL×93-11 & 93-11×GL | CCCCAACCTATCAACTTTC |
|  |  |  | TTCGCCATACTTCTCCTTC |
| LOC_Os11g39310 | Yes | GL×93-11 & 93-11×GL | CATCAACAGCAATACGGC |
|  |  |  | CTCTGGAGGCAAGAAACC |
| LOC_Os11g40009 | No | GL×93-11 & 93-11×GL | CTTCTCCACCTACGAGGC |
|  |  |  | CGAGCACTCAACAAATGG |
| LOC_Os11g40249 | Yes | GL×93-11 & 93-11×GL | CACTGAAGAACAGACCCATAAC |
|  |  |  | TTTGAACCTTGGTGAGCC |
| LOC_Os11g41540 | Yes | GL×93-11 & 93-11×GL | CTGATTTGATAGCCCTGC |
|  |  |  | TGAGATACAGTCACCCAAGC |
| LOC_Os11g44990 | Yes | GL×93-11 & 93-11×GL | GATGTGGGTTGCTGAAGG |
|  |  |  | CCAATGGACTCTGGGAGAC |
| LOC_Os12g13270 | Yes | GL×93-11 & 93-11×GL | cDNA: AGAGTCAGTCTGCTGTCCC |
|  |  |  | CTCATCTCGCTTGGAAAG |
|  |  |  | gDNA: AGCCAAGCCTGTTAGACC |
|  |  |  | TCGTCGTTAGAGTTCCGTC |
| LOC_Os12g22284 | Yes | GL×93-11 & 93-11×GL | cDNA: ACCTGAGGAACCCATCCG |
|  |  |  | GCCCAAATAAATGCCCAAAC |
|  |  |  | gDNA: CGAAAGAAGGGAATCCTC |
|  |  |  | TGCTCACGGTAATCGTTC |
| LOC_Os12g24800 | Yes | GL×93-11 & 93-11×GL | TTTAGGATGACAGCACACC |
|  |  |  | TCTTCCCAACCATTCGTC |
| LOC_Os10g10300 | Yes | GL×93-11 | AGAAGGAATCAAGCACGAG |
|  |  |  | AAGAATCCCTCATCCACC |
| LOC_Os11g18810 | Yes | GL×93-11 | TTGAAGCCGATGCTGTAG |
|  |  |  | GCCTTGGTTTGATGATAGG |
| LOC_Os01g31830 | Yes | 93-11×GL | TGAAGGAAGGGAAAGCAG |
|  |  |  | GGAATGATTGAGGAAGAGC |
| LOC_Os04g38060 | Yes | 93-11×GL | GCACCAAAGTCAATCAAGAG |
|  |  |  | CATCAGAGAGCACCTTCATC |
| LOC_Os11g07140 | Yes | 93-11×GL | GAACCACATTTCATTGGCTC |
|  |  |  | TCTTGGCAAAGGTCCAGC |
| LOC_Os01g20880 | Yes | 93-11×TQ & TQ×93-11 | TGTAGCCCCTCCCCTTAC |
|  |  |  | CAATCTTCACTCGCTGCTC |
| LOC_Os01g26210 | Yes | 93-11×TQ & TQ×93-11 | TGCCTATCTCCATTGTGC |
|  |  |  | TCTCTGTTCTCCTCTAAGCC |
| LOC_Os01g33684 | Yes | 93-11×TQ & TQ×93-11 | CGCTATCTTTCTCTGAACTGC |
|  |  |  | GCAATCCACACAAATCTTCC |
| LOC_Os01g40980 | Yes | 93-11×TQ & TQ×93-11 | CATCTTTTGACTTGCTTCCC |
|  |  |  | CCTTCATCCCAGGTCCAG |
| LOC_Os01g42330 | Yes | 93-11×TQ & TQ×93-11 | CGATGAAGTATTCCCCGAG |
|  |  |  | CTGCCACGAAGGTTGTTC |
| LOC_Os01g55090 | Yes | 93-11×TQ & TQ×93-11 | CAATCAGAGAGAAGACAGCAG |
|  |  |  | CAACTAACAGAGAAAACGGG |
| LOC_Os02g14520 | Yes | 93-11×TQ & TQ×93-11 | CCCCTGATTGATGAGTTGTTAG |
|  |  |  | GCATTGACCTTCTTCTTGTCTC |
| LOC_Os03g26080 | Yes | 93-11×TQ & TQ×93-11 | cDNA: CACAAGAATCCTTCCGTG |
|  |  |  | ACACCAAGACTGTCGTGC |
|  |  |  | gDNA: AAAGGTCCTCAACTCTACATCCTCC |
|  |  |  | CTTGCCTCTTGTCATCCAGTGG |
| LOC_Os03g37240 | Yes | 93-11×TQ & TQ×93-11 | CCCAGCCAAACAAAAACAAG |
|  |  |  | GGTTGAATGCGTTGAATGG |
| LOC_Os04g29424 | No | 93-11×TQ & TQ×93-11 | cDNA: GAAAAGTTGGACAAGTGCC |
|  |  |  | TGAATCTTCCCATCACGC |
|  |  |  | gDNA: TATGACCACAGTTCAAGGC |
|  |  |  | TGACCAGAGGACAGTAGGTG |
| LOC_Os04g29680 | Yes | 93-11×TQ & TQ×93-11 | CCAACTACACCGCTAAGG |
|  |  |  | CTGGAGAAACTGTGCCTG |
| LOC_Os04g30240 | Yes | 93-11×TQ & TQ×93-11 | TGGTGATGTAAAGCCAGC |
|  |  |  | GCAAAGTTGAGACCCTCG |
| LOC_Os04g30250 | Yes | 93-11×TQ & TQ×93-11 | GCCTCTCCACAGAAGTGC |
|  |  |  | CCCGTCGTGATGTTTGTC |
| LOC_Os04g37990 | No | 93-11×TQ & TQ×93-11 | CGCCATCTTCCTCTTCTAC |
|  |  |  | CTTGTCAACCGAAACCAC |
| LOC_Os05g03320 | Yes | 93-11×TQ & TQ×93-11 | cDNA: ATCAGGAGGTGCTTGAGG |
|  |  |  | TCCCAACAAACTAACGCC |
|  |  |  | gDNA: CCAATAATGAACACCGCAG |
|  |  |  | GGAGACCTGGAATGGTTTG |
| LOC_Os05g03390 | Yes | 93-11×TQ & TQ×93-11 | GGAGCACAACCATTTCCC |
|  |  |  | CAGTGAAGGACCCCATCC |
| LOC_Os05g15220 | Yes | 93-11×TQ & TQ×93-11 | TCCTGCTGTGATGGTTCG |
|  |  |  | GCTCTGCCCTTGTTTTGC |
| LOC_Os05g15340 | Yes | 93-11×TQ & TQ×93-11 | GCTTGCTTCAGTGGAGTTC |
|  |  |  | GATTACGCCATTCTTGCC |
| LOC_Os05g45580 | No | 93-11×TQ & TQ×93-11 | CAAAGCAAAGAAGGGGTC |
|  |  |  | CTGATGAAGTCTGGATAGGC |
| LOC_Os06g48650 | No | 93-11×TQ & TQ×93-11 | cDNA: TTACCCCCAACATCCAAG |
|  |  |  | CACAGCGGAAAGAAGTGC |
|  |  |  | gDNA: GAAGATAGTTGGTGCCCAG |
|  |  |  | GGAGAGCAAAAGCAAAGG |
| LOC_Os07g17230 | Yes | 93-11×TQ & TQ×93-11 | GATTCCTGGCTATGAACAAC |
|  |  |  | GTCCCCTTAGTCCACTGAG |
| LOC_Os07g17689 | Yes | 93-11×TQ & TQ×93-11 | GCTACCTCCTCCGCTTAG |
|  |  |  | ACTTCTCTTCCACCGACAG |
| LOC_Os07g31250 | Yes | 93-11×TQ & TQ×93-11 | CCACACTTCCTAATAACTGGG |
|  |  |  | GCCTAAGCCGAAACAAAG |
| LOC_Os07g33690 | Yes | 93-11×TQ & TQ×93-11 | CCTGGGAAGTGATTGGTG |
|  |  |  | GAAGCCTGACTGACATTACTC |
| LOC_Os07g45560 | Yes | 93-11×TQ & TQ×93-11 | CGGCAACCCTATTGTGTC |
|  |  |  | CCTGTAGTTCTCAACCCCTG |
| LOC_Os07g45570 | Yes | 93-11×TQ & TQ×93-11 | cDNA: CATTGCGAGAGGTGTTGG |
|  |  |  | CGACCCATCAGTTCTTTTG |
|  |  |  | gDNA: GACATTACTGGTTCCACTACAG |
|  |  |  | GGCAGAGAGAGGTGTGTTAC |
| LOC_Os08g01520 | Yes | 93-11×TQ & TQ×93-11 | CGCAAACCACACATTCTAAC |
|  |  |  | AGGGCACATTCTTCGTCC |
| LOC_Os08g14880 | Yes | 93-11×TQ & TQ×93-11 | TCTGTCTACCACTTTGTGCC |
|  |  |  | CTTCTGATGCTTTCTTCTCC |
| LOC_Os08g14990 | Yes | 93-11×TQ & TQ×93-11 | TCTTGATGTGGCAGAGGC |
|  |  |  | CCCAGATTACTTCGTTGCTC |
| LOC_Os08g27580 | Yes | 93-11×TQ & TQ×93-11 | CCTCCATAGACGGCAGAC |
|  |  |  | GCAGATTACCTCGTAGTGTGG |
| LOC_Os10g04342 | Yes | 93-11×TQ & TQ×93-11 | GACCAGTTATCAAGGAAGAGTG |
|  |  |  | CAAAACCAATGTCCACAGG |
| LOC_Os10g10980 | Yes | 93-11×TQ & TQ×93-11 | CTTGCGTTTACTCATCCG |
|  |  |  | GCTGTTGCCAGAAATACC |
| LOC_Os10g25180 | Yes | 93-11×TQ & TQ×93-11 | GTTATTGAGAAGAAGCCTCG |
|  |  |  | CTCCAAACCCTTAGTCTGC |
| LOC_Os11g07250 | Yes | 93-11×TQ & TQ×93-11 | TCCCATTCAGTCATCTGTTC |
|  |  |  | CGTGTTGTTGCTCAGGTAG |
| LOC_Os11g29090 | Yes | 93-11×TQ & TQ×93-11 | CTGTCAGCATTTGGAGTTG |
|  |  |  | CCACAAGAGAGAGATGAGTTAG |
| LOC_Os11g29110 | Yes | 93-11×TQ & TQ×93-11 | CCCCAACCTATCAACTTTC |
|  |  |  | TTCGCCATACTTCTCCTTC |
| LOC_Os11g40249 | Yes | 93-11×TQ & TQ×93-11 | CACTGAAGAACAGACCCATAAC |
|  |  |  | TTTGAACCTTGGTGAGCC |
| LOC_Os12g18260 | Yes | 93-11×TQ & TQ×93-11 | TGGGCTAAATGGGGAGTG |
|  |  |  | CGTCATCCTTGTCATCCTTATC |
| LOC_Os12g32710 | No | 93-11×TQ & TQ×93-11 | GCAGGACTGGCTGATAATC |
|  |  |  | GAGGGACTCTGGATAATGC |
| LOC_Os06g35600 | Yes | 93-11×TQ | ACACCGATGTGCCTTCAG |
|  |  |  | CACCTTCCTTTGCTTTGG |
| LOC_Os01g31830 | Yes | TQ×93-11 | TGAAGGAAGGGAAAGCAG |
|  |  |  | GGAATGATTGAGGAAGAGC |
