# Supplementary material for: Global RNA sequencing reveals that genotype-dependent allele-specific expression contributes to differential expression in rice F1 hybrids
Source: BMC Plant Biol. 2013 Dec 21;13:221. doi: 10.1186/1471-2229-13-221 (PMC3878109; doi:10.1186/1471-2229-13-221)
Supplement: Additional file 11: Table S7 — List of confirmed preferential allelic expression and biallelic expression genes. [file 1471-2229-13-221-S11.docx]

Table S7. List of confirmed preferential allelic expression and biallelic expression genes

|  | Gene id | Confirmed (Yes or No) | Materials | Primers(5' to 3' F/R) |  |
| --- | --- | --- | --- | --- | --- |
|  | LOC_Os01g09320 | Yes | TQ×GL | GGGGATACTGCTATGTTGTG | |
|  |  |  |  | GACAGCGATTTCAGGGTC | |
|  | LOC_Os05g33100 | Yes | TQ×GL | CCTGAGGAAGATAGCCGAC | |
|  |  |  |  | TTCTGGTGGGGACATACG | |
|  | LOC_Os06g14350 | Yes | GL×TQ & TQ×GL | CATTGACATCCTTGGAGG | |
|  |  |  |  | TCTACTACCATACCAGTGCTTC | |
|  | LOC_Os10g06720 | Yes | GL×TQ & TQ×GL | CGAGAACACGATTCCCAC | |
|  |  |  |  | GATACAACCATCCAGGTCTG | |
|  | LOC_Os11g08110 | Yes | TQ×GL | TGCTTTCACTCACCACTTG | |
|  |  |  |  | GATGCTCAGTTCAGGCAC | |
|  | LOC_Os11g39310 | Yes | GL×TQ & TQ×GL | CATCAACAGCAATACGGC | |
|  |  |  |  | CTCTGGAGGCAAGAAACC | |
|  | LOC_Os02g17450 | Yes | GL×93-11 & 93-11×GL | CAGACTTCAAATGGATTCCC | |
| Preferential |  |  |  | AACCCTGACAGTAGCCCC | |
| allelic | LOC_Os02g42190 | Yes | GL×93-11 & 93-12×GL | AGGTCTCTGACTTTGGTGC | |
| expression |  |  |  | TTCCCTGTGAGCCTCTTC | |
|  | LOC_Os04g29770 | Yes | GL×93-11 & 93-13×GL | GCAAAGGGATGGATGAAAG | |
|  |  |  |  | CATTCGTGCGATTATTGAGC | |
|  | LOC_Os04g35380 | Yes | GL×93-11 & 93-14×GL | CGACCTATTCATTTCTACGC | |
|  |  |  |  | AGGCTTCCCAAAGTAAGG | |
|  | LOC_Os06g06960 | Yes | GL×93-11 & 93-15×GL | TGGGCTCTAAATCTTCCG | |
|  |  |  |  | GAGTTGTAGCAATAGCAAGGAC | |
|  | LOC_Os03g57560 | Yes | 93-11×TQ & TQ×93-11 | GTTCACCTCTTTGGACATTC | |
|  |  |  |  | TCATTGCTCTTCTCCTTAGC | |
|  | LOC_Os06g04350 | Yes | 93-11×TQ & TQ×93-12 | TCCACCAGTAAAGTTCCAAG | |
|  |  |  |  | AGCAAGTGACCACATAATCC | |
|  | LOC_Os06g04690 | Yes | 93-11×TQ & TQ×93-13 | TTCAAGGGGACAAAACGG | |
|  |  |  |  | GGAGTATCACACCTAAAACCAGTC | |
|  | LOC_Os07g43570 | Yes | 93-11×TQ & TQ×93-14 | GGACAAGAAGTAGCAGTAAAGAGG | |
|  |  |  |  | TCTCCAAGACCAGGACGC | |
|  | LOC_Os07g45550 | Yes | 93-11×TQ & TQ×93-15 | ATCAATACCCCATCCAGAC | |
|  |  |  |  | CACAATGTAGAGTGCCCC | |
|  | LOC_Os08g17080 | Yes | 93-11×TQ & TQ×93-16 | CCTGACATCTGAGGTTACTTTC | |
|  |  |  |  | CTCCTGTTTGGGGATTTG | |
|  | LOC_Os12g07970 | No | 93-11×TQ & TQ×93-17 | AACTTCACCTTCCACTATGC | |
|  |  |  |  | ACACCAACTATCCCAGCC | |
|  | LOC_Os01g07370 | Yes | GL×TQ & TQ×GL | GAAGGCTGGACAATGATTC | |
|  |  |  |  | TTCTGATTTCTCGGCGTG | |
|  | LOC_Os01g09540 | Yes | GL×TQ | AACGCCACCGCCACCAATC | |
|  |  |  |  | TCACGACGACGACGACGAGG | |
|  | LOC_Os02g15750 | Yes | GL×TQ & TQ×GL | GAGCCAACCAGCAATCAC | |
|  |  |  |  | GGTCTTCAGCAACTTATGCC | |
|  | LOC_Os02g40510 | Yes | GL×TQ & TQ×GL | AAGCCTTTGAGATGCCAG | |
|  |  |  |  | CTCCGTTCAGACCGACTAC | |
|  | LOC_Os03g03724 | Yes | TQ×GL | GGCATCAGCATCAGCAGC | |
|  |  |  |  | TTTCGCATCTAATCCCCG | |
|  | LOC_Os03g26080 | Yes | GL×TQ & TQ×GL | CACAAGAATCCTTCCGTG | |
|  |  |  |  | ACACCAAGACTGTCGTGC | |
|  | LOC_Os05g47545 | Yes | GL×TQ | AAACCTGGACAGAAAGTGC | |
|  |  |  |  | CGTAAGTCTTCACATCGTGG | |
|  | LOC_Os06g06290 | Yes | GL×TQ & TQ×GL | AGGTGTGGGATGTCAGGC | |
|  |  |  |  | TGGCAACGCAAGTAAACC | |
|  | LOC_Os10g04720 | Yes | GL×TQ & TQ×GL | AACTCCACCACCTCCGTG | |
|  |  |  |  | TCTCTCCCTCCATTTCGC | |
|  | LOC_Os11g09010 | Yes | TQ×GL | ATTCAAGCAGCGGCTATC | |
|  |  |  |  | TGGTGGCAGAGGTGTCAC | |
|  | LOC_Os11g34460 | Yes | GL×TQ & TQ×GL | CACCTGAGAAAGATGGTATGTC | |
|  |  |  |  | ATGAGAGAGTGTGCCCCC | |
| Biallelic | LOC_Os12g19470 | Yes | GL×TQ & TQ×GL | ACTTGCCGCCATTGACAGTG | |
| expression |  |  |  | CCGAACGATAGACCGATAGTTGTAGTAG | |
|  | LOC_Os12g37260 | Yes | GL×TQ & TQ×GL | CGGCTACTTCCCCAATCG | |
|  |  |  |  | GCAGGCTCGCTGTGTTAG | |
|  | LOC_Os03g07530 | Yes | GL×93-11 & 93-13×GL | GCTGTCACGGTCAGACTATG | |
|  |  |  |  | AGAAGAATCAGTGCCCCC | |
|  | LOC_Os03g17570 | Yes | GL×93-11 | TTCTGATGGCTTTGTCCG | |
|  |  |  |  | GCAAGGGAACTGGAGGTC | |
|  | LOC_Os06g46140 | Yes | GL×93-11 & 93-12×GL | CGTGCTGGATTATCTGATG | |
|  |  |  |  | ATCTCTCTCTCTTCTTGAGGAG | |
|  | LOC_Os02g15750 | Yes | 93-11×TQ & TQ×93-11 | GAGCCAACCAGCAATCAC | |
|  |  |  |  | GGTCTTCAGCAACTTATGCC | |
|  | LOC_Os02g56250 | Yes | TQ×93-12 | AGCCCAGCAAGCAGATAC | |
|  |  |  |  | GAGGTAAATGGAAGAACGG | |
|  | LOC_Os03g13810 | Yes | 93-11×TQ & TQ×93-13 | CAGCACATTTTGCCGTAG | |
|  |  |  |  | TTTACACAAGCACACAGCAG | |
|  | LOC_Os03g17570 | Yes | 93-11×TQ & TQ×93-14 | TTCTGATGGCTTTGTCCG | |
|  |  |  |  | GCAAGGGAACTGGAGGTC | |
|  | LOC_Os05g27730 | Yes | TQ×93-15 | ACCACCTACGAGGGCAAG | |
|  |  |  |  | CTGTCTCTCAGCAGTTACAAAG | |
|  | LOC_Os07g47110 | Yes | 93-11×TQ & TQ×93-16 | TTGAACAAGGAGGAACAGC | |
|  |  |  |  | TGCCGAGATAGGATAGCG | |
|  | LOC_Os07g48280 | Yes | 93-11×TQ & TQ×93-17 | GCTCAAATCAGCAGTGGG | |
|  |  |  |  | GTCGTCTTTCAGGTTGTCC | |
|  | LOC_Os08g07540 | Yes | 93-11×TQ | GGCAAAACCCCTTACCTC | |
|  |  |  |  | GCAATCAGCAAGTCAGTGG | |
|  | LOC_Os08g14450 | Yes | 93-11×TQ & TQ×93-19 | TGAGTTCTTCACCATTGTCC | |
|  |  |  |  | GTCTCTCCTCTTTTGCTGTC | |
|  | LOC_Os12g18729 | No | TQ×93-20 | CCCCTGGATTTCGCTGTG | |
|  |  |  |  | GGATTTTGTCTAAACGGACCTC | |
