# Supplementary material for: Global RNA sequencing reveals that genotype-dependent allele-specific expression contributes to differential expression in rice F1 hybrids
Source: BMC Plant Biol. 2013 Dec 21;13:221. doi: 10.1186/1471-2229-13-221 (PMC3878109; doi:10.1186/1471-2229-13-221)
Supplement: Additional file 12: Table S8 — The expression levels of genes with monoallelic expression in the F1 and parental populations. [file 1471-2229-13-221-S12.docx]

Table S8. The expression levels of genes with monoallelic expression in the F1 and parental populations.

| GL×TQ | | | | | | | | | | | | | |
| --- | --- | --- | --- | --- | --- | --- | --- | --- | --- | --- | --- | --- | --- |
| Gene_id | AE ratio-GL | | | | AE ratio-TQ | | | GL-RPKM | GL/TQ-RPKM | | | TQ-RPKM | |
| LOC_Os04g54110 | 100% | | 0% | | | 3.288349 | | | 3.208406 | | 3.664708 | | |
| LOC_Os07g47110 | 100% | | 0% | | | 8.615948 | | | 2.817924 | | 5.771446 | | |
| LOC_Os07g01890 | 100% | | 0% | | | 9.821632 | | | 7.047807 | | 6.186323 | | |
| LOC_Os03g63450 | 100% | | 0% | | | 4.721323 | | | 6.139719 | | 2.735037 | | |
| LOC_Os01g49529 | 0% | | 100% | | | 26.31335 | | | 23.53195 | | 29.49044 | | |
| LOC_Os05g23960 | 0% | | 100% | | | 1.449144 | | | 1.567462 | | 1.762826 | | |
| LOC_Os01g12100 | 0% | | 100% | | | 2.78254 | | | 4.944546 | | 3.63252 | | |
| LOC_Os12g39970 | 0% | | 100% | | | 20.88057 | | | 31.68264 | | 34.90802 | | |
| LOC_Os06g46170 | 0% | | 100% | | | 0.859278 | | | 1.864644 | | 1.582881 | | |
| LOC_Os02g57140 | 100% | | 0% | | | 1.62596 | | | 1.444658 | | 0.699507 | | |
| LOC_Os04g51250 | 100% | | 0% | | | 4.405725 | | | 3.448581 | | 1.481457 | | |
| LOC_Os11g27799 | 100% | | 0% | | | 6.795593 | | | 8.810352 | | 2.255582 | | |
| LOC_Os10g22450 | 100% | | 0% | | | 2.580657 | | | 1.336248 | | 0.851077 | | |
| LOC_Os03g14920 | 100% | | 0% | | | 0.928725 | | | 2.981764 | | 0.208194 | | |
| LOC_Os09g13510 | 100% | | 0% | | | 0.307029 | | | 0.03666 | | 0.056313 | | |
| LOC_Os07g19210 | 100% | | 0% | | | 1.346277 | | | 0.548763 | | 0.234153 | | |
| LOC_Os03g01420 | 100% | | 0% | | | 25.79364 | | | 17.17018 | | 4.463451 | | |
| LOC_Os01g10700 | 100% | | 0% | | | 6.369738 | | | 2.320036 | | 0.701957 | | |
| LOC_Os12g25830 | 0% | | 100% | | | 0.090742 | | | 0.572081 | | 0.366154 | | |
| LOC_Os03g28270 | 0% | | 100% | | | 0.511107 | | | 1.447495 | | 3.464122 | | |
| LOC_Os05g43910 | 0% | | 100% | | | 0.856889 | | | 2.411703 | | 5.886185 | | |
| LOC_Os10g19120 | 0% | | 100% | | | 0.536596 | | | 4.874733 | | 3.789123 | | |
| LOC_Os11g01990 | 0% | | 100% | | | 1.832742 | | | 10.27061 | | 14.66729 | | |
| LOC_Os04g12580 | 0% | | 100% | | | 0.800931 | | | 6.732579 | | 6.517518 | | |
| LOC_Os11g08940 | 0% | | 100% | | | 0.385364 | | | 2.277666 | | 3.174747 | | |
| LOC_Os11g45990 | 100% | | 0% | | | 1.355056 | | | 1.392862 | | 0.118865 | | |
| LOC_Os09g19350 | 100% | | 0% | | | 1.891402 | | | 2.057637 | | 0.115636 | | |
| LOC_Os05g15940 | 100% | | 0% | | | 2.154895 | | | 1.017427 | | 0.12787 | | |
| LOC_Os03g57560 | 100% | | 0% | | | 4.770707 | | | 3.151977 | | 0.277086 | | |
| LOC_Os04g30030 | 100% | | 0% | | | 26.67127 | | | 12.069 | | 1.335442 | | |
| LOC_Os06g12140 | 100% | | 0% | | | 3.766579 | | | 4.387077 | | 0.143382 | | |
| LOC_Os02g05530 | 100% | | 0% | | | 13.01771 | | | 18.40774 | | 0.317707 | | |
| LOC_Os07g16970 | 100% | | 0% | | | 3.094574 | | | 0.429348 | | 0.065952 | | |
| LOC_Os04g22970 | 100% | | 0% | | | 14.6783 | | | 6.105923 | | 0.303957 | | |
| LOC_Os02g38386 | 100% | | 0% | | | 55.76771 | | | 32.25549 | | 0.688158 | | |
| LOC_Os11g45190 | 100% | | 0% | | | 7.283542 | | | 1.097784 | | 0.07227 | | |
| LOC_Os10g10300 | 100% | | 0% | | | 6.984091 | | | 1.931179 | | 0.052973 | | |
| LOC_Os11g12350 | 100% | | 0% | | | 19.40014 | | | 7.455459 | | 0.144965 | | |
| LOC_Os10g24000 | 100% | | 0% | | | 51.47081 | | | 37.81554 | | 0.324514 | | |
| LOC_Os06g15730 | 100% | | 0% | | | 8.73231 | | | 1.954987 | | 0.033367 | | |
| LOC_Os11g39190 | 100% | | 0% | | | 22.95709 | | | 24.04049 | | 0.069545 | | |
| LOC_Os04g22960 | 100% | | 0% | | | 14.54999 | | | 7.986417 | | 0.043814 | | |
| LOC_Os06g40780 | 100% | | 0% | | | 17.97387 | | | 5.320921 | | 0.025865 | | |
| LOC_Os12g07370 | 100% | | 0% | | | 22.05808 | | | 12.67018 | | 0.030315 | | |
| LOC_Os11g29970 | 100% | | 0% | | | 20.72698 | | | 15.72976 | | 0.023923 | | |
| LOC_Os09g19229 | 100% | | 0% | | | 10.56171 | | | 6.364559 | | 0.005737 | | |
| LOC_Os05g13420 | 0% | | 100% | | | 1.248671 | | | 10.48762 | | 16.37511 | | |
| LOC_Os11g40009 | 0% | | 100% | | | 0.102819 | | | 0.405136 | | 1.34837 | | |
| LOC_Os04g52590 | 0% | | 100% | | | 0.133393 | | | 1.423514 | | 1.867059 | | |
| LOC_Os09g19390 | 0% | | 100% | | | 0.643272 | | | 2.615138 | | 11.00064 | | |
| LOC_Os12g02060 | 0% | | 100% | | | 0.425244 | | | 4.244807 | | 9.351634 | | |
| LOC_Os04g23360 | 0% | | 100% | | | 0.037025 | | | 0.94828 | | 1.17652 | | |
| LOC_Os12g28100 | 0% | | 100% | | | 0.074989 | | | 1.32965 | | 2.68546 | | |
| LOC_Os05g41880 | 0% | | 100% | | | 0.069245 | | | 1.382408 | | 2.710261 | | |
| LOC_Os07g04480 | 0% | | 100% | | | 0.188368 | | | 2.22667 | | 8.474907 | | |
| LOC_Os10g22362 | 0% | | 100% | | | 0.214727 | | | 7.332743 | | 10.1807 | | |
| LOC_Os07g04430 | 0% | | 100% | | | 0.196761 | | | 4.177975 | | 9.461212 | | |
| LOC_Os02g38700 | 0% | | 100% | | | 0.057641 | | | 2.611903 | | 2.849186 | | |
| LOC_Os12g21570 | 0% | | 100% | | | 0.339161 | | | 4.603118 | | 23.98751 | | |
| LOC_Os10g25180 | 0% | | 100% | | | 0.036748 | | | 0.868785 | | 2.817345 | | |
| LOC_Os07g10940 | 0% | | 100% | | | 0.027867 | | | 0.823527 | | 3.809091 | | |
| LOC_Os03g26350 | 0% | | 100% | | | 0.041146 | | | 3.945101 | | 10.08621 | | |
| LOC_Os06g38680 | 0% | | 100% | | | 0.064482 | | | 4.785122 | | 18.21333 | | |
| LOC_Os06g12470 | 0% | | 100% | | | 0.030766 | | | 5.394584 | | 10.36597 | | |
| LOC_Os11g07140 | 0% | | 100% | | | 0.123282 | | | 4.533833 | | 58.57538 | | |
| LOC_Os02g29210 | 0% | | 100% | | | 0.032329 | | | 9.957326 | | 17.41517 | | |
| LOC_Os02g40130 | 0% | | 100% | | | 0.473181 | | | 136.9346 | | 712.0189 | | |
| LOC_Os11g10850 | 100% | | 0% | | | 0.456578 | | | 5.097295 | | 0 | | |
| LOC_Os02g31230 | 100% | | 0% | | | 0.505535 | | | 0.663983 | | 0 | | |
| LOC_Os12g28110 | 100% | | 0% | | | 1.632681 | | | 0.964984 | | 0 | | |
| LOC_Os03g01520 | 100% | | 0% | | | 1.738229 | | | 1.327348 | | 0 | | |
| LOC_Os04g30180 | 100% | | 0% | | | 1.747542 | | | 3.884303 | | 0 | | |
| LOC_Os09g18410 | 100% | | 0% | | | 1.872643 | | | 0.81986 | | 0 | | |
| LOC_Os07g01900 | 100% | | 0% | | | 2.524591 | | | 1.04213 | | 0 | | |
| LOC_Os11g41210 | 100% | | 0% | | | 2.694464 | | | 1.203254 | | 0 | | |
| LOC_Os12g07380 | 100% | | 0% | | | 3.010244 | | | 3.234877 | | 0 | | |
| LOC_Os10g15240 | 100% | | 0% | | | 3.026155 | | | 3.71542 | | 0 | | |
| LOC_Os10g24050 | 100% | | 0% | | | 3.891287 | | | 2.862118 | | 0 | | |
| LOC_Os04g12990 | 100% | | 0% | | | 4.405474 | | | 2.143064 | | 0 | | |
| LOC_Os10g24200 | 100% | | 0% | | | 4.482802 | | | 1.998624 | | 0 | | |
| LOC_Os11g25830 | 100% | | 0% | | | 4.580126 | | | 2.685564 | | 0 | | |
| LOC_Os12g30760 | 100% | | 0% | | | 4.696116 | | | 4.829975 | | 0 | | |
| LOC_Os07g17689 | 100% | | 0% | | | 4.703301 | | | 4.60599 | | 0 | | |
| LOC_Os09g15639 | 100% | | 0% | | | 4.819431 | | | 4.298131 | | 0 | | |
| LOC_Os11g35274 | 100% | | 0% | | | 5.774058 | | | 1.759131 | | 0 | | |
| LOC_Os11g18790 | 100% | | 0% | | | 6.103169 | | | 1.970618 | | 0 | | |
| LOC_Os08g01520 | 100% | | 0% | | | 6.480818 | | | 13.76955 | | 0 | | |
| LOC_Os07g07030 | 100% | | 0% | | | 7.00464 | | | 2.830796 | | 0 | | |
| LOC_Os03g13690 | 100% | | 0% | | | 7.711718 | | | 4.006992 | | 0 | | |
| LOC_Os12g29150 | 100% | | 0% | | | 7.992301 | | | 2.51498 | | 0 | | |
| LOC_Os03g01480 | 100% | | 0% | | | 9.803912 | | | 3.863019 | | 0 | | |
| LOC_Os11g18800 | 100% | | 0% | | | 10.00959 | | | 4.38229 | | 0 | | |
| LOC_Os05g03320 | 100% | | 0% | | | 11.14465 | | | 7.141665 | | 0 | | |
| LOC_Os11g45220 | 100% | | 0% | | | 11.45492 | | | 5.628426 | | 0 | | |
| LOC_Os04g31260 | 100% | | 0% | | | 11.82951 | | | 4.097726 | | 0 | | |
| LOC_Os09g15650 | 100% | | 0% | | | 11.82951 | | | 3.359397 | | 0 | | |
| LOC_Os01g35860 | 100% | | 0% | | | 13.1304 | | | 6.299377 | | 0 | | |
| LOC_Os11g18820 | 100% | | 0% | | | 18.35614 | | | 4.55401 | | 0 | | |
| LOC_Os11g18810 | 100% | | 0% | | | 18.4042 | | | 4.291142 | | 0 | | |
| LOC_Os01g35870 | 100% | | 0% | | | 20.58934 | | | 10.09591 | | 0 | | |
| LOC_Os02g38392 | 100% | | 0% | | | 23.29574 | | | 10.51781 | | 0 | | |
| LOC_Os09g24190 | 100% | | 0% | | | 24.39837 | | | 18.39649 | | 0 | | |
| LOC_Os11g13680 | 100% | | 0% | | | 25.63247 | | | 23.19599 | | 0 | | |
| LOC_Os02g18612 | 100% | | 0% | | | 28.50075 | | | 31.00567 | | 0 | | |
| LOC_Os10g03000 | 100% | | 0% | | | 32.68451 | | | 24.85954 | | 0 | | |
| LOC_Os05g46660 | 100% | | 0% | | | 43.51022 | | | 20.61933 | | 0 | | |
| LOC_Os02g57930 | 100% | | 0% | | | 69.5095 | | | 28.37527 | | 0 | | |
| LOC_Os05g46680 | 100% | | 0% | | | 69.9367 | | | 29.78387 | | 0 | | |
| LOC_Os05g46640 | 100% | | 0% | | | 72.18883 | | | 38.82727 | | 0 | | |
| LOC_Os04g23040 | 100% | | 0% | | | 113.6556 | | | 55.33488 | | 0 | | |
| LOC_Os09g24170 | 100% | | 0% | | | 117.0696 | | | 61.25648 | | 0 | | |
| LOC_Os12g24800 | 100% | | 0% | | | 147.3576 | | | 120.3454 | | 0 | | |
| LOC_Os09g24180 | 100% | | 0% | | | 344.5554 | | | 116.2321 | | 0 | | |
| LOC_Os09g20040 | 0% | | 100% | | | 0 | | | 0.753027 | | 0.813987 | | |
| LOC_Os07g04420 | 0% | | 100% | | | 0 | | | 1.735609 | | 2.629024 | | |
| LOC_Os12g32310 | 0% | | 100% | | | 0 | | | 1.554808 | | 3.215054 | | |
| LOC_Os04g30200 | 0% | | 100% | | | 0 | | | 1.162154 | | 3.347201 | | |
| LOC_Os10g29170 | 0% | | 100% | | | 0 | | | 1.290423 | | 3.927703 | | |
| LOC_Os04g51009 | 0% | | 100% | | | 0 | | | 1.214657 | | 4.252976 | | |
| LOC_Os06g39090 | 0% | | 100% | | | 0 | | | 2.548541 | | 4.665569 | | |
| LOC_Os09g19380 | 0% | | 100% | | | 0 | | | 1.421612 | | 5.39865 | | |
| LOC_Os10g04750 | 0% | | 100% | | | 0 | | | 3.819203 | | 6.013305 | | |
| LOC_Os07g19150 | 0% | | 100% | | | 0 | | | 7.322998 | | 6.622268 | | |
| LOC_Os02g14520 | 0% | | 100% | | | 0 | | | 2.949173 | | 7.051029 | | |
| LOC_Os06g07810 | 0% | | 100% | | | 0 | | | 1.755918 | | 7.492354 | | |
| LOC_Os01g59300 | 0% | | 100% | | | 0 | | | 7.536675 | | 8.952409 | | |
| LOC_Os01g12304 | 0% | | 100% | | | 0 | | | 4.379551 | | 9.762913 | | |
| LOC_Os07g09460 | 0% | | 100% | | | 0 | | | 4.698785 | | 9.877511 | | |
| LOC_Os12g02070 | 0% | | 100% | | | 0 | | | 4.887939 | | 11.52138 | | |
| LOC_Os10g24980 | 0% | | 100% | | | 0 | | | 8.936435 | | 13.78079 | | |
| LOC_Os06g13520 | 0% | | 100% | | | 0 | | | 1.592323 | | 13.97686 | | |
| LOC_Os12g21580 | 0% | | 100% | | | 0 | | | 4.040613 | | 13.99819 | | |
| LOC_Os04g19970 | 0% | | 100% | | | 0 | | | 11.87459 | | 14.49464 | | |
| LOC_Os12g20410 | 0% | | 100% | | | 0 | | | 3.820626 | | 16.82396 | | |
| LOC_Os11g44960 | 0% | | 100% | | | 0 | | | 12.15301 | | 22.94753 | | |
| LOC_Os12g36030 | 0% | | 100% | | | 0 | | | 5.746836 | | 23.37918 | | |
| LOC_Os05g48790 | 0% | | 100% | | | 0 | | | 13.83332 | | 24.6241 | | |
| LOC_Os11g44990 | 0% | | 100% | | | 0 | | | 13.37647 | | 26.5334 | | |
| LOC_Os04g19980 | 0% | | 100% | | | 0 | | | 19.19573 | | 29.09839 | | |
| LOC_Os01g09370 | 0% | | 100% | | | 0 | | | 25.39855 | | 42.97245 | | |
| LOC_Os10g20060 | 0% | | 100% | | | 0 | | | 22.91237 | | 68.17492 | | |
| LOC_Os11g47140 | 0% | | 100% | | | 0 | | | 29.87863 | | 80.06703 | | |
| LOC_Os11g07980 | 0% | | 100% | | | 0 | | | 95.33317 | | 184.7145 | | |
| GL×93-11 | | | | | | | | | | | | | |
| Gene_id | AE ratio-93-11 | | | AE ratio-GL | | | 93-11-RPKM | | | GL/93-11-RPKM | | | GL-RPKM |
| LOC_Os08g24850 | 100% | | | 0% | | | 14.48733 | | | 47.34508 | | | 49.81193 |
| LOC_Os01g09320 | 100% | | | 0% | | | 34.17767 | | | 10.50425 | | | 32.19367 |
| LOC_Os08g07080 | 100% | | | 0% | | | 8.028718 | | | 5.834932 | | | 7.401402 |
| LOC_Os01g33960 | 100% | | | 0% | | | 99.19376 | | | 152.1958 | | | 89.71724 |
| LOC_Os03g35970 | 100% | | | 0% | | | 18.93561 | | | 13.09507 | | | 12.79355 |
| LOC_Os11g15450 | 100% | | | 0% | | | 0.208038 | | | 0.536257 | | | 0.095891 |
| LOC_Os09g01980 | 0% | | | 100% | | | 0 | | | 1.328851 | | | 0 |
| LOC_Os03g50290 | 0% | | | 100% | | | 19.47409 | | | 32.47936 | | | 22.7811 |
| LOC_Os01g14790 | 0% | | | 100% | | | 1.107449 | | | 2.854663 | | | 0.935843 |
| LOC_Os04g52590 | 100% | | | 0% | | | 0.759668 | | | 0.686639 | | | 0.133393 |
| LOC_Os11g29110 | 100% | | | 0% | | | 0.638062 | | | 0.847285 | | | 0.098035 |
| LOC_Os01g02440 | 100% | | | 0% | | | 9.814357 | | | 4.220911 | | | 1.49018 |
| LOC_Os08g21879 | 100% | | | 0% | | | 27.88555 | | | 11.68562 | | | 3.475015 |
| LOC_Os04g24510 | 0% | | | 100% | | | 0.17323 | | | 1.299007 | | | 0.585548 |
| LOC_Os04g24620 | 0% | | | 100% | | | 5.127372 | | | 5.08882 | | | 20.85331 |
| LOC_Os12g22060 | 0% | | | 100% | | | 1.170343 | | | 2.861773 | | | 5.394497 |
| LOC_Os01g15270 | 0% | | | 100% | | | 32.69481 | | | 49.7265 | | | 155.7728 |
| LOC_Os11g27799 | 0% | | | 100% | | | 1.284079 | | | 9.829583 | | | 6.795593 |
| LOC_Os02g31230 | 0% | | | 100% | | | 0.073118 | | | 0.873838 | | | 0.505535 |
| LOC_Os12g22284 | 0% | | | 100% | | | 0.301676 | | | 2.697231 | | | 2.13927 |
| LOC_Os01g56850 | 0% | | | 100% | | | 0.585214 | | | 2.386175 | | | 4.423807 |
| LOC_Os03g01420 | 0% | | | 100% | | | 3.272493 | | | 26.68681 | | | 25.79364 |
| LOC_Os04g23799 | 0% | | | 100% | | | 0.47696 | | | 3.613855 | | | 4.763339 |
| LOC_Os11g40009 | 100% | | | 0% | | | 1.609268 | | | 0.537661 | | | 0.102819 |
| LOC_Os08g18079 | 100% | | | 0% | | | 16.21802 | | | 6.29911 | | | 0.79848 |
| LOC_Os04g24274 | 100% | | | 0% | | | 1.244009 | | | 0.893982 | | | 0.050969 |
| LOC_Os11g15670 | 100% | | | 0% | | | 3.664257 | | | 4.554666 | | | 0.097912 |
| LOC_Os10g08830 | 100% | | | 0% | | | 2.807562 | | | 1.95494 | | | 0.073948 |
| LOC_Os07g04480 | 100% | | | 0% | | | 8.336804 | | | 7.067478 | | | 0.188368 |
| LOC_Os04g53496 | 100% | | | 0% | | | 6.197248 | | | 3.864213 | | | 0.132476 |
| LOC_Os08g10250 | 100% | | | 0% | | | 7.541563 | | | 3.682513 | | | 0.102744 |
| LOC_Os07g10940 | 100% | | | 0% | | | 2.131133 | | | 3.357673 | | | 0.027867 |
| LOC_Os07g04390 | 100% | | | 0% | | | 13.16057 | | | 5.478621 | | | 0.142733 |
| LOC_Os08g30110 | 100% | | | 0% | | | 12.33335 | | | 9.626491 | | | 0.129692 |
| LOC_Os01g42330 | 100% | | | 0% | | | 5.566764 | | | 1.992974 | | | 0.047517 |
| LOC_Os04g29680 | 100% | | | 0% | | | 4.325481 | | | 1.450678 | | | 0.036583 |
| LOC_Os11g07140 | 100% | | | 0% | | | 15.04479 | | | 33.46905 | | | 0.123282 |
| LOC_Os07g04430 | 100% | | | 0% | | | 27.88921 | | | 12.30397 | | | 0.196761 |
| LOC_Os07g30980 | 100% | | | 0% | | | 5.694471 | | | 2.954432 | | | 0.038742 |
| LOC_Os06g38680 | 100% | | | 0% | | | 12.24077 | | | 7.867749 | | | 0.064482 |
| LOC_Os10g20080 | 100% | | | 0% | | | 76.74803 | | | 26.18841 | | | 0.310313 |
| LOC_Os12g18320 | 100% | | | 0% | | | 7.838309 | | | 2.968863 | | | 0.029493 |
| LOC_Os11g40249 | 100% | | | 0% | | | 16.32182 | | | 8.633731 | | | 0.058661 |
| LOC_Os12g29150 | 0% | | | 100% | | | 0.541856 | | | 2.666502 | | | 7.992301 |
| LOC_Os12g29160 | 0% | | | 100% | | | 0.215925 | | | 2.992573 | | | 4.151704 |
| LOC_Os08g23020 | 0% | | | 100% | | | 0.582024 | | | 4.767689 | | | 14.0669 |
| LOC_Os05g08900 | 0% | | | 100% | | | 0.523849 | | | 4.151406 | | | 12.73149 |
| LOC_Os06g14350 | 0% | | | 100% | | | 0.035501 | | | 0.873521 | | | 1.407284 |
| LOC_Os12g13270 | 0% | | | 100% | | | 0.220575 | | | 12.44664 | | | 9.204567 |
| LOC_Os06g28300 | 0% | | | 100% | | | 0.07398 | | | 2.600419 | | | 4.296569 |
| LOC_Os11g39310 | 0% | | | 100% | | | 0.146008 | | | 16.57716 | | | 12.95526 |
| LOC_Os08g14880 | 0% | | | 100% | | | 0.300263 | | | 25.45709 | | | 27.65253 |
| LOC_Os01g19140 | 0% | | | 100% | | | 0.093397 | | | 14.2698 | | | 15.92836 |
| LOC_Os10g03000 | 0% | | | 100% | | | 0.190106 | | | 25.1254 | | | 32.68451 |
| LOC_Os02g05530 | 0% | | | 100% | | | 0.05694 | | | 20.25469 | | | 13.01771 |
| LOC_Os10g24000 | 0% | | | 100% | | | 0.174479 | | | 46.12018 | | | 51.47081 |
| LOC_Os04g22960 | 0% | | | 100% | | | 0.047114 | | | 4.835738 | | | 14.54999 |
| LOC_Os10g04342 | 0% | | | 100% | | | 0.041768 | | | 10.86428 | | | 20.46494 |
| LOC_Os11g40400 | 100% | | | 0% | | | 0.375808 | | | 2.113567 | | | 0 |
| LOC_Os11g29090 | 100% | | | 0% | | | 0.797728 | | | 0.373872 | | | 0 |
| LOC_Os12g24100 | 100% | | | 0% | | | 2.78858 | | | 2.631053 | | | 0 |
| LOC_Os04g38490 | 100% | | | 0% | | | 3.275386 | | | 3.070161 | | | 0 |
| LOC_Os11g07150 | 100% | | | 0% | | | 3.510885 | | | 2.131608 | | | 0 |
| LOC_Os10g29170 | 100% | | | 0% | | | 4.460389 | | | 1.886962 | | | 0 |
| LOC_Os06g39090 | 100% | | | 0% | | | 4.815165 | | | 2.878346 | | | 0 |
| LOC_Os08g23090 | 100% | | | 0% | | | 5.061282 | | | 1.920255 | | | 0 |
| LOC_Os11g47452 | 100% | | | 0% | | | 5.198662 | | | 3.330864 | | | 0 |
| LOC_Os12g20410 | 100% | | | 0% | | | 6.857832 | | | 3.549281 | | | 0 |
| LOC_Os07g09460 | 100% | | | 0% | | | 8.010647 | | | 4.1882 | | | 0 |
| LOC_Os10g04750 | 100% | | | 0% | | | 8.043386 | | | 2.882717 | | | 0 |
| LOC_Os12g18300 | 100% | | | 0% | | | 9.495623 | | | 8.383178 | | | 0 |
| LOC_Os04g41000 | 100% | | | 0% | | | 10.52445 | | | 4.71948 | | | 0 |
| LOC_Os10g24980 | 100% | | | 0% | | | 12.16642 | | | 9.404338 | | | 0 |
| LOC_Os01g26210 | 100% | | | 0% | | | 12.40696 | | | 4.840585 | | | 0 |
| LOC_Os07g04490 | 100% | | | 0% | | | 14.29403 | | | 15.07321 | | | 0 |
| LOC_Os01g20880 | 100% | | | 0% | | | 14.71462 | | | 7.174402 | | | 0 |
| LOC_Os03g32330 | 100% | | | 0% | | | 18.69531 | | | 11.00512 | | | 0 |
| LOC_Os08g27580 | 100% | | | 0% | | | 36.07842 | | | 14.68732 | | | 0 |
| LOC_Os08g14860 | 100% | | | 0% | | | 40.70684 | | | 29.7945 | | | 0 |
| LOC_Os04g19970 | 100% | | | 0% | | | 42.38109 | | | 18.46749 | | | 0 |
| LOC_Os01g31830 | 100% | | | 0% | | | 43.85042 | | | 11.25079 | | | 0 |
| LOC_Os11g44990 | 100% | | | 0% | | | 53.91408 | | | 13.22237 | | | 0 |
| LOC_Os08g21530 | 100% | | | 0% | | | 56.85351 | | | 33.07733 | | | 0 |
| LOC_Os04g19980 | 100% | | | 0% | | | 66.75241 | | | 26.20115 | | | 0 |
| LOC_Os10g19990 | 100% | | | 0% | | | 139.3159 | | | 56.19647 | | | 0 |
| LOC_Os10g20060 | 100% | | | 0% | | | 168.4871 | | | 80.21334 | | | 0 |
| LOC_Os11g36560 | 0% | | | 100% | | | 0 | | | 0.457777 | | | 0.207793 |
| LOC_Os01g25450 | 0% | | | 100% | | | 0 | | | 0.29918 | | | 0.219699 |
| LOC_Os12g10770 | 0% | | | 100% | | | 0 | | | 0.983712 | | | 0.994344 |
| LOC_Os08g41630 | 0% | | | 100% | | | 0 | | | 2.8385 | | | 1.17941 |
| LOC_Os12g22010 | 0% | | | 100% | | | 0 | | | 1.261201 | | | 1.493045 |
| LOC_Os04g30180 | 0% | | | 100% | | | 0 | | | 4.032154 | | | 1.747542 |
| LOC_Os04g30240 | 0% | | | 100% | | | 0 | | | 2.877863 | | | 2.225114 |
| LOC_Os10g04720 | 0% | | | 100% | | | 0 | | | 7.654641 | | | 2.509416 |
| LOC_Os07g01900 | 0% | | | 100% | | | 0 | | | 5.170624 | | | 2.524591 |
| LOC_Os10g03669 | 0% | | | 100% | | | 0 | | | 8.081064 | | | 2.535266 |
| LOC_Os08g23200 | 0% | | | 100% | | | 0 | | | 2.108957 | | | 2.547183 |
| LOC_Os11g41210 | 0% | | | 100% | | | 0 | | | 1.808203 | | | 2.694464 |
| LOC_Os07g45560 | 0% | | | 100% | | | 0 | | | 1.249704 | | | 2.928076 |
| LOC_Os10g15240 | 0% | | | 100% | | | 0 | | | 3.913091 | | | 3.026155 |
| LOC_Os06g42650 | 0% | | | 100% | | | 0 | | | 4.756793 | | | 3.075516 |
| LOC_Os10g24050 | 0% | | | 100% | | | 0 | | | 3.086162 | | | 3.891287 |
| LOC_Os10g24200 | 0% | | | 100% | | | 0 | | | 1.379964 | | | 4.482802 |
| LOC_Os04g23140 | 0% | | | 100% | | | 0 | | | 11.3448 | | | 4.958879 |
| LOC_Os11g18780 | 0% | | | 100% | | | 0 | | | 1.082959 | | | 5.124719 |
| LOC_Os01g15510 | 0% | | | 100% | | | 0 | | | 8.542208 | | | 5.414082 |
| LOC_Os10g10750 | 0% | | | 100% | | | 0 | | | 7.0825 | | | 5.451304 |
| LOC_Os01g26280 | 0% | | | 100% | | | 0 | | | 4.376711 | | | 6.417535 |
| LOC_Os10g10300 | 0% | | | 100% | | | 0 | | | 4.925559 | | | 6.984091 |
| LOC_Os11g45190 | 0% | | | 100% | | | 0 | | | 2.513124 | | | 7.283542 |
| LOC_Os03g04720 | 0% | | | 100% | | | 0 | | | 1.834445 | | | 8.419388 |
| LOC_Os01g55090 | 0% | | | 100% | | | 0 | | | 6.342073 | | | 8.472797 |
| LOC_Os06g15730 | 0% | | | 100% | | | 0 | | | 4.994391 | | | 8.73231 |
| LOC_Os04g36580 | 0% | | | 100% | | | 0 | | | 18.58676 | | | 9.173559 |
| LOC_Os04g38060 | 0% | | | 100% | | | 0 | | | 2.55168 | | | 9.434037 |
| LOC_Os03g01480 | 0% | | | 100% | | | 0 | | | 8.020119 | | | 9.803912 |
| LOC_Os11g18800 | 0% | | | 100% | | | 0 | | | 4.71187 | | | 10.00959 |
| LOC_Os01g32439 | 0% | | | 100% | | | 0 | | | 5.29465 | | | 10.30247 |
| LOC_Os04g22970 | 0% | | | 100% | | | 0 | | | 6.89339 | | | 14.6783 |
| LOC_Os12g13295 | 0% | | | 100% | | | 0 | | | 13.85532 | | | 16.54471 |
| LOC_Os10g04730 | 0% | | | 100% | | | 0 | | | 43.9271 | | | 17.02234 |
| LOC_Os11g41540 | 0% | | | 100% | | | 0 | | | 14.05339 | | | 17.70164 |
| LOC_Os11g18820 | 0% | | | 100% | | | 0 | | | 4.168365 | | | 18.35614 |
| LOC_Os11g18810 | 0% | | | 100% | | | 0 | | | 5.572677 | | | 18.4042 |
| LOC_Os11g40160 | 0% | | | 100% | | | 0 | | | 16.07986 | | | 19.48145 |
| LOC_Os12g29690 | 0% | | | 100% | | | 0 | | | 10.0221 | | | 21.67303 |
| LOC_Os04g22950 | 0% | | | 100% | | | 0 | | | 16.26454 | | | 24.56312 |
| LOC_Os04g23040 | 0% | | | 100% | | | 0 | | | 62.13864 | | | 113.6556 |
| LOC_Os12g24800 | 0% | | | 100% | | | 0 | | | 35.90403 | | | 147.3576 |
| 93-11×TQ | | | | | | | | | | | | | |
| Gene_id | | AE ratio-93-11 | | | AE ratio-TQ | | | 93-11-RPKM | | 93-11/TQ-RPKM | | | TQ-RPKM |
| LOC_Os03g37240 | | 100% | | | 0% | | | 15.89784 | | 18.02114 | | | 22.98276 |
| LOC_Os11g29110 | | 100% | | | 0% | | | 0.638062 | | 0.847294 | | | 0.758192 |
| LOC_Os03g35970 | | 100% | | | 0% | | | 18.93561 | | 19.16584 | | | 16.46203 |
| LOC_Os10g20630 | | 100% | | | 0% | | | 16.1499 | | 12.59269 | | | 11.52676 |
| LOC_Os10g01380 | | 0% | | | 100% | | | 6.098564 | | 6.582173 | | | 3.822555 |
| LOC_Os12g29160 | | 0% | | | 100% | | | 0.215925 | | 3.981468 | | | 0.315543 |
| LOC_Os12g32710 | | 0% | | | 100% | | | 0.973989 | | 1.432665 | | | 1.463157 |
| LOC_Os01g29469 | | 0% | | | 100% | | | 16.10357 | | 15.15548 | | | 25.20455 |
| LOC_Os04g37990 | | 0% | | | 100% | | | 0.310835 | | 0.660421 | | | 0.562064 |
| LOC_Os06g48650 | | 100% | | | 0% | | | 2.362665 | | 1.574265 | | | 0.972197 |
| LOC_Os05g45580 | | 100% | | | 0% | | | 1.140007 | | 1.114733 | | | 0.433698 |
| LOC_Os12g07440 | | 100% | | | 0% | | | 4.027108 | | 2.773537 | | | 1.523397 |
| LOC_Os01g40980 | | 100% | | | 0% | | | 1.23045 | | 1.054352 | | | 0.431796 |
| LOC_Os10g17510 | | 100% | | | 0% | | | 8.142842 | | 5.532955 | | | 2.534329 |
| LOC_Os06g14040 | | 100% | | | 0% | | | 5.040006 | | 1.755175 | | | 1.365738 |
| LOC_Os05g45590 | | 100% | | | 0% | | | 4.267599 | | 1.542954 | | | 0.900453 |
| LOC_Os07g23900 | | 100% | | | 0% | | | 2.396043 | | 3.245383 | | | 0.495157 |
| LOC_Os08g24360 | | 100% | | | 0% | | | 3.802313 | | 3.42566 | | | 0.761593 |
| LOC_Os08g17140 | | 100% | | | 0% | | | 3.420608 | | 1.533022 | | | 0.596438 |
| LOC_Os04g29680 | | 100% | | | 0% | | | 4.325481 | | 1.233091 | | | 0.664265 |
| LOC_Os06g40770 | | 100% | | | 0% | | | 6.043973 | | 2.834463 | | | 0.782616 |
| LOC_Os01g15270 | | 0% | | | 100% | | | 32.69481 | | 60.4902 | | | 106.4161 |
| LOC_Os01g59570 | | 0% | | | 100% | | | 0.438214 | | 1.280206 | | | 2.505481 |
| LOC_Os12g25830 | | 0% | | | 100% | | | 0.05906 | | 0.470561 | | | 0.366154 |
| LOC_Os12g37260 | | 0% | | | 100% | | | 2.903874 | | 9.204359 | | | 19.27897 |
| LOC_Os07g17230 | | 0% | | | 100% | | | 0.750817 | | 2.887032 | | | 5.160784 |
| LOC_Os08g29400 | | 0% | | | 100% | | | 0.466495 | | 1.3938 | | | 3.723603 |
| LOC_Os02g18780 | | 100% | | | 0% | | | 1.731945 | | 2.291667 | | | 0.172567 |
| LOC_Os08g21879 | | 100% | | | 0% | | | 27.88555 | | 12.42968 | | | 2.196214 |
| LOC_Os02g10340 | | 100% | | | 0% | | | 1.377438 | | 0.566729 | | | 0.104996 |
| LOC_Os07g15930 | | 100% | | | 0% | | | 1.106086 | | 0.864327 | | | 0.079124 |
| LOC_Os12g42830 | | 100% | | | 0% | | | 2.709048 | | 2.077897 | | | 0.188006 |
| LOC_Os02g12450 | | 100% | | | 0% | | | 1.05479 | | 0.450217 | | | 0.070065 |
| LOC_Os08g18079 | | 100% | | | 0% | | | 16.21802 | | 7.92437 | | | 1.027686 |
| LOC_Os07g28644 | | 100% | | | 0% | | | 2.026127 | | 1.362081 | | | 0.117763 |
| LOC_Os10g08830 | | 100% | | | 0% | | | 2.807562 | | 1.182376 | | | 0.124329 |
| LOC_Os10g28510 | | 100% | | | 0% | | | 3.936838 | | 1.710916 | | | 0.149771 |
| LOC_Os08g27790 | | 100% | | | 0% | | | 8.063238 | | 5.266506 | | | 0.305169 |
| LOC_Os07g30980 | | 100% | | | 0% | | | 5.694471 | | 3.114007 | | | 0.146557 |
| LOC_Os07g45570 | | 100% | | | 0% | | | 18.55861 | | 8.484342 | | | 0.469166 |
| LOC_Os06g12140 | | 100% | | | 0% | | | 6.398549 | | 4.146004 | | | 0.143382 |
| LOC_Os01g33684 | | 100% | | | 0% | | | 3.734974 | | 1.853597 | | | 0.073588 |
| LOC_Os12g33194 | | 100% | | | 0% | | | 8.782887 | | 5.301883 | | | 0.166687 |
| LOC_Os08g10250 | | 100% | | | 0% | | | 7.541563 | | 3.507568 | | | 0.120919 |
| LOC_Os11g12350 | | 100% | | | 0% | | | 19.95323 | | 7.824676 | | | 0.144965 |
| LOC_Os03g32330 | | 100% | | | 0% | | | 18.69531 | | 11.56666 | | | 0.122954 |
| LOC_Os08g21530 | | 100% | | | 0% | | | 56.85351 | | 30.84955 | | | 0.303857 |
| LOC_Os05g03390 | | 100% | | | 0% | | | 27.27539 | | 10.64837 | | | 0.145045 |
| LOC_Os01g42330 | | 100% | | | 0% | | | 5.566764 | | 2.340865 | | | 0.023967 |
| LOC_Os11g40249 | | 100% | | | 0% | | | 16.32182 | | 7.814062 | | | 0.044382 |
| LOC_Os07g23270 | | 100% | | | 0% | | | 35.1842 | | 16.68464 | | | 0.083045 |
| LOC_Os06g40780 | | 100% | | | 0% | | | 11.48698 | | 3.756193 | | | 0.025865 |
| LOC_Os12g07370 | | 100% | | | 0% | | | 23.76465 | | 20.96042 | | | 0.030315 |
| LOC_Os11g29970 | | 100% | | | 0% | | | 33.05673 | | 15.61832 | | | 0.023923 |
| LOC_Os09g19229 | | 100% | | | 0% | | | 23.48754 | | 7.830563 | | | 0.005737 |
| LOC_Os11g07250 | | 0% | | | 100% | | | 0.219114 | | 0.576113 | | | 2.404439 |
| LOC_Os11g08940 | | 0% | | | 100% | | | 0.278684 | | 3.830222 | | | 3.174747 |
| LOC_Os10g04060 | | 0% | | | 100% | | | 0.093541 | | 0.857083 | | | 1.62379 |
| LOC_Os04g29424 | | 0% | | | 100% | | | 0.223901 | | 3.134624 | | | 4.580778 |
| LOC_Os11g08830 | | 0% | | | 100% | | | 0.897162 | | 19.83611 | | | 23.49996 |
| LOC_Os06g06960 | | 0% | | | 100% | | | 0.263543 | | 6.866279 | | | 8.136731 |
| LOC_Os08g05440 | | 0% | | | 100% | | | 0.012083 | | 0.332136 | | | 0.415754 |
| LOC_Os06g35600 | | 0% | | | 100% | | | 0.061666 | | 1.031782 | | | 2.236512 |
| LOC_Os12g36400 | | 0% | | | 100% | | | 1.940255 | | 53.33353 | | | 80.74441 |
| LOC_Os12g28100 | | 0% | | | 100% | | | 0.040672 | | 1.190912 | | | 2.68546 |
| LOC_Os11g29490 | | 0% | | | 100% | | | 0.041177 | | 1.451733 | | | 2.929362 |
| LOC_Os12g16520 | | 0% | | | 100% | | | 0.104752 | | 6.071814 | | | 13.58932 |
| LOC_Os05g43910 | | 0% | | | 100% | | | 0.044263 | | 1.957279 | | | 5.886185 |
| LOC_Os08g14880 | | 0% | | | 100% | | | 0.300263 | | 17.65551 | | | 47.27365 |
| LOC_Os07g33690 | | 0% | | | 100% | | | 0.049755 | | 1.84335 | | | 11.96062 |
| LOC_Os11g07720 | | 0% | | | 100% | | | 0.048077 | | 4.366797 | | | 13.05507 |
| LOC_Os01g19140 | | 0% | | | 100% | | | 0.093397 | | 16.85475 | | | 27.56182 |
| LOC_Os10g04342 | | 0% | | | 100% | | | 0.041768 | | 11.10665 | | | 23.93629 |
| LOC_Os12g35465 | | 0% | | | 100% | | | 0.050108 | | 53.11807 | | | 71.43445 |
| LOC_Os11g07980 | | 0% | | | 100% | | | 0.090989 | | 68.50794 | | | 184.7145 |
| LOC_Os10g13840 | | 100% | | | 0% | | | 0.568926 | | 0.930442 | | | 0 |
| LOC_Os11g29090 | | 100% | | | 0% | | | 0.797728 | | 0.317795 | | | 0 |
| LOC_Os08g15830 | | 100% | | | 0% | | | 1.145087 | | 0.603328 | | | 0 |
| LOC_Os02g16995 | | 100% | | | 0% | | | 4.288519 | | 3.075193 | | | 0 |
| LOC_Os11g47452 | | 100% | | | 0% | | | 5.198662 | | 3.539083 | | | 0 |
| LOC_Os07g07030 | | 100% | | | 0% | | | 5.296911 | | 3.623652 | | | 0 |
| LOC_Os12g18260 | | 100% | | | 0% | | | 5.379044 | | 1.995094 | | | 0 |
| LOC_Os12g07380 | | 100% | | | 0% | | | 6.23391 | | 5.144261 | | | 0 |
| LOC_Os09g15639 | | 100% | | | 0% | | | 6.325124 | | 3.625389 | | | 0 |
| LOC_Os12g30760 | | 100% | | | 0% | | | 6.468751 | | 3.285664 | | | 0 |
| LOC_Os12g24090 | | 100% | | | 0% | | | 6.758733 | | 5.281467 | | | 0 |
| LOC_Os10g10980 | | 100% | | | 0% | | | 7.301045 | | 3.520884 | | | 0 |
| LOC_Os08g07080 | | 100% | | | 0% | | | 8.028718 | | 1.880166 | | | 0 |
| LOC_Os12g18300 | | 100% | | | 0% | | | 9.495623 | | 4.275469 | | | 0 |
| LOC_Os07g17689 | | 100% | | | 0% | | | 9.801086 | | 5.990483 | | | 0 |
| LOC_Os08g01520 | | 100% | | | 0% | | | 10.61408 | | 8.484237 | | | 0 |
| LOC_Os09g15650 | | 100% | | | 0% | | | 11.7917 | | 6.079145 | | | 0 |
| LOC_Os01g26210 | | 100% | | | 0% | | | 12.40696 | | 7.064593 | | | 0 |
| LOC_Os01g24200 | | 100% | | | 0% | | | 12.90383 | | 6.511385 | | | 0 |
| LOC_Os01g20880 | | 100% | | | 0% | | | 14.71462 | | 7.268335 | | | 0 |
| LOC_Os01g21130 | | 100% | | | 0% | | | 16.04479 | | 9.709138 | | | 0 |
| LOC_Os05g03320 | | 100% | | | 0% | | | 23.93082 | | 12.21501 | | | 0 |
| LOC_Os05g20460 | | 100% | | | 0% | | | 26.96191 | | 19.66544 | | | 0 |
| LOC_Os08g27580 | | 100% | | | 0% | | | 36.07842 | | 21.87386 | | | 0 |
| LOC_Os01g31830 | | 100% | | | 0% | | | 43.85042 | | 14.15366 | | | 0 |
| LOC_Os09g24190 | | 100% | | | 0% | | | 45.09071 | | 16.40103 | | | 0 |
| LOC_Os09g24170 | | 100% | | | 0% | | | 114.3185 | | 71.01065 | | | 0 |
| LOC_Os12g17470 | | 0% | | | 100% | | | 0 | | 0.062089 | | | 0.096626 |
| LOC_Os05g46110 | | 0% | | | 100% | | | 0 | | 1.267631 | | | 0.157819 |
| LOC_Os01g25450 | | 0% | | | 100% | | | 0 | | 0.732401 | | | 0.269118 |
| LOC_Os05g11290 | | 0% | | | 100% | | | 0 | | 1.618488 | | | 0.775003 |
| LOC_Os12g22080 | | 0% | | | 100% | | | 0 | | 0.745737 | | | 0.787513 |
| LOC_Os11g36970 | | 0% | | | 100% | | | 0 | | 0.746018 | | | 1.067356 |
| LOC_Os01g15910 | | 0% | | | 100% | | | 0 | | 0.882122 | | | 1.372795 |
| LOC_Os04g30240 | | 0% | | | 100% | | | 0 | | 1.476958 | | | 1.436564 |
| LOC_Os08g14990 | | 0% | | | 100% | | | 0 | | 3.76845 | | | 2.497892 |
| LOC_Os06g34460 | | 0% | | | 100% | | | 0 | | 3.732751 | | | 2.717142 |
| LOC_Os10g25180 | | 0% | | | 100% | | | 0 | | 0.809895 | | | 2.817345 |
| LOC_Os04g30250 | | 0% | | | 100% | | | 0 | | 1.61378 | | | 2.861863 |
| LOC_Os07g31250 | | 0% | | | 100% | | | 0 | | 0.875889 | | | 3.053333 |
| LOC_Os07g45560 | | 0% | | | 100% | | | 0 | | 1.280961 | | | 3.294111 |
| LOC_Os12g10770 | | 0% | | | 100% | | | 0 | | 2.264613 | | | 3.578504 |
| LOC_Os05g15340 | | 0% | | | 100% | | | 0 | | 2.738082 | | | 3.787659 |
| LOC_Os12g22010 | | 0% | | | 100% | | | 0 | | 1.340041 | | | 3.846454 |
| LOC_Os10g03900 | | 0% | | | 100% | | | 0 | | 2.767966 | | | 4.249411 |
| LOC_Os07g19150 | | 0% | | | 100% | | | 0 | | 5.596007 | | | 6.622268 |
| LOC_Os08g10760 | | 0% | | | 100% | | | 0 | | 5.729723 | | | 6.687624 |
| LOC_Os02g14520 | | 0% | | | 100% | | | 0 | | 4.46038 | | | 7.051029 |
| LOC_Os12g12514 | | 0% | | | 100% | | | 0 | | 3.41573 | | | 7.319005 |
| LOC_Os06g07810 | | 0% | | | 100% | | | 0 | | 2.631867 | | | 7.492354 |
| LOC_Os04g38060 | | 0% | | | 100% | | | 0 | | 3.19318 | | | 8.34477 |
| LOC_Os01g59300 | | 0% | | | 100% | | | 0 | | 6.05292 | | | 8.952409 |
| LOC_Os03g26080 | | 0% | | | 100% | | | 0 | | 3.1573 | | | 9.110485 |
| LOC_Os03g26350 | | 0% | | | 100% | | | 0 | | 4.027374 | | | 10.08621 |
| LOC_Os06g12470 | | 0% | | | 100% | | | 0 | | 5.484274 | | | 10.36597 |
| LOC_Os01g55090 | | 0% | | | 100% | | | 0 | | 6.039343 | | | 11.03871 |
| LOC_Os05g15330 | | 0% | | | 100% | | | 0 | | 9.674969 | | | 11.36088 |
| LOC_Os01g32439 | | 0% | | | 100% | | | 0 | | 5.952278 | | | 11.40953 |
| LOC_Os05g13410 | | 0% | | | 100% | | | 0 | | 9.405085 | | | 13.43231 |
| LOC_Os06g13520 | | 0% | | | 100% | | | 0 | | 2.844037 | | | 13.97686 |
| LOC_Os12g29690 | | 0% | | | 100% | | | 0 | | 10.12551 | | | 15.93218 |
| LOC_Os05g13420 | | 0% | | | 100% | | | 0 | | 12.18362 | | | 16.37511 |
| LOC_Os12g36030 | | 0% | | | 100% | | | 0 | | 7.174707 | | | 23.37918 |
| LOC_Os12g21570 | | 0% | | | 100% | | | 0 | | 5.129774 | | | 23.98751 |
| LOC_Os05g15220 | | 0% | | | 100% | | | 0 | | 8.168591 | | | 27.0458 |
| LOC_Os10g04730 | | 0% | | | 100% | | | 0 | | 8.341222 | | | 39.70945 |

*AE: allelic expression
