# Supplementary material for: Global RNA sequencing reveals that genotype-dependent allele-specific expression contributes to differential expression in rice F1 hybrids
Source: BMC Plant Biol. 2013 Dec 21;13:221. doi: 10.1186/1471-2229-13-221 (PMC3878109; doi:10.1186/1471-2229-13-221)
Supplement: Additional file 13: Table S9 — The expression changes of monoallelic expression genes between F1 and parents. [file 1471-2229-13-221-S13.docx]

| F1  hybrids | expressed allele | total  number | comparison | down regulated | % | up regulated | % |
| --- | --- | --- | --- | --- | --- | --- | --- |
| GLXTQ | GL | 80 | F1 vs GL | 64 | 80.0% | 16 | 20.0% |
|  | TQ | 63 | F1 vs TQ | 57 | 90.5% | 6 | 9.5% |
| GLX93-11 | 93-11 | 58 | F1 vs 93-11 | 49 | 84.5% | 9 | 15.5% |
|  | GL | 71 | F1 vs GL | 45 | 63.4% | 26 | 36.6% |
| 93-11XTQ | 93-11 | 71 | F1 vs 93-11 | 65 | 91.5% | 6 | 8.5% |
|  | TQ | 70 | F1 vs TQ | 59 | 84.3% | 11 | 15.7% |

Table S9. The expression changes of monoallelic expression genes between F1 and parents
