# Supplementary material for: Global RNA sequencing reveals that genotype-dependent allele-specific expression contributes to differential expression in rice F1 hybrids
Source: BMC Plant Biol. 2013 Dec 21;13:221. doi: 10.1186/1471-2229-13-221 (PMC3878109; doi:10.1186/1471-2229-13-221)
Supplement: Additional file 14: Table S10 — Allelic expression genes classified by the relative expression level of parents. [file 1471-2229-13-221-S14.docx]

Table S10. Allelic expression genes classified by the relative expression level of parents

|  | Monoallelic  expression | % | Preferential  allelic expression | % | Biallelic  expression | % | Total | % |
| --- | --- | --- | --- | --- | --- | --- | --- | --- |
|  | GL×TQ | | | | | | | |
| I | 9 | 0.4% | 396 | 19.2% | 1662 | 80.4% | 2067 | 57.0% |
| II | 16 | 1.3% | 363 | 28.4% | 900 | 70.4% | 1279 | 35.3% |
| III | 42 | 22.8% | 79 | 42.9% | 63 | 34.2% | 184 | 5.1% |
| IV | 76 | 78.4% | 13 | 13.4% | 8 | 8.2% | 97 | 2.7% |
| Total | 143 |  | 851 |  | 2633 |  | 3627 |  |
|  | GL×93-11 | | | | | | | |
| I | 9 | 0.4% | 460 | 18.6% | 2001 | 81.0% | 2470 | 64.6% |
| II | 14 | 1.3% | 330 | 30.9% | 724 | 67.8% | 1068 | 27.9% |
| III | 35 | 18.7% | 98 | 52.4% | 54 | 28.9% | 187 | 4.9% |
| IV | 71 | 71.7% | 17 | 17.2% | 11 | 11.1% | 99 | 2.6% |
| Total | 129 |  | 905 |  | 2790 |  | 3824 |  |
|  | 93-11×TQ | | | | | | | |
| I | 9 | 0.3% | 550 | 19.7% | 2230 | 80.0% | 2789 | 74.9% |
| II | 18 | 2.4% | 305 | 40.1% | 437 | 57.5% | 760 | 20.4% |
| III | 48 | 49.0% | 35 | 35.7% | 15 | 15.3% | 98 | 2.6% |
| IV | 66 | 83.5% | 13 | 16.5% | 0 | 0.0% | 79 | 2.1% |
| Total | 141 |  | 903 |  | 2682 |  | 3726 |  |

Notes: The data corresponding for the groups of I, II, III and IV in Figure 4.
