# Supplementary material for: Global RNA sequencing reveals that genotype-dependent allele-specific expression contributes to differential expression in rice F1 hybrids
Source: BMC Plant Biol. 2013 Dec 21;13:221. doi: 10.1186/1471-2229-13-221 (PMC3878109; doi:10.1186/1471-2229-13-221)
Supplement: Additional file 15: Table S11 — Molecular function of monoallelic expression genes. [file 1471-2229-13-221-S15.docx]

Table S11. Molecular function of monoallelic expression genes

| **GO Term** | **GL**×**TQ** | | **GL**×**93-11** | | **93-11**×**TQ** | |
| --- | --- | --- | --- | --- | --- | --- |
|  | **Genes** | **P value** | **Genes** | **P value** | **Genes** | **P value** |
| nucleotide binding | 26 | 1.87E-08 | 27 | 2.20E-10 | 37 | 1.52E-14 |
| receptor activity | 8 | 3.77E-05 | 12 | 1.23E-09 | 7 | 5.85E-04 |
| protein binding | 25 | 5.51E-10 | 41 | 1.70E-26 | 46 | 1.98E-26 |
| kinase activity | 22 | 5.65E-09 | 22 | 4.85E-10 | 28 | 3.30E-12 |
| transcription factor activity | 17 | 1.49E-05 |  |  | 12 | 0.01 |
| ATP-dependent DNA helicase activity |  |  | 1 | 0.01 | 1 | 0.01 |
| monooxygenase activity | 2 | 0.02 |  |  | 2 | 0.02 |
| protein serine/threonine kinase activity |  |  | 3 | 0.05 | 5 | 0.00 |
| protein-tyrosine kinase activity |  |  | 3 | 0.04 | 5 | 0.00 |
| damaged DNA binding | 1 | 0.02 |  |  |  |  |
| aspartic-type endopeptidase activity |  |  |  |  | 2 | 0.03 |
| inositol-3-phosphate synthase activity | 1 | 0.00 |  |  |  |  |
| protein kinase activity |  |  |  |  | 5 | 0.01 |
| protein tyrosine phosphatase activity |  |  |  |  | 1 | 0.00 |
| ATP binding |  |  |  |  | 6 | 0.02 |
| protein tyrosine/serine/threonine phosphatase activity |  |  |  |  | 1 | 0.01 |
| carbohydrate binding |  |  |  |  | 6 | 3.33E-07 |
| identical protein binding |  |  |  |  | 1 | 0.01 |
