# Supplementary material for: Global RNA sequencing reveals that genotype-dependent allele-specific expression contributes to differential expression in rice F1 hybrids
Source: BMC Plant Biol. 2013 Dec 21;13:221. doi: 10.1186/1471-2229-13-221 (PMC3878109; doi:10.1186/1471-2229-13-221)
Supplement: Additional file 16: Table S12 — Molecular function of preferential allelic expression genes. [file 1471-2229-13-221-S16.docx]

Table S12. Molecular function of preferential allelic expression genes

| **GO Term** | **GL**×**TQ** | | | **GL**×**93-11** | | | | **93-11**×**TQ** | | |
| --- | --- | --- | --- | --- | --- | --- | --- | --- | --- | --- |
|  | **Genes** | | **P value** | | **Genes** | | **P value** | | **Genes** | **P value** |
| nucleotide binding | 252 | 4.38E-48 | | 300 | | 1.27E-66 | | 220 | | 3.26E-30 |
| transcription factor activity | 163 | 1.20E-27 | | 127 | | 3.67E-11 | | 158 | | 2.33E-23 |
| receptor activity | 59 | 3.55E-18 | | 77 | | 2.05E-28 | | 25 | | 0.02 |
| structural molecule activity | 63 | 4.19E-11 | | 89 | | 2.71E-22 | | 59 | | 1.02E-08 |
| transporter activity | 112 | 2.22E-14 | | 105 | | 5.37E-10 | | 119 | | 1.25E-15 |
| protein binding | 231 | 1.09E-55 | | 276 | | 4.19E-76 | | 185 | | 3.07E-29 |
| kinase activity | 180 | 7.10E-37 | | 235 | | 1.27E-62 | | 142 | | 8.13E-18 |
| carbohydrate binding | 7 | 0.03 | | 9 | | 0.00 | | 8 | | 0.01 |
| motor activity | 19 | 1.31E-08 | |  | |  | | 13 | | 2.07E-04 |
| aminomethyltransferase activity |  |  | | 1 | | 0.01 | | 1 | | 0.01 |
| nuclease activity |  |  | | 12 | | 0.02 | | 13 | | 0.01 |
| nucleoside diphosphate kinase activity |  |  | | 4 | | 1.23E-06 | | 4 | | 1.08E-06 |
| sterol carrier activity |  |  | | 1 | | 0.03 | | 1 | | 0.03 |
| ATP binding |  |  | | 35 | | 0.04 | | 38 | | 0.01 |
| arsenite transporter activity | 1 | 0.01 | |  | |  | | 1 | | 0.01 |
| hydrolase activity | 74 | 0.01 | | 94 | | 1.18E-05 | |  | |  |
| adenyl-nucleotide exchange factor activity | 1 | 0.04 | |  | |  | |  | |  |
| DNA binding | 117 | 0.01 | |  | |  | |  | |  |
| damaged DNA binding | 4 | 1.62E-04 | |  | |  | |  | |  |
| GTP cyclohydrolase II activity | 1 | 0.04 | |  | |  | |  | |  |
| NAD+ ADP-ribosyltransferase activity | 1 | 0.03 | |  | |  | |  | |  |
| fumarylacetoacetase activity | 1 | 0.01 | |  | |  | |  | |  |
| glyceraldehyde-3-phosphate dehydrogenase (phosphorylating) activity | 4 | 3.18E-06 | |  | |  | |  | |  |
| dolichyl-diphosphooligosaccharide-protein glycotransferase activity | 1 | 0.01 | |  | |  | |  | |  |
| structural constituent of cytoskeleton | 2 | 0.00 | |  | |  | |  | |  |
| DNA-dependent ATPase activity | 3 | 0.00 | |  | |  | |  | |  |
| lipid binding | 14 | 0.01 | |  | |  | |  | |  |
| protein kinase CK2 regulator activity | 1 | 0.04 | |  | |  | |  | |  |
| 3,4-dihydroxy-2-butanone-4-phosphate synthase activity | 1 | 0.04 | |  | |  | |  | |  |
| oxidoreductase activity, acting on other nitrogenous compounds as donors, iron-sulfur protein as acceptor | 1 | 0.03 | |  | |  | |  | |  |
| protein homodimerization activity | 1 | 0.04 | |  | |  | |  | |  |
| chaperone binding | 1 | 0.04 | |  | |  | |  | |  |
| succinate dehydrogenase activity |  |  | | 1 | | 0.04 | |  | |  |
| beta-1,4-mannosylglycoprotein 4-beta-N-acetylglucosaminyltransferase activity |  |  | | 1 | | 0.03 | |  | |  |
| 2-amino-4-hydroxy-6-hydroxymethyldihydropteridine diphosphokinase activity |  |  | | 1 | | 0.03 | |  | |  |
| GMP synthase (glutamine-hydrolyzing) activity |  |  | | 1 | | 0.01 | |  | |  |
| adenylate cyclase activity |  |  | | 1 | | 0.03 | |  | |  |
| dihydropteroate synthase activity |  |  | | 1 | | 0.03 | |  | |  |
| protein tyrosine phosphatase activity |  |  | | 1 | | 0.04 | |  | |  |
| valine-tRNA ligase activity |  |  | | 1 | | 0.03 | |  | |  |
| calcium ion binding |  |  | | 10 | | 0.00 | |  | |  |
| succinate dehydrogenase (ubiquinone) activity |  |  | | 1 | | 0.03 | |  | |  |
| galactosyltransferase activity |  |  | | 2 | | 0.03 | |  | |  |
| enzyme regulator activity |  |  | | 7 | | 0.01 | |  | |  |
| unfolded protein binding |  |  | | 6 | | 2.60E-04 | |  | |  |
| RNA binding |  |  | |  | |  | | 36 | | 0.00 |
| hydroxymethylglutaryl-CoA synthase activity |  |  | |  | |  | | 1 | | 0.03 |
| trehalose-phosphatase activity |  |  | |  | |  | | 2 | | 0.02 |
| tyrosine-tRNA ligase activity |  |  | |  | |  | | 1 | | 0.01 |
| phosphopantetheinyltransferase activity |  |  | |  | |  | | 1 | | 0.01 |
| electron carrier activity |  |  | |  | |  | | 10 | | 0.03 |
| heme binding |  |  | |  | |  | | 4 | | 0.05 |
