# Supplementary material for: Global RNA sequencing reveals that genotype-dependent allele-specific expression contributes to differential expression in rice F1 hybrids
Source: BMC Plant Biol. 2013 Dec 21;13:221. doi: 10.1186/1471-2229-13-221 (PMC3878109; doi:10.1186/1471-2229-13-221)
Supplement: Additional file 17: Table S13 — Biological function of monoallelic expression genes. [file 1471-2229-13-221-S17.docx]

Table S13. Biological function of monoallelic expression genes

| **GO Term** | **GL**×**TQ** | | **GL**×**93-11** | | **93-11**×**TQ** | |
| --- | --- | --- | --- | --- | --- | --- |
|  | **genes** | **P value** | **genes** | **P value** | **genes** | **P value** |
| protein modification | 27 | 3.10E-11 | 27 | 1.35E-12 | 36 | 9.22E-17 |
| signal transduction | 31 | 3.16E-13 | 27 | 1.48E-11 | 43 | 2.53E-21 |
| response to endogenous stimulus | 28 | 8.58E-10 | 24 | 2.49E-08 | 40 | 6.32E-17 |
| myo-inositol biosynthesis | 1 | 0.001036 |  |  |  |  |
| mismatch repair | 1 | 0.014402 |  |  |  |  |
| amino acid and derivative metabolism | 11 | 9.49E-05 |  |  |  |  |
| phospholipid biosynthesis | 1 | 0.014402 |  |  |  |  |
| carbohydrate metabolism | 11 | 4.67E-06 |  |  | 6 | 0.02499 |
| electron transport | 8 | 7.19E-04 |  |  | 6 | 0.023124 |
| protein folding |  |  | 3 | 1.06E-04 | 3 | 2.26E-04 |
| lipid metabolism | 13 | 4.57E-07 |  |  | 12 | 1.16E-05 |
| biosynthesis | 16 | 1.59E-04 |  |  | 12 | 0.027846 |
| response to abiotic stimulus | 12 | 0.002198 |  |  | 10 | 0.0377 |
| morphogenesis | 4 | 0.005116 |  |  | 6 | 1.47E-04 |
| flower development | 4 | 0.014941 |  |  | 6 | 7.76E-04 |
| secondary metabolism | 8 | 9.45E-07 |  |  | 6 | 2.27E-04 |
| cell differentiation | 9 | 1.90E-06 |  |  | 11 | 6.94E-08 |
| secretory pathway |  |  | 14 | 8.81E-05 | 13 | 0.003522 |
| protein amino acid phosphorylation |  |  |  |  | 5 | 0.006267 |
| physiological process |  |  |  |  | 45 | 0.036896 |
| response to biotic stimulus |  |  |  |  | 15 | 5.52E-05 |
| negative regulation of enzyme activity |  |  |  |  | 1 | 0.003543 |
