# Supplementary material for: Global RNA sequencing reveals that genotype-dependent allele-specific expression contributes to differential expression in rice F1 hybrids
Source: BMC Plant Biol. 2013 Dec 21;13:221. doi: 10.1186/1471-2229-13-221 (PMC3878109; doi:10.1186/1471-2229-13-221)
Supplement: Additional file 18: Table S14 — Biological function of preferential allelic expression genes. [file 1471-2229-13-221-S18.docx]

Table S14. Biological function of preferential allelic expression genes

| **GO Term** | **GL**×**TQ** | | **GL**×**93-11** | | **93-11**×**TQ** | |
| --- | --- | --- | --- | --- | --- | --- |
|  | **genes** | **P value** | **genes** | **P value** | **genes** | **P value** |
| protein modification | 221 | 2.89E-48 | 235 | 4.77E-50 | 175 | 8.30E-24 |
| signal transduction | 238 | 6.76E-50 | 266 | 1.24E-58 | 182 | 1.07E-21 |
| biosynthesis | 125 | 3.00E-10 | 147 | 1.38E-14 | 90 | 0.023505 |
| morphogenesis | 32 | 2.70E-10 | 39 | 7.50E-14 | 23 | 4.21E-05 |
| response to endogenous stimulus | 264 | 1.63E-54 | 265 | 1.75E-48 | 207 | 3.00E-25 |
| DNA metabolism | 49 | 4.49E-05 | 42 | 0.010399 | 46 | 7.96E-04 |
| protein biosynthesis | 40 | 2.48E-06 | 51 | 5.48E-10 | 45 | 8.44E-08 |
| amino acid and derivative metabolism | 86 | 6.11E-15 | 79 | 2.05E-10 | 61 | 3.23E-05 |
| lipid metabolism | 67 | 7.99E-11 | 63 | 5.17E-08 | 45 | 0.003092 |
| response to stress | 172 | 2.38E-24 | 165 | 2.76E-18 | 204 | 5.86E-36 |
| catabolism | 76 | 1.96E-19 | 43 | 3.72E-04 | 58 | 1.06E-09 |
| response to external stimulus | 58 | 5.92E-11 | 56 | 6.79E-09 | 62 | 5.63E-12 |
| response to biotic stimulus | 107 | 1.36E-13 | 143 | 4.41E-26 | 149 | 2.22E-30 |
| response to abiotic stimulus | 141 | 9.70E-23 | 140 | 2.49E-19 | 146 | 4.98E-23 |
| pollination | 10 | 0.001166 | 20 | 2.35E-10 | 11 | 4.48E-04 |
| flower development | 30 | 3.36E-06 | 44 | 1.54E-12 | 42 | 8.04E-12 |
| cell organization and biogenesis | 39 | 1.81E-04 | 33 | 0.019878 | 31 | 0.033566 |
| cell differentiation | 44 | 2.43E-11 | 51 | 4.52E-14 | 36 | 6.69E-07 |
| secretory pathway | 81 | 0.003356 | 93 | 2.74E-04 | 90 | 3.59E-04 |
| carbohydrate metabolism | 50 | 1.69E-06 | 47 | 1.03E-04 |  |  |
| cell cycle | 17 | 4.60E-07 | 9 | 0.024831 |  |  |
| autophagy |  |  | 1 | 0.040854 | 1 | 0.03962 |
| CTP biosynthesis |  |  | 4 | 1.23E-06 | 4 | 1.08E-06 |
| embryonic development |  |  | 7 | 2.71E-06 | 3 | 0.022383 |
| glycine catabolism |  |  | 1 | 0.027425 | 1 | 0.02659 |
| GTP biosynthesis |  |  | 4 | 1.23E-06 | 4 | 1.08E-06 |
| UTP biosynthesis |  |  | 4 | 1.23E-06 | 4 | 1.08E-06 |
| reproduction |  |  | 24 | 0.031612 | 37 | 1.40E-06 |
| response to extracellular stimulus | 12 | 0.001944 |  |  | 12 | 0.002823 |
| transcription | 74 | 3.09E-06 |  |  | 97 | 9.61E-13 |
| photosynthesis | 10 | 3.88E-05 |  |  | 8 | 0.001338 |
| glycolysis | 5 | 6.38E-04 |  |  |  |  |
| electron transport | 55 | 1.40E-08 |  |  |  |  |
| mismatch repair | 3 | 6.83E-04 |  |  |  |  |
| protein folding | 6 | 0.001674 |  |  |  |  |
| tropism | 3 | 3.18E-04 |  |  |  |  |
| secondary metabolism | 27 | 9.70E-07 |  |  |  |  |
| sucrose metabolism |  |  | 1 | 0.013808 |  |  |
| mitochondrial electron transport, succinate to ubiquinone |  |  | 1 | 0.013808 |  |  |
| cAMP biosynthesis |  |  | 1 | 0.027425 |  |  |
| GMP biosynthesis |  |  | 1 | 0.013808 |  |  |
| valyl-tRNA aminoacylation |  |  | 1 | 0.027425 |  |  |
| cell growth |  |  | 2 | 0.017757 |  |  |
| bacteriochlorophyll biosynthesis |  |  | 1 | 0.013808 |  |  |
| transcription initiation |  |  |  |  | 2 | 0.031773 |
| tyrosyl-tRNA aminoacylation |  |  |  |  | 1 | 0.013385 |
| ubiquitin-dependent protein catabolism |  |  |  |  | 4 | 0.008611 |
| phosphatidylserine biosynthesis |  |  |  |  | 1 | 0.013385 |
| apoptosis |  |  |  |  | 9 | 0.003286 |
| antibacterial humoral response (sensu Protostomia) |  |  |  |  | 1 | 0.013385 |
| cell death |  |  |  |  | 17 | 0.006874 |
| trehalose biosynthesis |  |  |  |  | 2 | 0.029 |
